# Supplementary material for: Knowledge, attitude, and preventive practices towards COVID-19 and associated factors among adult hospital visitors in South Gondar Zone Hospitals, Northwest Ethiopia
Source: PLoS One. 2021 May 17;16(5):e0250145. doi: 10.1371/journal.pone.0250145 (PMC8128268; doi:10.1371/journal.pone.0250145)
Supplement: S1 Data — (DOCX) [file pone.0250145.s001.docx]

Part I Socio-demographic information of the respondents

| Part I Socio-demographic information health facility related questions | | | |
| --- | --- | --- | --- |
| Code | Question | Response | |
| 1.1 | Age of the respondents | ------------------------------------ | |
| 1.2 | Sex | 1.Male  2.Female | |
| 1.3 | Marital statuses | 1. single  2. married  3. divorced  4. widowed | |
| 1.4 | Religions | 1. Orthodox  2. protestant  3. Muslim  9. others specify | |
| 1.5 | Educational status | 1. Cannot read and write  2. read and write  3. primary (1-8 grade)  4. secondary(9-12) grade  5. college and above | |
| 1.6 | Occupation | 1.Farmer  2.Student  3.Unemployed  4.Government worker  5.Private business workers  9.Others | |
| 1.7 | Resident | 1.Urban  2.Rural | |
| 1.8 | Monthly income | ------------------------ | |
| 1.9 | Do you have history of chronic medical illness | 1.yes  2.no | |
| 1.10 | Do you take health education on the COVID 19? | 1.yes  2. no | |
| 1.11 | Do you use social media as source of information? | 1.yes  2. no | |
| 1.12 | Do you use peer as source of information? | 1.yes  2. no | |
| 1.13 | Do you use radio /TV as source of information? | 1.Yes  2. no | |
| 1.14 | Do you use religious institution as source of information | 1.yes  2.no | |
| **Part II knowledge related questions** | | | |
| **2.1** | Did you hear about covid 19? | | 1.yes  2.no  3. I do not know |
| **2.2** | COVID 19 is caused by virus. | | 1.yes  2.no  3. I do not know |
| **2.3** | The major clinical symptoms of covid 19 infected person Dry Cough, fever and sore throat and myalgia. | | 1.yes  2.no  3. I do not know |
| **2.4** | Unlike the common cold, stuffy nose, runny nose, and sneezing are less common in persons Infected with the covid-19 virus | | 1.yes  2. no  3.I do not know |
| **2.5** | All persons with COVID 19 may not develop sever cases, only those who are elderly, have chronic illness and obese are more likely to be severe cases. | | 1.yes  2. no  3.I do not know |
| **2.6** | Currently there is no effective cure for covid 19 but early symptomatic and supportive treatments can help most patients recover from the infection. | | 1. yes  2. no  3.I do not know |
| **2.7** | Covid 19 viruses can spreads via respiratory droplets of infected individuals | | 1.yes  2.no  3. I do not know |
| **2.8** | Eating or contacting wild animals would result in the infection by the covid 19 virus | | 1.Yes  2. no  3.I do not know |
| **2.9** | Persons with COVID 19 virus cannot transmit the virus to others when a fever is not present | | 1.Yes  2. no  3.I do not know |
| **2.10** | Proper washing hand with soap and water is one method of preventing COVID-19. | | 1.Yes  2. no  3.I do not know |
| **2.11** | Wearing general masks can prevent one from acquiring infection by the COVID 19 virus | | 1.yes  2. no  3.I do not know |
| **2.12** | It is not necessary for children and young adults to take measures to prevent the infection by Covid 19 virus | | 1.yes  2. no  3.I do not know |
| **2.13** | Individuals should avoid going to crowded places such as bus parks and avoid public transportation to prevent the transmission of COVID-19 | | 1.yes  2. no  3.I do not know |
| **2.14** | People who have contact with someone infected with COVID 19 virus should be immediately isolated in a proper place in general the observation period is 14 days | | 1.yes  2. no  3.I do not know |
| **2.15** | Isolation and treatment of people who are infected with COVID 19 virus are effective ways to reduce the spread of virus | | 1.yes  2. no  3.I don’t know |
|  | **Part III attitude related questions** | |  |
| **3.1** | Black race is protected towards COVID 19 disease. | | 1.agree  2. neutral  3. disagree |
| **3.2** | Wearing a well-fitting face mask are effective in preventing COVID 19 virus | | 1.agree  2. neutral  3. disagree |
| **3.3** | Hand wash can prevent you from COVID 19 virus. | | 1.agree  2.neutral  3.disagree |
| **3.4** | Ethiopia is in the good position to contain COVID 19 virus | | 1.agree  2.neutral  3.disagree |
| **3.5** | If you are infected with COVID-19, do you avoid hiding of your infection? | | 1.agree  2.neutral  3.disagree |
| **3.6** | If you get infected with COVID 19, Do you go to hospital as advised? | | 1.agree  2.neutral  3.disagree |
| **3.7** | Do you think that you can get infected with COVID 19 if you are contacted with an infected patient despite your good immunity? | | 1.agree  2.neutral  3.disagree |
| **3.8** | Do you think that COVID 19 is fatal | | 1.agree  2.neutral  3.disagree |
| **3.9** | During the outbreak of COVID 19 eating well cooked and safely handled meat is safe. | | 1.agree  2.neutral  3.neutral |
| **3.10** | COVID 19 patients should share their recent travel history with health care provider. | | 1.agree  2.neutral  3.disagree |
| **3.11** | The occurrence of Covid-19 is not associated with our sin. | | 1.agree  2.neutral  3.disagree |
|  | **Part IV Practice related question** | | **Response** |
| **4.1** | Do you avoid hand shaking to prevent covid 19? | | 1.Yes  2. no |
| **4.2** | Do you wash your hands with soap and water for 20 seconds repeatedly? | | 1.Yes  2. no |
| **4.3** | If soap and water are not readily available, do you use a hand Sanitizer that contains at least 60% alcohol? | | 1.Yes  2. no |
| **4.4** | Do you wear face masks repeatedly when you leave your home? | | 1.Yes  2. no |
| **4.5** | Do you coughing and sneezing into the elbow or within clothing? | | 1.Yes  2. no |
| **4.6** | In recent days, do you avoid going to any crowded place? | | 1.Yes  2. no |
| **4.7** | Do you avoid eating of raw animal products to prevent COVID 19 virus? | | 1.Yes  2. no |
| **4.8** | Do you avoid touching of your mouth nose and eyes by unwashed hands? | | 1.Yes  2. no |
| **4.9** | Do you keep your self 2m away from the others when you got to public area? | | 1.Yes  2. no |
| **4.10** | Do you stay at your home after the emergent of covid 19? | | 1.yes  2. no |
