# Supplementary material for: Knowledge, attitude, and preventive practices towards COVID-19 and associated factors among adult hospital visitors in South Gondar Zone Hospitals, Northwest Ethiopia
Source: PLoS One. 2021 May 17;16(5):e0250145. doi: 10.1371/journal.pone.0250145 (PMC8128268; doi:10.1371/journal.pone.0250145)
Supplement: S2 Data — (DOCX) [file pone.0250145.s002.docx]

**የፈቃደድ(ስምምነት)**

ሰላም! ስሜ ___________________ እባላለሁ፡፡ የመጣሁት ከደብረታቦር ዩኒቨርሲቲ ሲሆን በተቋሙ እየተካሄደ ያለዉን ጥናት በመወከል የመረጃ ሰብሳቢዎች ቡድን አባል ነኝ ፡፡ የጥናቱ ዓላማ በደቡብ ጎንደር ዞን በሚገኙ የጤና ተቋማትለተለያዩ አገልግሎት በሚመጡ ደንበኞች የኮቪድ 19 ለመከላከል የሚደረጉ ተግባራትና ተያያዥ ጉዳዮችን ለመዳሰስ ያተኮረ ነዉ፡፡ የጥናቱ መረጃ የሚሰበሰብበት መንገድ ፊት ለፊት ቃለ-መጠይቅ ፣ ምልከታን ያካትታል ፡፡ መረጃዉን ለመሰብሰብ የሚወስደዉ ጊዜ ቢበዛ 15 ደቂቃ ይሆናል። የሚያቀርቧቸው መረጃዎች በሙሉ በመለያ ቁጥርዎ( ኮዱን ) ብቻ እንጂ ስም መፃፍ ስለማይችል ለእኔ የሚሰጡት መረጃ ሁሉ በሚስጢር የተጠበቀ ነው ፡፡ በዚህ ምክንያት ፣ ቅን እና እውነተኛ መልስ እንዲሰጡኝ በትህትና እጠይቃለሁ ፡፡ በዚህ ጥናት ውስጥ ያለዎት ተሳትፎ ሙሉ በሙሉ በእርስዎ ፈቃደኝነት ላይ የተመሠረተ ነው ስለሆነም በማንኛውም ጊዜ ከጥናቱ የመውጣት ፣ የመቃወም መብት አለዎት ፡፡ ጥያቄዎችን ለመመለስ መሳተፍ ወይም እምቢ ማለት በህይወትዎ ላይ ምንም ተጽዕኖ አይኖረውም ፡፡ ስለ ጥያቄው እርግጠኛነት ወይም ጥያቄ ካለዎት ወይም የዚህ ጥናት ውጤቶችን ማወቅ ከፈለጉ ዋና ተመራማሪውን ፣ የምርምር ተቆጣጣሪውን ማነጋገር ይችላሉ ፡፡

በጥናቱ ለመሳተፍ ይስማማሉ- 1. አዎ 2. የለም (ከሁለቱ አንደኛው ለቃል ስምምነት ምልክት ያድርጉ) ፊርማዎ ______________________ (በጽሑፍ የተሰጠ ስምምነት)

አማርኛ መጠይቅ

| ክፍል 1 የሶሽዮ-ዲሞግራፊ መረጃ መጠይቅ | | | |
| --- | --- | --- | --- |
| **ተ.ቁ** | **ጥያቄ** | **አማራጭ** | **እለፍ** |
| 1.1 | ዕድሜ | --------------------------- |  |
| 1.2 | ፆታ | 1. ወንድ  2. ሴት |  |
| 1.3 | የጋብቻ ሁኔታ | 1.ያላገባ  2.ያገባ  3.የፈታ  4.የሞተበት |  |
| 1.4 | ሃይማኖት | 1.ኦርቶዶክስ  2. ሙስሊም  3.ፕሮቴስታነት  9. ሌላ ካለ ይግለፁ |  |
| 1.5 | የትምህረት ደረጃ | 1.ማንበብና መጻፍ የማይችል  2. ማንበብና መጻፍ የሚችል  3.አንደኛ ደረጃ(1-8ኛ) ክፍል  4.ሁለተኛ ደረጃ(9-12ኛ) ክፍል  5 ኮሌጅና ከዚያ በላይ. |  |
| 1.6 | የስራ መስክ | 1. ገበሬ  2. ተማሪ  3 ሥራ የሌለዉ  4 የመንግስት ሰራተኛ  5. የግል ሰራተኛ |  |
| 1.7 | መኖርያ ቦታ | 1.ከተማ  2.ገጠር |  |
| 1.8 | የወር ገቢ | --------------------ብር |  |
| 1.9 | የቆየ (ስር የሰደደ በሽታ አለብዎት ? | 1.አለ  2. የለም |  |
| 1.10 | ስለ ኮሮና ስልጠና ወስደዋል? | 1. አለ  2. የለም |  |
| 1.11 | ማህበራዊ ሚድያ እንደመረጃ ምንጭነት ይጠቀማሉ? | 1. አለ  2. የለም |  |
| 1.12 | ቴሌቭዥን /ራዲዮ አንደመረጃ ምንጭነት ይጠቀማሉ ? | 1.አለ  2. የለም |  |
| 1.13 | ከጓደኛዎት/ከዘመድ ስለ ኮቪድ በሽታእንደ መረጃ ምንጭነት ይጠቀማሌ ? | 1.አለ  2. የለም |  |
| 1.14 | ከሀይማኖት ተቋማትስለኮሮና በሽታ እንደመረጃ ምንጭነት ይጠቀማሉ ? | 1.አለ  2. የለም |  |
| **ክፍል II ከእውቀት ጋር የሚዛመዱ ጥያቄዎች** | | | |
| 2.1 | ስለ ኮሮና መረጃ ሰምተህ/ሽ ታዉቃለህ/ሽ | 1.አዎ  2.አይደለም  3.አላዉቅም |  |
| 2.2 | ኮሮና በቫይረስ አማካኝነት የሚመጣ በሽታ ነዉ፡፡ | 1.አዎ  2.አይደለም  3.አላዉቅም |  |
| 2.3 | ሳል ፤ትኩሳት ፤የጉሮሮ መቁሰል፤ የአፍንጫ ፈሳሽ መብዛት፤ የመገጣጠሚያ ህመም እና ተቅማጥ ዋና ዋና የኮሮና ምልክቶች ናቸዉ፡፡ | 1.አዎ  2.አይደለም  3.አላዉቅም |  |
| 2.4 | ከተለመደው ጉንፋን በተቃራኒ ፣ የአፍንጫ መታፈን ፣ የአፍንጫ ፈሳሽ መብዛት እና ማስነጠስ በኮቪድ -19 ቫይረስ በተያዙ ሰዎች ብዙም የተለመዱ አይደሉም፡፡ | 1.አዎ  2.አይደለም  3.አላዉቅም |  |
| 2.5 | ሁሉም በኮረና ቫይረስ የተያዙ ሰዎች ለከፋ ችግር አይጋለጡም ነገር ግን ፣ በዕድሜ የገፉ ሰዎች ፣ ሥር የሰደደ ህመም እና ከመጠን በላይ የሆነ ዉፍረት ያላቸዉ ሰዎች የመጠቃት ዕድላቸው ከፍተኛ ነው፡፡ | 1.አዎ  2.አይደለም  3.አላዉቅም |  |
| 2.6 | በአሁኑ ጊዜ ለኮቪድ 19 ውጤታማ የሆነ መድኃኒት የለም ነገር ግን ቀደም ሲል የበሽታ ምልክቶች እና ድጋፍ ሰጪ ህክምናዎች አብዛኛዎቹ ህመምተኞች ከበሽታው እንዲድኑ ሊያግዝ ይችላል ፡፡ | 1.አዎ  2.አይደለም  3.አላዉቅም |  |
| 2.7 | የ ኮቪድ 19 ቫይረሶች በበሽታው በተያዙ ግለሰቦች የመተንፈሻ አካላት ጠብታዎች በኩል ሊሰራጭ ይችላል:: | 1.አዎ  2.አይደለም  3.አላዉቅም |  |
| 2.8 | የዱር እንስሳትን መብላት ወይም መገናኘት ኮቪድ-19 ቫይረስ በሽታን ያስከትላል፡፡ | 1.አዎ  2.አይደለም  3.አላዉቅም |  |
| 2.9 | በ ኮቪድ 19 ቫይረስ የተያዙ ሰዎች ትኩሳት በማይኖርበት ጊዜ ቫይረሱን ወደ ሌሎች ሰዎች አያስተላልፉም፡፡ | 1.አዎ  2.አይደለም  3.አላዉቅም |  |
| 2.10 | እጅን በዉሃና ሳሙና መታጠብ ከኮሮና ቫይረስ ለመከላከል ይጠቅማል፡፡ | 1.አዎ  2.አይደለም  3.አላዉቅም |  |
| 2.11 | የሕክምና ጭምብሎችን መልበስ በ ኮቪድ 19 ቫይረስ በሽታ እንዳንያዝ ይከላከላል | 1.አዎ  2.አይደለም  3.አላዉቅም |  |
| 2.12 | በ ኮቪድ-19 ቫይረስ በሽታን ለመከላከል ለልጆች እና ለወጣቶች አስፈላጊ እርምጃዎችን መውሰድ አስፈላጊ አይደለም፡፡ | 1.አዎ  2.አይደለም  3.አላዉቅም |  |
| 2.13 | በ ኮቪድ 19 ቫይረስ በሽታን ለመከላከል እንደ አውቶቡስ መናኸሪያ ባሉ ብዙ ሰዎች ወደሚኖሩባቸው ቦታዎች ከመሄድ መቆጠብ እና የህዝብ መጓጓዣን ማስቀረት አለባቸው፡፡ | 1.አዎ  2.አይደለም  3.አላዉቅም |  |
| 2.14 | በኮቪድ 19 ቫይረስ ከተያዘው ሰው ጋር ንክኪ ያላቸው ሰዎች ወዲያውኑ በታዩበት ጊዜ ለ14 ቀናት ውስጥ በተገቢው ቦታ ተለይተው መቆየት አለባቸው ፡፡ | 1.አዎ  2.አይደለም  3.አላዉቅም |  |
| 2.15 | በ ኮቪድ 19 ቫይረስ የተያዙ ሰዎችን መለየት እና ሕክምና መስጠት የቫይረሱን ስርጭት ለመቀነስ ውጤታማ መንገዶች ናቸው | 1.አዎ  2.አይደለም  3.አላዉቅም |  |
|  | **ክፍል 3፡ ግንዛቤ/ አመለካከት ተሃማጅ ጥቄዎች** | **ምላሽ** |  |
| 3.1 | ጥቁሮች ከነጮች የበለጠ በ ኮቪድ 19 በሽታ ይጠበቃሉ? | 1.እስማማለሁ  2.አልስማማም  3.ገለልተኛ |  |
| 3.2 | በደንብ የተጣጣመ የፊት ጭንብል በመልበስ የኮቪድ 19 ቫይረስን ለመከላከል ውጤታማ ነው፡፡ | 1.እስማማለሁ  2.አልስማማም  3.ገለልተኛ |  |
| 3.3 | እጅን መታጠብ ከ ኮቪድ 19 ቫይረስ ይከላከል ብለዉ ያስባሉ፡፡ | 1.እስማማለሁ  2.አልስማማም  3.ገለልተኛ |  |
| 3.4 | ኢትዮጵያ የኮቪድ19 ቫይረስ ለመቆጣጠር በጥሩ አቋም ላይ ትገኛለች፡፡ | 1.እስማማለሁ  2.አልስማማም  3.ገለልተኛ |  |
| 3.5 | የኮቪድ በሽታን በሽታየን መደበቅ የለብኝም ብለዉ ያስባሉ፡፡ | 1.እስማማለሁ  2.አልስማማም  3.ገለልተኛ |  |
| 3.6 | በ ኮቪድ 19 ቫይረስ ከተያዝኩ ወድያዉኑ ወደ ሆስፒታል እሄዳለሁ ብለዉ ያስባሉ ፡፡ | 1.እስማማለሁ  2.አልስማማም  3.ገለልተኛ |  |
| 3.7 | ምንም እንኳን ጥሩ በሽታ የመከላከል አቅም ቢኖረኝም በበሽታው ከተጠቁ በሽተኞች ጋር ከተገናኘሁ በ ኮቪድ 19 ልያዝ እችላለሁ ብለዉ ያስባሉ ፡፡ | 1.እስማማለሁ  2.አልስማማም  3.ገለልተኛ |  |
| 3.8 | ኮቪድ 19 ገዳይ በሽታ ነው ብለዉ ያስባሉ፡፡ | 1.እስማማለሁ  2.አልስማማም  3.ገለልተኛ |  |
| 3.9 | ኮቪድ 19 በሚከሰትበት ጊዜ በጥሩ ሁኔታ የተቀቀለ እና የተቀመጠ ሥጋ መብላት አስፈላጊ ነው ብለዉ ያስባሉ ፡፡ | 1.እስማማለሁ  2.አልስማማም  3.ገለልተኛ |  |
| 3.10 | ኮቪድ 19 ሕመምተኞች የቅርብ ጊዜ የጉዞ ታሪካቸውን ለጤና ባለሙያዎች ማጋራት አለባቸው ብለዉ ያስባሉ ፡፡ | 1.እስማማለሁ  2.አልስማማም  3.ገለልተኛ |  |
| 3.11 | የኮሮና በሽታ በሰዎች ሃጥያት አማካኝነት የመጣ አይደለም ብለዉ ያምናሉ;፡ | 1.እስማማለሁ  2.አልስማማም  3.ገለልተኛ |  |
|  | **ክፍል 4፡ ትግበራ ተዛማጅ ጥቄዎች** | **ምላሽ** |  |
| 4.1 | የኮቪድ 19 ን ለመከላከል እጅን ሰላምታን አስወገደዋል? | 1.አዎ  2.የለም |  |
| 4.2 | ማነኛዉንም ነገር ከመንካትዎ በፊት እጅዎን በተደጋጋሚ በዉሃና በሳሙና ይታጠባሉ? | 1.አዎ  2.የለም |  |
| 4.3 | ውሃ እና ሳሙና የማይገኝ ከሆነ አልኮል ያለዉ ሳኒታይዘር ይጠቀማሉ? | 1.አዎ  2.የለም |  |
| 4.4 | የፊት ጭምብሎችን በተደጋጋሚ ያደርጋሉ? | 1.አዎ  2.የለም |  |
| 4.5 | በሚስሉበትና በሚያስነጥሱብት ጊዜ ለማስወገድ የቆሻሻ በተዘጋጀ እቃ በአግባቡ ያስወግዳሉ? | 1.አዎ  2.የለም |  |
| 4.6 | ከቅርብ ቀናት ወዲህ ወደተጨናነቀ ቦታ ሄደዉ ያዉቃሉ? | 1.አዎ  2.የለም |  |
| 4.7 | COVID 19 ቫይረስን ለመከላከል ጥሬ የእንስሳት ምርቶችን ከመብላት ይቆጠባሉ? | 1.አዎ  2.የለም |  |
| 4.8 | እጅዎን ሳይታጠቡ አፍንጫ፣አይንና አፍዎን ከመንካት ተቆጥበዋል? | 1.አዎ  2. የለም |  |
| 4.9 | ወደ ህዝብ በሚሄዱበት ጊዜ እራስዎን ከሌላው ሰው 2 ሜትር ይቆዩ ነበር? | 1.አዎ  2. የለም |  |
| 4.10 | የኮሮና በሽታ ከተከሰተ በኁላ ያለበቂ ምክንያት ከቤት ከመዉጣት ተቆጥበዋል? | 1.አዎ  2. የለም |  |

**ለተሳትፎዎ ከልብ አመሰግናለሁ**
