# Supplementary material for: Knowledge, attitude, and preventive practices towards COVID-19 and associated factors among adult hospital visitors in South Gondar Zone Hospitals, Northwest Ethiopia
Source: PLoS One. 2021 May 17;16(5):e0250145. doi: 10.1371/journal.pone.0250145 (PMC8128268; doi:10.1371/journal.pone.0250145)
Supplement: Attachment — Submitted filename: PONE-D-20-36453_reviewer.pdf [file pone.0250145.s005.pdf]

PLOS ONE

Preventive Practice and Associated Factors towards COVID-19 among Medical Visitors in Hospitals of South Gondar Zone, Northwest Ethiopia

--Manuscript Draft--

**Manuscript Number:**	PONE-D-20-36453
**Article Type:**	Research Article
**Full Title:**	Preventive Practice and Associated Factors towards COVID-19 among Medical Visitors in Hospitals of South Gondar Zone, Northwest Ethiopia
**Short Title:**	Preventive practice of COVID-19 among medical visitors

**Corresponding Author:**	Gete Gelaw Berihun, MSc<br>Wollo University<br>Dessie, ETHIOPIA

**Keywords:**	knowledge, attitude, practice visitors, COVID-19

Abstract:

Background : Coronavirus 2019 (COVID-19) is a rapidly expanding respiratory disease of the [illegible]rld. The first case of the disease was reported in Wuhan city, Hubei province, China at the end of late December. Healthcare facilities are the most vulnerable area for the transmission of the disease and medical visitors are members of it.

Objective: To assess preventive practice and associated factors towards COVID-19 among medical visitors in hospitals of South Gondar Zone, Northwest Ethiopia ~~in Augest~~ 2020.

Methods: A facility-based cross-sectional study design was applied from Augoust 1 to 30, 2020 among 404 medical visitors using a systematic random sampling technique. Data were collected using a pre-tested ~~face to face~~ administered questionnaire. Data were cleaned and entered into Epi data version 3.1 and exported to SPSS Version.25 for statistical analysis. Bivariate logistic regression analysis was done to determine the presence and degree of association between preventive practice and socio-demographic variables. Odds Ratio (OR) with 95% confidence intervals (CI) with p values of less than 0.25 was selected as candidate variables for multivariable logistic regression. Multivariable logistic regression analysis was applied for preventive practice and a statistical significance test was declared at P-value <0.05 and adjusted Odd Ratio (AOR) with 95% CI.

Results: ~~A total of 404 participants completed the survey questionnaire~~. Of the participants, 280 (69.3%), 253 (62.6%), 205 (49.3%) have had good knowledge, positive attitude, and good practice respectively towards the prevention of COVID-19. Multivariable logistic regression indicated that educational status of reading and write (AOR=2.78; 95% CI: 1.18, 6.56); college and above (AOR=6.15; 95% CI: 2.18-17.40), and use of social media (AOR=2.96; 95% CI: 1.46, 6.01) were significantly associated with knowledge of COVID-19. Educational status of primary (AOR=6.49; 95% CI: 1.52-27.78) college and above (AOR=6.91; 95% CI2.59-14.50 presence of chronic illness (AOR=5.00; 95% CI; 1.71-14.67), training (AOR=3.91; 95% CI: 1.96-7.70), and peer as a source of information (AOR=2.45; 95% CI: 1.06-5.63) were significantly associated with attitude of COVID-19. Occupation of being a student (AOR=7.70; 95% CI: 1.15-15.86) and being good knowledge (AOR=4.49; 95% CI: 2.41-8.39) was significantly associated with prevention practice of COVID-19.

Conclusion: The findings of this study showed that relatively good knowledge and attitude, however, there was a problem in prevention practice. Most of the participants had reckless practices in the keeping physical distance and the problem of staying at home. Therefore, information dissemination should be conducted on regular basis by health care professionals, and the use of mass media at the entrance of each institution towards the prevention practice of the pandemic.

Order of Authors:

Gete Gelaw Berihun, MSc

Zebader Walle Belete

Awoke Asfaw Keleb

Ayechew Tesema Ademas

Powered by Editorial Manager® and ProduXion Manager® from Aries Systems Corporation

Additional Information:	
**Question**	**Response**
**Financial Disclosure**<br><br>Enter a financial disclosure statement that describes the sources of funding for the work included in this submission. Review the submission guidelines for detailed requirements. View published research articles from *PLOS ONE* for specific examples.<br><br>This statement is required for submission and **will appear in the published article** if the submission is accepted. Please make sure it is accurate.<br><br>**Unfunded studies**<br>Enter: *The author(s) received no specific funding for this work.*<br><br>**Funded studies**<br>Enter a statement with the following details:<br>• Initials of the authors who received each award<br>• Grant numbers awarded to each author<br>• The full name of each funder<br>• URL of each funder website<br>• Did the sponsors or funders play any role in the study design, data collection and analysis, decision to publish, or preparation of the manuscript?<br>• **NO** - Include this sentence at the end of your statement: *The funders had no role in study design, data collection and analysis, decision to publish, or preparation of the manuscript.*<br>• **YES** - Specify the role(s) played.<br><br>* typeset	The author(s) received no specific funding for this work
**Competing Interests**<br><br>Use the instructions below to enter a competing interest statement for this submission. On behalf of all authors, disclose any competing interests that could be perceived to bias this work—acknowledging all financial support and any other relevant financial or non-financial competing interests.	The authors have declared that no competing interests exist.

This statement **will appear in the published article** if the submission is accepted. Please make sure it is accurate. View published research articles from *PLOS ONE* for specific examples.<br><br>**NO authors have competing interests**<br><br>Enter: *The authors have declared that no competing interests exist.*<br><br>**Authors with competing interests**<br><br>Enter competing interest details beginning with this statement:<br><br>*I have read the journal's policy and the authors of this manuscript have the following competing interests: [insert competing interests here]*<br><br>* typeset	
**Ethics Statement**<br><br>Enter an ethics statement for this submission. This statement is required if the study involved:<br><br>• Human participants<br>• Human specimens or tissue<br>• Vertebrate animals or cephalopods<br>• Vertebrate embryos or tissues<br>• Field research<br><br>Write "N/A" if the submission does not require an ethics statement.<br><br>General guidance is provided below. Consult the submission guidelines for detailed instructions. **Make sure that all information entered here is included in the Methods section of the manuscript.**	Ethical clearance was obtained from the ethical review committee of Debre Tabor University, research, and community service coordinator office. Afterward, consent was obtained from the respective hospital managers of the study site. Informed consent was obtained from each participant after explaining the objective of the study.

**Format for specific study types**<br><br>**Human Subject Research (involving human participants and/or tissue)**<br>• Give the name of the institutional review board or ethics committee that approved the study<br>• Include the approval number and/or a statement indicating approval of this research<br>• Indicate the form of consent obtained (written/oral) or the reason that consent was not obtained (e.g. the data were analyzed anonymously)<br><br>**Animal Research (involving vertebrate animals, embryos or tissues)**<br>• Provide the name of the Institutional Animal Care and Use Committee (IACUC) or other relevant ethics board that reviewed the study protocol, and indicate whether they approved this research or granted a formal waiver of ethical approval<br>• Include an approval number if one was obtained<br>• If the study involved *non-human primates*, add *additional details* about animal welfare and steps taken to ameliorate suffering<br>• If anesthesia, euthanasia, or any kind of animal sacrifice is part of the study, include briefly which substances and/or methods were applied<br><br>**Field Research**<br><br>Include the following details if this study involves the collection of plant, animal, or other materials from a natural setting:<br>• Field permit number<br>• Name of the institution or relevant body that granted permission	
**Data Availability**<br><br>Authors are required to make all data underlying the findings described fully available, without restriction, and from the time of publication. PLOS allows rare exceptions to address legal and ethical concerns. See the PLOS Data Policy and FAQ for detailed information.	No - some restrictions will apply

Powered by Editorial Manager® and ProduXion Manager® from Aries Systems Corporation

A Data Availability Statement describing where the data can be found is required at submission. Your answers to this question constitute the Data Availability Statement and **will be published in the article**, if accepted.<br><br>**Important:** Stating 'data available on request from the author' is not sufficient. If your data are only available upon request, select 'No' for the first question and explain your exceptional situation in the text box.<br><br>Do the authors confirm that all data underlying the findings described in their manuscript are fully available without restriction?	
**Describe where the data may be found in full sentences. If you are copying our sample text, replace any instances of XXX with the appropriate details.**<br><br>• If the data are **held or will be held in a public repository**, include URLs, accession numbers or DOIs. If this information will only be available after acceptance, indicate this by ticking the box below. For example: *All XXX files are available from the XXX database (accession number(s) XXX, XXX.*).<br>• If the data are all contained **within the manuscript and/or Supporting Information files**, enter the following: *All relevant data are within the manuscript and its Supporting Information files.*<br>• If neither of these applies but you are able to provide **details of access elsewhere**, with or without limitations, please do so. For example:<br><br>*Data cannot be shared publicly because of [XXX]. Data are available from the XXX Institutional Data Access / Ethics Committee (contact via XXX) for researchers who meet the criteria for access to confidential data.*<br><br>*The data underlying the results presented in the study are available from (include the name of the third party*	the data is available with corresponding author upon request

and contact information or URL).<br>• This text is appropriate if the data are owned by a third party and authors do not have permission to share the data.<br><br>* typeset	
Additional data availability information:	

Powered by Editorial Manager® and ProduXion Manager® from Aries Systems Corporation

Manuscript
Click here to access/download;Manuscript;final revised patient manucript.docx

COVID-19 Preventive Practice and Associated Factors among Visitors in Hospitals of South Gondar Zone, Northwest Ethiopia

Gete Berihun[1*] **Zebader Walle Belete**[2] **Awoke Keleb**[1] **Ayechew Ademas**[1]

[1]Department of Environmental Health, College of Medicine and Health Sciences, Wollo University, Dessie, Ethiopia.

[2]Department of Public Health, College of Health Sciences, Debre Tabor University, Debre Tabor, Ethiopia.

[*] **corresponding author** **geteberihun@gmail.com**

Abstract

Background: Coronavirus 2019 (COVID-19) is currently the critical health problem of the globe, including Ethiopia. The first case of the disease was reported in Wuhan city, Hubei province, China, in late December 2019. Visitors of healthcare facilities are the high-risk groups due to the presence of suspected and confirmed cases of COVID-19. Even if many trials were conducted on the vaccine and treatment of the disease, still there is no effective treatment and vaccine. Therefore, prevention is the most effective method of reducing the transmission of the disease.

Objective: To assess COVID-19 preventive practice and associated factors among visitors in hospitals ofSouth Gondar Zone, Northwest Ethiopia

Methods: A facility-based cross-sectional study design was applied in August 2020 in visitors of hospitals of South Gondar zone, Northwest Ethiopia. A total of 404 visitors participated in the study and were selected using a systematic random sampling technique. The questionnaire was pre-tested in 5% of the final sample size to establish the validity of the data collection instrument. The data were collected using face-to-face interviews by considering physical distancing and wearing of face masks. The data was entered in Epi-data version 3.1 and exported to Statistical Package for Social Science (SPSS) Version 25 for analysis. Bi-variate Crude Odd Ration (COR) with 95% confidence intervals (CI) and p-values of less than 0.25 were applied to select candidate variables for multi-variable analysis. Then, multi-variable Adjusted Odd Ratio (AOR) using binary logistic regression analysis at a p-value of less than 0.05 at 95% CI was

statistically significance with the preventive practice of COVID-19 in visitors of hospitals ofSouth Gondar zone Northwest Ethiopia.

Results: A total of 404 participants completed the survey questionnaire with a response rate of 95.7%. About 280 (69.3%) had good knowledge, 253 (62.6%) had a positive attitude, and 205 (49.3%) had good preventive practice towards COVID-19. Multi-variable (AOR) logistic regression analysis indicated that educational status who can read and write (AOR=2.78; 95% CI: 1.18, 6.56); college and above (AOR=6.15; 95% CI: 2.18-17.40), and use of social media (AOR=2.96; 95% CI: 1.46, 6.01) were significantly associated with knowledge of COVID-19. Educational status of primary (AOR=6.49; 95% CI: 1.52-27.78) college and above (AOR=6.91; 95% CI 2.59-14.50), presence of chronic illness (AOR=5.00; 95% CI; 1.71-14.67), training on COVID-19 (AOR=3.91; 95% CI: 1.96-7.70), and peer/family as a source of information (AOR=2.45; 95% CI: 1.06-5.63) were significantly associated with attitude of COVID-19. Being a student (AOR=7.70; 95% CI: 1.15-15.86) and good knowledge on COVID-19 (AOR=4.49; 95% CI: 2.41-8.39) were significantly associated with prevention practice of COVID-19.

Conclusion: we concluded that only half of the visitors had a good preventive practice of COVID-19**.** Most of the participants reported that do not comply with the recommended physical distance and staying at home. Therefore, the zonal health office should focus on collaboration with other concerned stakeholders to enhance awareness, attitude, and prevention practice of COVID-19. Furthermore, the prevention and control strategy should be incorporated with health extension programs to address all members of the community mainly rural areas.

Keywords: knowledge, attitude, practice, COVID-19

Introduction

COVID-19 is a rapidly emerging pandemic respiratory disease caused by a novel Coronavirus of Severe Acute Respiratory Syndrome COV-2. Initially, the disease was reported in Wuhan city, Hubei Province, China at the end of December 2019 (1–3). Later on, at the end of January 2020, World Health Organization (WHO) announces the disease as a public health emergency of international concern and then declared it as a global pandemic on March 11 (4–6). Two days later, the government of Ethiopia reported the first confirmed case of COVID -19 (7,8).

COVID-19 transmits mainly through droplets, airborne transmission, and contact between humans (6,9–11). The major sign and symptoms of COVID-19 cases are fever, dry cough, fatigue, myalgia, shortness of breath, and dyspnoea (4–6). The Severe cases of COVID-19 can lead to cardiac injury, respiratory failure, acute respiratory distress syndrome, and death. Elders and patients with chronic medical illnesses like hypertension, cardiac disease, lung disease, cancer, or diabetes have been identified as potential risk factors for disease severity and mortality. Even though the disease has no effective cure, but early recognition of symptoms and timely seeking of supportive care enhance recovery from the illness. The case fatality rate of the disease is approximately 3.4% (6,11).

According to the Worldometer report, as of October 6, 2020, 9:54 am, COVID-19 spreads to more than 214 countries across the world. A total of 35,707,844 confirmed cases were reported. Of them, 26,907,997 recovered and 1,049,700 died of the pandemic (12). The total confirmed cases of COVID-19 in Ethiopia were 79,437; 1,230 died, and 34,016 recovered (13). The pandemic of COVID-19 terrifies the whole population of the entire continent. It disrupts the communities physical, mental health, political, economic, and social stability of countries. The

pandemic also threatened the countries economic growth, sense of security, health care system, trade relations, tourism, employment, and global interactions (14).

Due to the absence of cure (6,11) prevention is recommended as the only strategy to protect against the spread of the pandemic. Universal source control, respiratory hygiene, early identification and isolation of patients with suspected disease, hand washing, social distancing, use of Personal Protective Equipment (PPE), and environmental disinfection are the most common prevention methods of COVID-19 (6,15–18). As a result, the WHO designed different guidelines and online training programs to alleviate the problems (19). But still, there is a deficiency of information on its distribution, transmission, treatment, and prevention of the pandemic (6,18).

The government of Ethiopia has also implemented different prevention measures since the first case of COVID-19. Later on, the country declares and enforces a state of emergency for about six months starting from early March 2020 (20). However, COVID-19 cases are increasing at an alarming rate. Therefore, assessing knowledge is important in identifying gaps and strengthening ongoing prevention methods of the pandemic (4)**.** Even though there are strong initiatives and recognition of the public health importance of COVID-19, it needs strong reinforcements on community prevention practice (11)**.**

The state of emergency towards COVID-19 prevention was terminated at the end of September 2020. As a result, most populations perceived as the disease was eliminated during the termination of a state of emergency. As a result, COVID-19 prevention measures being neglected and confirmed cases of COVID-19 increased from time to time. Even if many studies were conducted on different aspects of the COVID-19 pandemic in Ethiopia, but scientific

evidence on prevention practice of COVID-19 is rare among visitors of healthcare settings. Therefore, the study was designed to assess COVID-19 prevention practice and associated factors among visitors in hospitals ofSouth Gondar Zone, Northwest Ethiopia.

Methods and Materials

Study design, period, and setting

A facility-based cross-sectional study design was applied among visitors in hospitals of South Gondar zone, Northwest Ethiopia from August 1 to 30, 2020. South Gondar zone is one of the 15 administrative zones in the Amhara regional state of Ethiopia. Debre Tabor is its capital town which is located at 597 km to the North of Addis Ababa and 105 km from Bahir Dar which is the capital city of Amhara regional state. According to the population projection of 2014, the total population of the study area was 2,364,603 of which 1,196,318 were males while 1,168,285 were females (population projection 2014). South Gondar zone has a total of eight hospitals of which seven are district hospitals while Debre Tabor hospital is a general hospital (23).

Source population and inclusion criteria

All visitors in hospitals of the South Gondar zone were the source population while all visitors in two randomly selected hospitals (Debre Tabor and Mekane Eyesus) were the study population. Visitors who were less than 18 years old and those who were unable to respond due to illness during data collection were excluded from the study.

Sample size estimation and sampling methods

The sample size was determined using the single population proportion formula by taking the following assumptions.

$$n = \frac{(z_{\alpha/2})^2 * p(1-p)}{d^2}$$

$\boldsymbol{Z_{\alpha/2}}$ is the standard normal variable value at (1-α)% confidence level (α is 0.05 with 95% CI, $Z_{\alpha/2}$ = 1.96), an estimate of the proportion of knowledge attitude and practice, was considered as 50% as there was no similar studies conducted and margin of error, 5%. The sample size became 384 and adding 10% non-response rates so that the final sample size becomes 422.

Initially, two hospitals were selected using a simple random sampling technique by lottery method. The patient flow data were estimated by reviewing the patients' logbook in the last three months and the average number of the patient for a month was calculated to determine the interval. Then, we used a systematic random sampling technique to select study participants of the study. Then, the proportional allocation was carried out to determine the number of participants from each hospital.

Outcome and explanatory variables

The outcome variable was the preventive practice of COVID-19 (good/poor), knowledge (good/poor), and attitude (good/poor), while explanatory variables comprised of socio-demographic variables, behavioral variables, pre-existing medical condition, and sources of information towards COVID-19.

Operational definitions

Knowledge was measured by using 15 questions consisting of signs and symptoms, risk groups and prognosis, method of transmission, and /preventive methods. Each question was responded as either yes, no, and I do not know. Respondents who answered correctly were given 1 point

while others were given 0 points. The total knowledge score ranges from 0-15 and a cut-off level of ≥12 (80% and above) was considered as **good knowledge** while <12 (80%) was considered as **poor knowledge** (24)**.** The attitude section was measured by using 11 items and the response was categorized based on 3 scale measurements with agree (3 points), neutral (2 points), and disagree (1 point). The score of attitude varies from 11 to 33, with an overall score of ≥26.4 (80%) was considered as a **positive attitude (27)**. The prevention practice was measured using 10 items and those who respond as yes were given 1 point while no was marked as 0. The total prevention practice score ranges from 0-10 and a score with a cut-off ≥ 8 (80%) was considered as **good practice** while <8 was taken as a poor practice (25,26).

Data collection, management, and quality assurance

The data were collected using a structured questionnaire which was adapted from articles published in different journals and the WHO guidelines (28–33). The questionnaire consists of four sections including; part I: socio-demographic characteristics of the participants; part II; sources of information towards COVID-19; part III: knowledge of the participants; part IV: Attitude of the participants; and part V: Prevention practice of COVID-19. The tool was prepared in the English version and translated to Amharic version (local language), and re-translated back to English to ensure consistency. A pre-test was conducted using 5% (21) of the final sample size in the Andabet district to establish the validity of the questionnaire and amendment was made accordingly. The data was collected by using face-to-face-interviews by considering COVID-19 prevention measures such as wearing of face-mask and keeping a 2-meter distance from the respondents. The data was collected by four BSc nurse professionals and supervised by two master's degree holders in public health. Two days of training was given for data collectors and supervisors on the overall aim of the study, contents of the tool. Supervision

was carried out on daily basis, and appropriate corrections were done accordingly. Furthermore, double data entry was done to control data entry errors and data cleaning was carried before statistical analysis. The reliability coefficient of Cronbach's alpha was 0.76 which is an acceptable range.

Statistical analysis

Epi-Data version 3.1 was used for data entry and exported to the Statistical Package of the Social Science (SPSS) version 25.0 for statistical analysis. Bi-variate analysis with (COR) and multi-variable analysis (AOR) was determined using binary logistic regression analysis with 95% CI. In the bivariate analysis, variables with p <0.25 were a candidate for multi-variable analysis. From the multi-variable logistic regression analysis, variables with a significance level of p <0.05 were taken as statistically significant and independently associated with outcome variables. The presence of multi-collinearity among independent variables was checked using standard error at the cutoff value of 2 with a maximum standard error of 0.97 and model fitness was checked using the Hosmer-Lemeshow test with a p-value of 0.65.

Result

Socio-demographic characteristics of the respondents

A total of 404 visitors participated in the study with a response rate of 95.7%. The majority of the respondents 242 (59.7%) were females, less than a quarter 92 (22.8%) were in the age of 30-39, more than three fourth 331(81.9%) of the study participants were orthodox, and 295(73.1%) were married. Furthermore, 66 (16.3%), 68(16.8%), and 117(29.0%) of the respondents can't read and write, farmers, and live in rural areas respectively. Similarly, 137 (39.9%) of the participant took training towards COVID-19 and 252 (62.4%) used social media as a source of information for COVID-19. Finally, 56 (13.9%) of the participants had either or more chronic medical illness history **(Table 1).**

Knowledge of medical visitors towards COVID-19

The finding of the study showed that 388 (96.0%) of the participants heard about COVID-19 from different sources of information. But a lower number of respondents 322 (79.7%) knew as COVID-19 is a viral disease while 339 (83.9%) of the respondents knew the major sign and symptoms of COVID-19 cases. Furthermore, 320 (79.2%) participants knew that elders, those who had a chronic medical illness, and obese are more likely to have severe cases of COVID-19. Similarly, 283 (70%) of the respondents knew that COVID-19 can be transmitted from one person to another even in the absence of COVID-19 **(Table 1).**

Attitude of visitors towards COVID-19

The finding of the study also showed that almost half of 203(50.2%) of the participants agree that the black race is not protective against COVID-19. Similarly, 180 (44.6%) of the participants agreed that Ethiopia is in a good position to contain the spread of the COVID-19. Approximately, two-thirds 274 (67.8%) of the participants believed COVID-19 do not cause stigma. This finding also indicated that more than half 221(54.7%) of the respondents agree that they can get infected with COVID-19 if they contacted infected patients despite their good immunity. On the other hand, 55 (13.6%) of the respondents believed that COVID-19 has occurred as a result of our sin **(Table 3).**

Preventive practice of COVID-19

This finding showed that 378 (93.6%) of the participants washed their hands with water and soap for at least 20 seconds and three-fourth of the participants used alcohol-based hand rub sanitizers which contain at least 60% alcohol during the absence of handwashing facilities like water and soap. Furthermore, almost nine out of ten respondents avoid handshaking practice after the emergent of COVID-19. But a relatively lower number of 338 (83.7%) participants used facemasks when they leave their home and 333 (82.4%) practiced respiratory hygiene while coughing and sneezing. Keeping social distancing was one of the major problems faced by the respondents and only 177(43.8%) respondents practiced it. Staying at home after the emergent of COVID-19 was also another challenge and only less than one-third 121(30%) of the participants applied it as a means of COVID-19 prevention measures **(Table 4).**

Status of Knowledge, attitude, and preventive practice of COVID-19

The finding of the study revealed seven out of ten 280 (69.3%) respondents had good knowledge towards COVID-19 while 253 (62.6%) had a positive attitude towards COVID-19. The finding of the study showed the pillar of prevention practice was much lower and only half 199(49.3%) of the participants had a score of good prevention practice of COVID-19 **(Figure 1).**

Predictors of Knowledge, Attitude, and preventive practice towards COVID-19

The multi-variable analysis indicated that educational status and use of social media as a source of information were statistically significant with the knowledge of COVID-19. The finding revealed that those who can read and write were 2.78 times more likely to have good knowledge than those who can‘t read and write. Similarly, those who have college and above educational level were 6.15 (2.18-17.40) times more likely to have good knowledge than those who can't read and write. Additionally, respondents who used social media as a source of information was 2.96 (1.46-6.01) times more likely to have good knowledge than the corresponding group of those who did not use social media **(Table 5).**

The multi-variable analysis revealed that those who had primary education were 6.49 (1.52-27.78) times more likely to have a positive attitude than those who can’t read and write while college above was 6.91 (2.58-14.5) times more likely to have a positive attitude than the corresponding reference group. Medical visitors who had chronic medical illnesses were 5.00 (1.71-14.67) more likely to have a positive attitude than those who don’t have a chronic illness. Respondents who took training on COVID-19 were 3.9 (1.96-7.70) more likely to have a positive attitude than those who didn’t take the training.

Additionally, participants who used peer and family as a source of information were 2.45 (1.06-5.63) times more likely to have a positive attitude than those who didn't exchange information from their peers and families **(Table 6).**

The finding also indicates that being a student was 7.70 (1.15-51.86) more likely to have a good prevention practice as compared to farmers. Similarly, having good knowledge was 4.49 (2.41-8.39) times more likely to have a good prevention practice than those who had poor knowledge **(Table 7).**

Discussion

The pandemic of COVID-19 is still the critical concern of the globe including our country Ethiopia. But up to date, there is no confirmed treatment for the pandemic. Therefore prevention is the single most important method of alleviating the spread of the pandemic.

In this finding, about 81.67% of the knowledge questions were correctly replied to by the respondents. This finding was in line with the study conducted in Saudi Arabia (80.5%) (4) and in Nigeria (77.36) (35). The finding of this study was lower than the study conducted in China (90%) (36). This discrepancy may be due to variation in the study population's characteristics, government commitment, and health care system quality on awareness creation. On the contrary, this study result was higher than in the Egyptian population (71.26%) (37). This discrepancy might be due to Spatio-temporal variation.

The finding of this study revealed that 69.3 % (CI; 65.1-73.8) of the participants had good knowledge of COVID-19 which was consistent with a finding in India (70 %) (38). On the other

hand, the finding of this study was lower than a multicenter study conducted among health care workers in Ethiopia with 88.2% (16) and Nigerian residents in an urban setting (99.7%) (39). This deviation may be due to the change in the study population (health care professionals vs. general population) and residents of the study population.

This finding showed that almost all (96%) of the respondents heard about COVID-19 by using different sources of information. About two-thirds (62.4%) of the participants used social media as a source of information of COVID-19 which was slightly higher than the study conducted in Nigeria 55% (39). This deviation may be due to a change in the study period and setting, socio-demographic characteristics of the study population. On the contrary, this finding was lower than the study conducted in Ethiopia (73.6%) (16). This deviation may be due to a change in the heterogeneity of the study population (general population vs. health care professionals), and variation of participants resident. Furthermore, this study also indicated that about 80% of participants knew that the elderly, those who had chronic medical illnesses, and obese are more likely to develop severe cases of COVID-19. This finding was slightly higher than the study conducted in Ethiopia (72.5%) (11). This variation may be due to the change in Spatio-temporal variation, socio-demographic characteristics of the study population, and coverage of awareness creation towards COVID-19. Even though children and young adults are vulnerable groups, only 83.4% of the participants knew that these groups need to take preventive measures towards COVID-19. Neglecting such types of the population may wide-spreading the transmission of the pandemic (11).

Regarding the attitudes, 62.6% (95% CI; 57.2-67.6) of respondents had a positive attitude towards COVID-19 which was lower than the study conducted in Ethiopia (94.7%) (16), Nigeria 79.5% (39), and Pakistan (82.16%) (40). This discrepancy may be due to a change in the study

population (health professionals vs general population), government commitment towards COVID-19. On the other hand, less than half (44.6%) of the participants believed that the government of Ethiopia can control the spread of COVID-19 within a short time. This finding was lower than the study conducted in china 97.1% (24) and India at 87.2% (41). This deviation may be due to the variation in the quality of the health system, socio-demographic characteristics of the study population, and government preparedness to respond to the control of the pandemic. The report of WHO showed that the government's of Ethiopia scored only 52% towards COVID-19 preparedness response (21) which supports the finding of this study. Furthermore, this study also indicated that almost two-thirds of the respondents believed that the pandemic of COVID-19 leads to the development of social stigma which was lower than a study conducted in Ethiopia at 77% (16) and 83.8% (11). This deviation may be due to differences in Spatio-temporal variation, socio-demographic characteristics of the study population. On the contrary, this finding was higher than the study conducted in the Peruvian population 59.1% (42). This variation may be due to a change in the socio-demographic characteristics of the study population and time, awareness creation towards COVID-19, and the burden of the pandemic. The social stigma may be developed due to fear of its mortality and high communicability. The history of social stigma due to pandemic was not a new phenomenon (43,44).

Regarding the prevention practice of COVID-19, the overall practice score of the respondents was 73.2% which was higher than the study conducted in Ethiopia (26). The finding of this study showed that only half 49.3% of the participants had a good preventive practice of COVID-19. But this finding was lower than other studies conducted in Ethiopia (16,26) and China (45). This variation may be due to the change in the study setting, socio-demographic characteristics of the study population, and occupation of the participant (being a health professional vs. general

Formatted: Justified

population). This finding also indicated that 93.6% of the respondents wash their hands with water and soap for at least 20 seconds which was consistent with the study conducted in Nigeria 96.4% (39). On the contrary, this finding was lower than a study conducted in Nigeria 87.9 % (35). This deviation may be to a variation in access and proper utilization of handwashing facilities in health care facilities. Additionally, 83.7% of the participants used face masks which were consistent with the study conducted in Nigeria 84.4% (35), and 82.3% (39). On the other hand, less than half (43.8%) of the respondents applied in Keeping physical distance when they go to public areas. This finding was lower than the study conducted in Nigeria 83% (35) and 92.7%, (39). This variation may be due to a difference in the study population, the burden of the disease, awareness of the community towards the pandemic, and population way of life. The finding also revealed that more than two-thirds of 70% of the respondents avoid going to crowded places after the emergence of COVID-19 which was higher than the finding in Nigeria 58.9% (35). Additionally, 82.4% of the respondents practiced respiratory hygiene which was lower than the finding in India with a value of (97.7%) (46) which may be due to the variation in a study setting, heterogeneity of population perception of the community, Knowledge towards COVID-19, and burden of confirmed COVID-19 cases. Above all, the most common challenge of COVOD-19 prevention practice was staying at home after the occurrence of COVID-19. Based on this finding, less than one-third (30%) of the respondents apply it. The possible justification for this problem may be the stay of the pandemic for a long period of time andmost Ethiopian population depends on subsitence way of life.

Conclusion

In conclusion, the finding of this study showed 70 % of the medical visitors had good knowledge while 62.4% had a positive attitude towards COVID1-19. But there was a problem in applying to practice what they know and perceive towards COVID-19. As a result, only half of the participants had good prevention practices of COVID-19. Seventy percent and above respondents had practiced most prevention measures of COVID-19 except for keeping of physical distance and staying at home. Therefore, the zonal health bureau should work in collaboration with other stakeholders to improve the awareness towards COVID-19 prevention practice mainly in public gathering areas like markets, schools, healthcare facilities, religious institutions, and bus stations. Furthermore, the Ethiopian Ministry of Health should link COVID-19 prevention with health extension programs mainly in rural parts of the country.

Abbreviations

COVID-19:Coronavirus disease 2019; 2019-novel coronavirus: 2019-novel coronavirus; AOR: Adjusted Odds Ratio; ARDS: Acute Respiratory Distress Syndrome; CDC: Communicable disease control; CI: Confidence Interval; COR: Crude Odds Ratio; COVID_19: Corona Virus Disease-2019; CSA: Central Statistical Agency; HCP: Health Care Professionals; HCWs: Health Care Workers; PHEIC: Public Health Emergency of International Concern; PPE: Personal Protective Equipment; LMC: Low and Middle-Income Countries; SARAS_COV-1: Severe Acute Respiratory Syndrome- Corona Virus-1; SARS-COV-2: Severe Acute Respiratory Syndrome- Corona Virus-2; SPSS: Statistical Package for Social Science; WHO: World Health Organization;

Declarations

Ethics approval and consent to participate

The study was approved by the ethical review committee of Debre Tabor University. Permission to conduct the study was obtained from the respective hospital managers of the study site. Before the data collection, the purpose of the study was explained and verbal consent was obtained from each participant. Individuals who were volunteer to participate in the study were also told as they have the right to withdraw from the study at any stage of the interview. The confidentiality of the study participants was ensured by avoiding possible identifiers. Data collectors wear a facemask and keep a physical distancing of two feet. Facemask was provided for the study participants who did not wear it during the data collection.

Consent for publication

Not applicable

Availability of data and material

Data and all the materials will be available from the corresponding author upon request.

Conflicts of interest

The authors declare that they have no conflicts of interest.

Funding

No extra funding for this study

Authors' contributions

Conceptualization: Gete Berihun**,** Zebader Walle Belete, Awoke Keleb, Ayechew Ademas

Data curation: Gete Berihun**,** Awoke Keleb, Ayechew Ademas

Formal analysis: Gete Berihun**,** Zebader Walle Belete, Awoke Keleb, Ayechew Ademas

Funding acquisition: Investigation: Gete Berihun**,** Zebader Walle Belete, Awoke Keleb, Ayechew Ademas

Methodology: Gete Berihun**,** Zebader Walle Belete, Awoke Keleb, Ayechew Ademas

Project administration: Gete Berihun**,** Zebader Walle Belete,

Resources: Gete Berihun**,** Zebader Walle Belete, Awoke Keleb, Ayechew Ademas

.**Software:** Gete Berihun**,** Zebader Walle Belete, Awoke Keleb, Ayechew Ademas

Supervision:. Gete Berihun**,** Zebader Walle Belete, Awoke Keleb, Ayechew Ademas

Validation: Gete Berihun**,** Zebader Walle Belete, Awoke Keleb, Ayechew Ademas

Visualization: Gete Berihun**,** Zebader Walle Belete, Awoke Keleb, Ayechew Ademas

Writing – original draft: Gete Berihun**,** Zebader Walle Belete, Awoke Keleb

Writing – review & editing: Gete Berihun**,** Zebader Walle Belete, Ayechew Ademas

Acknowledgments

The authors express their heartfelt gratitude to Debretabore University and all the participants of the study who gave valuable information and their precious time for this study. We are also grateful for data collectors, supervisors, and Health care facility administrative officials.

References

1. Elhadi M, Msherghi A, Alkeelani M, Zorgani A, Zaid A, Alsuyihili A, et al. Assessment of healthcare workers' levels of preparedness and awareness regarding Covid-19 infection in low-resource settings. Am J Trop Med Hyg. 2020;828–33.

2. WHO Regional Office for Africa. COVID-19 Situation Update for WHO African Region, External Situation Report. 2020 Jun. Report No.: 15.

3. Saqlain M, Munir MM, Ur Rehman S, Gulzar A, Naz S, Ahmed Z, et al. Knowledge, attitude, practice and perceived barriers among healthcare professionals regarding COVID-19: A Cross-sectional survey from Pakistan. Occupational and Environmental Health; 2020 Apr.

4. Al-Hanawi MK, Angawi K, Alshareef N, Qattan AMN, Helmy HZ, Abudawood Y, et al. Knowledge, Attitude and Practice Toward COVID-19 Among the Public in the Kingdom of Saudi Arabia: A Cross-Sectional Study. Front Public Heal. 2020;8(May).

5. Iannone P, Castellini G, Coclite D, Napoletano A, Fauci AJ, Iacorossi L, et al. The need for health policy perspective to protect Healthcare Workers during COVID-19 pandemic. A GRADE rapid review on the effectiveness of the N95 respirator. PLoS One [Internet]. 2020;15(6):1–13. Available from: http://dx.doi.org/10.1371/journal.pone.0234025

6. Nallani VRR, Nadendla RR, Kavuri NSS. Knowledge, attitude, and practice among health care professionals regarding COVID-19 and barriers faced by health care professionals inSouth India. Int J Community Med Public Heal. 2020;7(9):3450.

7. Lone SA, Ahmad A. COVID-19 pandemic – an African perspective. :10.

8. Baye K. COVID-19 prevention measures in Ethiopia Current realities and prospects. The Federal Democratic Republic of Ethiopia Policy Studies Institute; 2020.

9. Ssebuufu R, Katembo Sikakulya F, Binezero SM, Wasingya L, Nganza SK, Ibrahim B, et al. Awareness knowledge attitude and practice towards measures for prevention of the spread of COVID-19 in the Ugandans A nationwide online cross-sectional Survey. Medrxiv. 2020;1–28.

10. WHO. COVID-19 Strategic Preparedness and Response Plan Operational Planning Guideline to Support Country Preparedness and Response. 2020.

11. Kebede Y, Yitayih Y, Birhanu Z, Mekonen S, Ambelu A. Knowledge, perceptions and preventive practices towards COVID-19 early in the outbreak among Jimma university medical center visitors,Southwest Ethiopia. PLoS One [Internet]. 2020;15(5):1–15. Available from: http://dx.doi.org/10.1371/journal.pone.0233744

12. Worldometer Report COVID-19 coronavirus pandemic 2020. https://www.worldometers.info/coronavirus, 2020 accessed date October 6, 2020, 9.54 am

13. Worldometer report.COVID 19 coronavirus pandemic 2020. https://www.worldometers.info/coronavirus/country/ethiopia,2020 accessed date October 6, 2020, 9.54 am

14. Nigussie T.F and Azimach N.N. Knowledge, Attitude and Practice towards COVID-19 among Arba Minch Town GSJ : Volume 8, Issue 6, June 2020, Online : ISSN 2320-9186

15. CDC. Infection prevention and control for COVID-19 in healthcare settings - first update 12 March 2020. Eur Cent Dis Prev Control. 2020;(March):1–10.

16. Jemal B, Ferede ZA, Mola S, Hailu S, Abiy S, Wolde GD, et al. Knowledge, attitude, and practice of healthcare workers towards COVID-19 and its prevention in Ethiopia: a multicenter study. In Review; 2020 May.

17. Olum R, Chekwech G, Wekha G, Nassozi DR, Bongomin F. Coronavirus Disease-2019: Knowledge, Attitude, and Practices of Health Care Workers at Makerere University Teaching Hospitals, Uganda. Front Public Heal. 2020 Apr;8:181.

18. Lakshmi Priyadarsini S, Suresh M. Factors influencing the epidemiological characteristics of pandemic COVID 19: A TISM approach. Int J Healthc Manag. 2020;13(2):89–98.

19. Saqlain M, Munir MM, Rehman SU, ... Knowledge, attitude, practice and perceived barriers among healthcare professionals regarding COVID-19: A Cross-sectional survey from Pakistan. J … [Internet]. 2020; Available from https://www.ncbi.nlm.nih.gov/pmc/articles/PMC7211584/

20. Goshu D, Ketema M. Assessment of COVID-19 Effects and Response Measures in Ethiopia : Livelihoods and Welfare Implications Assessment of COVID-19 Effects and Response Measures in Ethiopia : Livelihoods and Welfare Implications 1. 2020.

21. Tolu LB, Ezeh A, Feyissa GT. How Prepared Is Africa for the COVID-19 Pandemic Response ? The Case of Ethiopia What Measures Should Be Taken to. 2020;

22. Alemayehu TY, Boled AT, Taye MG, Kibret S. Assessment of emergency and essential surgical service in primary hospitals ofSouth Gondar zone, Amhara region, Ethiopia, 2019 International Journal of Surgery Open Assessment of emergency and essential surgical

service in primary hospitals ofSouth Gondar zone, Amhara region, Ethiopia, 2019. Int J Surg Open [Internet]. 2020;23(February):35–8. Available from: https://doi.org/10.1016/j.ijso.2020.02.003

23. Abate BA, Ahunie MA. Basic Emergency Maternal And Neonatal Care Status OfSouth Gon- Dar Zone, North Central Ethiopia: Institutional Descriptive Sur- Vey, June 2016. 2017;9(1):11.

24. Giao H, Han NTN, Khanh T Van, Ngan VK. Knowledge and attitude toward COVID-19 among healthcare workers at District 2 Hospital, Ho Chi Minh City. Asian Pac J Trop Med. :7.

25. Defar A, Molla G, Abdella S, Tessema M, Ahmed M, Tadele A et. al. Knowledge, Practice and associated factors towards the Prevention of COVID-19 among high-risk groups: A cross-sectional study in Addis Ababa, Ethiopia. 2020;19.

26. Asemahagn MA. Factors determining the knowledge and prevention practice of healthcare workers towards COVID-19 in the Amhara region, Ethiopia : a cross-sectional survey. 2020;3.

27. Salman M, Mustafa ZU, Asif N, Zaidi HA, Shehzadi N. Knowledge, attitude and preventive practices related to COVID-19 among health professionals of Punjab province of Pakistan.

28. WHO E. Survey Tool And Guidance Rapid, simple, flexible behavioral insights on COVID-19. 2020.

29. Ahmad NA, Ahmad W, Arman R, Rahimi W, Ahmadi A, Vijay Raghavan SMS. Community Perception Survey – COVID 19 Knowledge, Attitude, and Practice Survey in Kabul, Kunduz, and Khost Province. the JOHANNITER international assistance, JACK, OHW; 2020.

30. National Comprehensive Covid19 Management Handbook. :162.

31. National Institute of Environmental Health Science. COVID-19_BSSR_Research_Tools.pdf. 2020.

32. Admissions R, Homes R. COVID-19 : Infection Prevention and Control Checklist for Long-Term Care and Retirement Homes. 2020;1–15.

33. WHO. COVID-19 Infection Prevention and Control Sameeksha. 2020;2.

34. Ahmed N, Shakoor M, Vohra F, Abduljabbar T, Mariam Q, Rehman MA. Knowledge, Awareness, and Practice of Health care Professionals amid SARS-CoV-2, Corona Virus Disease Outbreak. :8.

35. Okoro J, Ekeroku A, Nweze B, Odionye T, Nkire J, Onuoha M, et al. Attitude and preventive practices towards COVID-19 disease and the impact of awareness training on knowledge of the disease among correctional officers [version 1 ; peer review : awaiting peer review]. 2020;1–10.

36. Zhong B, Luo W, Li H, Zhang Q, Liu X, Li W, et al. Knowledge, attitudes, and practices towards COVID-19 among Chinese residents during the rapid rise period of the COVID-19 outbreak : a quick online cross-sectional survey. 2020;16.

37. Abdelhafiz AS, Mohammed Z, Ibrahim ME, Ziady HH, Alorabi M, Ayyad M, et al. Knowledge, Perceptions, and Attitude of Egyptians Towards the Novel Coronavirus Disease (COVID-19). J Community Health [Internet]. 2020;45(5):881–90. Available from: https://doi.org/10.1007/s10900-020-00827-7

38. Singh JP, Sewda A. Assessing the Knowledge, Attitude, and Practices of Students Regarding the COVID-19 Pandemic. 2020;

39. Christopher R, Margaret R, Dauda MAD, Saleh A, Ene P. Knowledge, Attitudes and Practices Towards COVID - 19 : An Epidemiological Survey in North - Central Nigeria. J Community Health [Internet]. 2020; (0123456789). Available from: https://doi.org/10.1007/s10900-020-00881-1

40. Hussain I, Majeed A, Imran I, Ullah M, Hashmi FK, Saeed H, et al. Knowledge, Attitude, and Practices Toward COVID - 19 in Primary Healthcare Providers : A Cross-Sectional Study from Three Tertiary Care Hospitals of Peshawar, Pakistan. J Community Health [Internet]. 2020;(0123456789). Available from: https://doi.org/10.1007/s10900-020-00879-9

41. S DP, B KR, Athul K, Swamy S, Bhodaji S, Deshmukh A, et al. Knowledge, Attitude, Awareness and Practice Towards Covid-19 Pandemic in Indian Citizens During The National Lockdown Period : A Quick Online Cross-. 2020;8(May):3504–15.

42. Zegarra-Valdivia, J. A. 1; Chino-Vilca, B. N. 1; & Ames-Guerrero R. Knowledge, perception and attitudes regarding COVID-19 Pandemic in Peruvian Population.

43. Person B, Sy F, Holton K, Govert B, Liang A, Sars N. Fear, and Stigma : The Epidemic within the SARS Outbreak. 2004;10(2).

44. Paget J. ITALIAN OF PUBLIC HEALTH The influenza pandemic and Europe : the social impact and public health response OF PUBLIC. 2009;6(3):257–9.

45. Zhou M, Tang F, Wang Y, Nie H, Zhang L, You G, et al. Knowledge, attitude and practice regarding COVID-19 among health care workers in Henan, China. J Hosp Infect [Internet]. 2020; Available from https://doi.org/10.1016/j.jhin.2020.04.012

46. Awasthi AA, Taneja N, Janardhanan R. Knowledge, Attitude And Practice Towards Covid-19 Pandemic Among Residents Of Bihar, India. 2020;11(D):38990–5.

Table 1: Socio-demographic characteristics of medical visitors in hospitals ofSouth Gondar zone Northwestern Ethiopia, in August 2020 (n=404)

Variable	Category	Frequency	Percentage
Sex	Male	163	40.3

	Female	241	59.7
Age(years)	<20	24	5.9
	20-29	92	22.8
	30-39	111	27.5
	40-49	94	23.3
	50-59	48	11.9
	≥60	35	8.7
Religion	Muslim	30	7.4
	Orthodox	331	81.9
	Protestant	43	10.6
Marital status	Single	83	20.5
	Married	295	73.1
	Divorced	26	6.5
Educational status	Cannot read and wright	66	16.3
	Read and write	95	23.5
	Primary (1-8) grade	36	8.9
	Secondary (9-12)grade	29	7.2
	College and above	178	44.1
Occupation	Farmer	68	16.8
	Student	45	11.1
	Unemployed	55	13.6
	Government employer	129	31.9
	Private business worker	107	26.5
Resident	Urban	287	71.0
	Rural	117	29.0
Monthly income(ETB)	≤499	127	31.4
	500-2000	101	25
	≥2001	176	43.6
History of chronic medical illness	Yes	56	13.9
	No	348	86.1
Training on COVID 19	Yes	137	33.9
	No	267	64.1
Use social media	Yes	252	62.4
	No	152	37.6
Peer as a source of information of COVID19	Yes	345	85.4
	No	59	14.6
Use TV/radio as a source of information	Yes	321	79.5
	No	83	20.5
Use religious institution as source of information	Yes	130	32.2
	No	274	67.8

Table 2: Knowledge of medical visitors towards COVID-19 in hospitals ofSouth Gondar zone, Northwestern Ethiopia, in August 2020 (n=404)

**Item**	Yes		No		I do not know	
	Frequency	%	Frequency	%	Frequency	**%**

Did you hear about COVID-19?	388	96.0	16	4.0		
COVID-19 is a viral disease.	322	79.7	40	9.9	42	10.4
The major sign and symptoms of COVID-19 are dry cough, fever, and shortness of breathing.	339	83.9	33	8.2	32	7.9
Runny nose and sneezing are less common symptoms of COVID-19.	275	68.1	88	21.8	41	10.1
Elder, those who have a chronic medical illness and obese are more likely to sever the case of COVID- 19.	320	79.2	57	14.1	27	6.7
Currently, there is no effective cure for COVID-19.	331	81.9	50	12.4	23	5.7
COVID-19 virus can spread via respiratory droplets.	375	92.8	29	7.2		
Eating and contacting wild animals would result COVID-19 infection	308	76.2	53	13.1	43	10.6
Persons with COVID 19 virus can transmit the virus to others when a fever is not present	283	70.0	71	17.6	50	12.4
Proper washing hand with soap and water is one method of preventing COVID-19.	375	92.8	18	4.5	11	2.7
Wearing general masks can prevent one from acquiring infection by the COVID 19 virus	354	87.6	35	8.7	15	3.7
Children and young adults must take measures to prevent the infection by Covid 19 virus	337	83.4	45	11.1	22	5.4
To prevent the infection by COVID 19 virus individuals should avoid going to crowded places such as bus parks and avoid public transportation	352	87.1	50	12.4	2	.5
People who have contact with someone infected with COVID 19 virus should be	273	67.6	95	23.5	36	8.9

immediately isolated in a proper place in general the observation period is 14 days						
Isolation and treatment of people who are infected with the COVID 19 virus are effective ways to reduce the spread of the virus	295	73.0	80	19.8	29	7.2
Mean± standard deviation **12.25±2.45**						
Minimum **2.00**						
Maximum **15**						

Table 3:-Attitude of medical visitors towards COVID-19 in hospitals ofSouth Gondar zone, Northwest Ethiopia, in August 2020 (n=404)

Item	Agree	Neutral	Disagree
Black races are not protected from COVID 19 disease.	203(50.2%)	146(36.1%)	55(13.6%)
Wearing a well-fitting face mask are effective in preventing COVID 19 virus	268(66.3%)	81(20.0%)	55(13.6%)
Hand wash can prevent you from COVID 19 virus	321 79.5	77 19.1	6 1.5
Ethiopia is in a good position to contain COVID 19 virus	180 44.6	144 35.6	80 19.8
COVID 19 is not stigma and I should not hide my infection	274 67.8	90 22.3	40 9.9
If I get infected with COVID 19, I will go to the hospital as advised.	221 54.7	141 34.9	42 10.4
I can get infected with COVID 19 if I contacted an infected patient despite my good immunity.	230 56.9	100 24.8	74 18.3
COVID 19 is fatal	215 53.2	105 26.0	84 20.8
During the outbreak of COVID 19 eating well cooked and safely handled meat is safe.	249 61.6	96 23.8	59 14.6
COVID 19 patients should share their recent travel history with a health care provider.	256 63.4	85 21.0	63 15.6
Do you think that the cause of Covid-19 is not spiritual/	262 64.9	87 21.5	55 13.6

is it happened because of our sin?			
Mean ±standard deviation **27.11±4.08**			
Minimum **17**			
Maximum **33**			

Table 4:-Preventive practice of medical visitors towards COVID-19 in hospitals ofSouth Gondar zone, Northwest Ethiopia, in August 2020 (n=404)

Item	Yes		No	
	Frequency	%	Frequency	%
Do you avoid handshaking to prevent covid 19?	363	89.9	41	10.1
Have you washed your hands often with soap and water for at least 20 seconds especially after you have been in a public place or after blowing your nose, coughing, or sneezing?	378	93.6	26	6.4
If soap and water are not readily available, are you applying a hand sanitizer that contains at least 60% alcohol?	309	76.5	95	23.5
Do you wear face masks repeatedly when you leave your home?	338	83.7	66	16.3
Do you coughing and sneezing into the elbow or within clothing?	333	82.4	71	17.6
In recent days have you avoid going to any crowded place?	281	69.6	123	30.4
Do you avoid eating raw animal products to prevent the COVID 19 virus?	336	83.2	68	16.8
Do you avoid touching your mouth nose and eyes with unwashed hands?	323	80.0	81	20.0
Do you keep your self 2m away from the others when you got to the public area?	177	43.8	227	56.2
Do you stay at your home after the emergent of covid 19?	121	30.0	283	70.0
Mean ±standard deviation **7.32±1.60**				

Minimum	**1.00**				
Maximum	**10**				

Table 5:-Predictors of knowledge towards COVID-19 among medical visitors in hospitals ofSouth Gondar zone, Northwest Ethiopia, August 2020 (n = 404).

Variable	Knowledge		COR(95% CI)	AOR(95% CI)	P-value
	Poor	Good			
Age					
<20	12	12	1	1	
20-29	22	70	3.182(1.252-8.085)	1.98(0.54-7.29)	0.31
30-39	29	82	2.828(1.144-6.992)	1.08(0.30-3.87)	.091
40-49	39	58	1.611(.654-3.970)	1.06(0.31-3.68)	0.92
50-59	15	33	2.200(.804-6.018)	1.21(0.30-4.82)	0.79
≥60	10	25	2.500(.844-7.401)	0.69(0.16-2.95)	0.61
Marital status					
Single	22	61	1	1	
Married	90	205	0.821(0.48-1.42)	0.88(0.42-1.82)	0.72
Divorced	12	14	0.42(0.17-1.05)	0.92(0.29-2.98)	0.89
**Education**					
cannot read and write	41	25	1	1	
Read and write	41	54	2.16(1.14-4.12)	**2.78(1.18-6.56)***	**0.02***
Primary	10	26	4.26(1.76-10.31)	2.42(0.56-10.44)	0.24
Secondary	8	21	4.31(1.66-11.18)	1.54(0.25-9.56)	0.65
College and above	24	154	10.52(10.52-5.45)	**6.15(2.18-17.40)***	**0.001***
Occupation					
Farmer	45	23	1	1	
Student	12	33	5.38(2.35-12.34)	1.64(0.28-9.72)	0.59
Currently unemployed	17	38	4.37(2.04-9.36)	1.50(0.49-4.58)	0.48
Gov’t worker	18	111	12.07(5.95-24.48)	0.83(0.16-4.19)	0.82

Private business	32	75	4.59(2.39-8.80)	0.91(0.25-3.30)	0.89
Resident					
Urban	71	216	2.52(1.60-3.96)	1.427(0.75-2.71)	0.28
Rural	53	64	1	1	
Monthly income					
<499	54	73	1	1	
500-2000	34	67	1.46(0.85-2.51)	1.272(0.52-3.09)	0.60
>2000	36	140	2.88(1.73-4.78)	1.291(0.46-3.60)	0.63
Training					
Yes	24	113	2.82(1.70-4.67)	1.74(0.89-3.42)	0.11
No	100	167	1	1	
Use social media					
Yes	48	204	4.25(2.72-6.65)	**2.96(1.46-6.01)***	**0.003***
No	76	76	1	1	
Peer					
Yes	93	252	3.00(1.71-5.27)	1.09(0.48-2.51)	0.84
No	31	28	1	1	
TV/radio					
Yes	78	243	3.87(2.34-6.40)	1.07(0.43-2.65)	0.88
No	46	37	1	1	
Religious institution					
Yes	32	98	1.55(0.97-2.48)	0.93(0.50-1.73)	0.83
No	92	182	1	1	

~~Predictors of Attitude towards COVID-19~~

Table 6:-~~Predictors~~ of attitude towards COVID-19 among medical visitors in hospitals ofSouth Gondar zone, Northwest Ethiopia, August 2020 (n = 404).

Variable	Attitude		COR(CI)	AOR(CI)	P-value
	Negative	Positive			
Age					
<20	12	12	1	1	
20-29	35	57	1.63(0.66-4.02)	0.42(0.12-1.46)	0.17
30-39	45	66	1.47(0.61-3.56)	0.51(0.14-1.82)	0.30
40-49	37	57	1.54(0.63-3.79)	0.94(0.28-3.21)	0.93
50-59	16	32	2.00(0.74-5.44)	1.10(0.28-4.27)	0.89
≥60	6	29	4.83(1.47-15.87)	1.65(0.33-8.42)	0.55
Religion					
Muslim	5	25	3.27(1.05-10.20)	2.18(0.50-9.58)	0.30
Orthodox	129	202	1.02(0.53-1.96)	1.49(0.64-3.48)	0.36
Protestant	17	26	1	1	
**Education**					
cannot read and write	44	22	1	1	
Read and write	48	47	1.96(1.02-3.76)	2.39(0.99-5.79)	.053
Primary	9	27	6.00(2.41-14.93)	**6.49(1.52-27.78)***	**0.012***
secondary	8	21	5.25(2.01-13.74)	2.32(0.39-13.74)	.35
College and above	42	136	6.48(3.49-12.01)	**6.91(2.58-14.50)***	**0.0001***
Occupation					
Farmer	46	22	1	1	
Student	9	36	8.36(3.44-20.36)	1.87(0.33-10.72)	0.48
Currently unemployed	22	33	3.14(1.50-6.58)	.54(0.18-1.68)	0.29
Gov't worker	24	105	9.15(4.66-17.96)	.61(0.12-3.05)	0.55
Private business	50	57	2.38(1.26-4.50)	.29(0.07-1.12)	0.07
Resident					
Urban	94	193	1.95(1.26-3.02)	1.23(0.66-2.23)	0.51
Rural	57	60	1	1	
Monthly income					
<499	55	72	1	1	
500-2000	40	61	1.17(0.69-1.98)	.893(0.33-2.38)	0.82
>2000	56	120	1.64(1.02-2.63)	.569(0.19-1.70)	0.31
History of chronic illness					
Yes	8	48	4.19(1.92-9.12)	**5.00(1.71-14.67)***	**0.003***
No	143	205	1	1	
Training					
Yes	24	113	4.27(2.59-7.05)	**3.9(1.96-7.70)***	**0.0001***
No	127	140	1	1	
Use social					

media					
Yes	73	179	2.59(1.70-3.93)	1.20(0.59-2.44)	0.63
No	78	74	1	1	
Peer					
Yes	114	231	3.41(1.92-60.5)	**2.45(1.06-5.63)***	**0.04***
No	37	22	1	1	
Tv/radio					
Yes	100	221	3.52(2.13-5.81)	2.091(0.85-5.16)	0.11
No	51	32	1	1	
Religious institution					
Yes	30	100	2.64(1.64-4.23)	1.725(0.93-3.21)	0.09
No	121	153	1	1	

Predictors of preventive practice towards COVID 19

Table 7: Predictors of preventive practice towards COVID-19 among medical visitors in hospitals ofSouth Gondar zone, Northwest Ethiopia, August 2020 (n = 404).

Variable	poor practice	good practice	COR(CI)	AOR(CI)	P-value
**Age**					
<20	15	9	1	1	
20-29	44	48	1.82(0.72-4.57)	.87(0.24-3.38)	0.84
30-39	62	49	1.32(0.53-3.26)	.43(0.12-1.67)	0.22
40-49	53	41	1.29(0.51-3.24)	.75(0.20-2.56)	0.67
50-59	21	27	2.14(0.79-5.85)	.99(0.23-4.24)	0.98
≥60	10	25	4.17(1.38-12.58)	1.31(0.28-6.11)	0.73
**Education**					
cannot read and write	49	17	1	1	
Read and write	70	25	1.03(0.50-2.11)	.93(0.36-2.43)	0.88
primary	20	16	2.31(0.98-5.44)	.39(0.08-1.80)	0.23
secondary	12	17	4.08(1.62-10.27)	.82(0.16-4.19)	0.82
College and above	54	124	6.62(3.50-12.52)	1.90(0.67-5.17)	0.21
**Occupation**					
Farmer	62	6	1	1	
Student	21	24	11.81(4.25-32.83)	**7.70(1.15-15.86)***	**0.04***
Currently unemployed	35	20	5.91(2.17-16.08)	2.35(0.58-9.57)	0.23
Gov't worker	36	93	26.70(10.62-67.12)	2.49(0.42-14.61)	0.31
Private business	51	56	11.35(4.52-28.47)	2.15(0.45-10.2)	0.34

**Resident**					
Urban	121	166	3.49(2.19-5.56)	1.54(0.79-3.00)	0.21
Rural	84	33	1	1	
**Monthly income**					
<499	88	39	1	1	
500-2000	51	50	2.21(1.29-3.81)	2.05(0.71-5.93)	0.19
>2000	66	110	3.76(2.32-6.12)	1.99(0.62-6.39)	0.25
**Training**					
Yes	51	86	2.30(1.51-3.51)	0.88(0.47-1.64)	0.68
No	154	113	1	1	
**Use social media**					
Yes	92	160	5.04(3.23-7.87)	1.54(0.76-3.10)	0.23
No	113	39	1	1	
**Peer**					
Yes	161	184	3.35(1.80-6.25)	0.78(0.31-1.97)	0.61
No	44	15	1	1	
**Tv/radio**					
Yes	136	185	6.70(3.62-12.41)	1.45(0.53-3.96)	0.46
No	69	14	1	1	
**Knowledge**					
Poor knowledge	102	22	1	1	
Good knowledge	103	177	7.97(4.73-13.41)	**4.49(2.41-8.39)***	**0.0001***
**attitude**					
Negative attitude	107	44	1	1	
Positive attitude	98	155	3.85(2.50-5.93)	1.04(0.58-1.86)	.068

 Click here to access/download;Figure;Fig1.docx

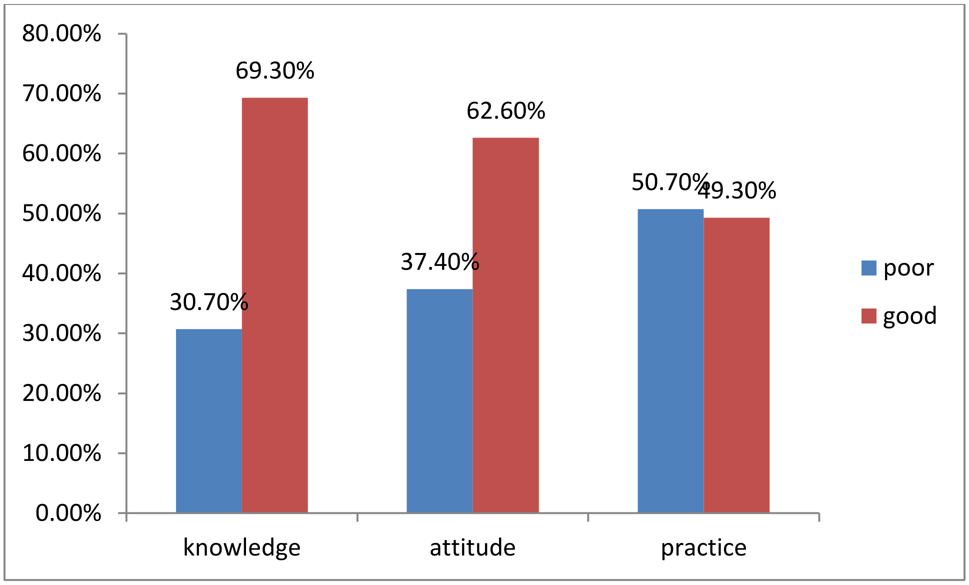

Fig1:-Status of Knowledge Attitude and Practice among Medical visitors in hospitals of South Gondar Zone, Northwest Ethiopia, in August 2020

Click here to access/download
Supporting Information
patient final manusript - submitted.docx
