# Supplementary material for: Knowledge, attitude, and preventive practices towards COVID-19 and associated factors among adult hospital visitors in South Gondar Zone Hospitals, Northwest Ethiopia
Source: PLoS One. 2021 May 17;16(5):e0250145. doi: 10.1371/journal.pone.0250145 (PMC8128268; doi:10.1371/journal.pone.0250145)
Supplement: Attachment — Submitted filename: Comment.pdf [file pone.0250145.s008.pdf]

PLOS ONE

Knowledge, Attitude, and Practices towards COVID-19 and associated factors among adult hospital Visitors in South Gondar Zone Hospitals, Northwest Ethiopia

--Manuscript Draft--

**Manuscript Number:**	PONE-D-20-36453R1
**Article Type:**	Research Article
**Full Title:**	Knowledge, Attitude, and Practices towards COVID-19 and associated factors among adult hospital Visitors in South Gondar Zone Hospitals, Northwest Ethiopia
**Short Title:**	Knowledge, Attitude, and Practices towards COVID-19 and associated factors among adult hospital Visitors
**Corresponding Author:**	Gete Gelaw Berihun, MSc<br>Wollo University<br>Dessie, ETHIOPIA
**Keywords:**	Knowledge, attitude, practice, associated factors, COVID-19, Ethiopia
**Abstract:**	Background<br><br>Coronavirus disease 2019 is currently the critical health problem of the globe, including Ethiopia. Visitors of healthcare facilities are the high-risk groups due to the presence of suspected and confirmed cases of coronavirus 2019 in healthcare setting. Increasing the knowledge, attitude and practices towards COVID-19 prevention among hospitals visitors are very important to prevent transmissions of the pandemic despite lack of evidence remains a challenge in Ethiopia. Therefore, this study was designed to investigate the status of knowledge, attitude, and practice towards COVID-19 and associated factors among hospital visitors in South Gondar Zone Hospitals, Northwest Ethiopia.<br>Methods<br><br>A facility-based cross-sectional study design was employed during August 1 to 30, 2020 from randomly selected 404 hospital visitors in South Gondar Zone Hospitals, northwest Ethiopia. Data was collected using interviewer administered questionnaire. The outcome of this study was good or poor knowledge, positive or negative attitude and good or poor practice towards COVID-19. A binary logistic regression model with 95% CI (Confidence interval) was used for data analysis. Bivariable analysis with (COR [crude odds ratio]) and multivariable analysis (AOR [adjusted odds ratio]) was used during data analysis. From the bivariable analysis, variables with a p- value < 0.25 were retained into the multivariable logistic regression analysis. From the multivariable logistic regression analysis, variables with a significance level of p -value < 0.05 were taken as factors independently associated with knowledge, attitude and practices towards COVID-19.<br>Main findings<br>About 69.3% of the respondents had good knowledge, 62.6% had positive attitude, and 49.3% had good practice towards the prevention of COVID-19. We found that factors significantly associated with good knowledge about COVID-19 were educational status who can read and write (AOR=2.78; 95%CI: 1.18, 6.56) and college and above (AOR=6.15; 95%CI: 2.18-17.40), and use of social media (AOR=2.96; 95%CI: 1.46, 6.01). Furthermore, factors significantly associated with positive attitude towards COVID-19 includes presence of chronic illnesses (AOR=5.00; 95%CI; 1.71-14.67), training on COVID-19 (AOR=3.91; 95%CI: 1.96-7.70), and peer/family as a source of information (AOR=2.45; 95%CI: 1.06-5.63). Being a student (AOR=7.70; 95%CI: 1.15-15.86) and participants who had a good knowledge on COVID-19 (AOR=4.49; 95%CI: 2.41-8.39) were factors significantly associated with good practice towards COVID-19.<br>Conclusion<br>We conclude that the status of knowledge, attitude and practice towards COVID-19 prevention was not satisfactory. Factors significantly associated with good knowledge were educational status who can read and write and college and above and use of social media. Factors significantly associated with positive attitude include presence of chronic illnesses, training on COVID-19, and peer/family as a source of information.

Powered by Editorial Manager® and ProduXion Manager® from Aries Systems Corporation

	Being a student and participants who had a good knowledge were factors significantly associated with good practice towards COVID-19. Hence, intervention strategies such as health education and infection prevention and control that could improve the knowledge, attitude and practice status towards COVID-19 preventions are urgently needed to control the transmission of COVID-19.
**Order of Authors:**	Gete Berihun, MSc
	Zebader Walle Belete
	Awoke Keleb
	Ayechew Ademas
	Leykun Berhanu
	Masresha Abebe
	Adinew Gizeyatu
	Seada Hassen
	Daniel Teshome
	Mistir Lingerew
	Alelgne Feleke
	Tarikuwa Natnael
	Metadel Adane
**Response to Reviewers:**	Date: March 12 2021<br>Manuscript ID: PONE-D-20-36453R1<br>Preventive Practice and Associated Factors towards COVID-19 among Medical Visitors in Hospitals of South Gondar Zone, Northwest Ethiopia<br>Corresponding authors: Gete Berihun (MSc)<br><br>Dear Dr, Francesco Di Gennaro (PhD)<br>Academic Editor<br>PLOS ONE<br>Thank you for your letter dated January 27, 2021 with a decision of major revision needed. We were pleased to know that our manuscript was considered potentially acceptable for publication in PLoS ONE, subject to adequate revision as requested by the reviewers, academic editors and the journals. Based on the instructions provided in your letter, we uploaded the file of the rebuttal letter; the marked up copy of the revised manuscript highlighting the changes made in the original submitted version and the clean copy of the revised manuscript.<br>We have revised the manuscript by modifying the abstract, introduction, methods, results, discussion and other sections, based on the comments made by the reviewers and using the journal guidelines. Therefore, we have marked in red color all the changes made during the revision process. Appended to this letter is our point-by-point response (rebuttal letter) to the comments made by the reviewers.<br>We have agreed with almost all the comments and questions raised by the reviewers and academic editors. We also provided justification for those comments and questions for disagreeing. We would like to take this opportunity to express our thanks to the reviewers for their valuable comments and to thank you for allowing us to resubmit a revision of the manuscript.<br>I hope that the revised manuscript is accepted for publication in PLoS ONE.<br>Sincerely yours,<br>Gete Berihun (Wollo University)<br>Response to the Journal Requirements Questions<br>Response to editor<br>Question #1 Please ensure that your manuscript meets PLOS ONE's style requirements, including those for file naming.<br>Response: Thank you for this remark. We re-formatted the revised manuscript using the PLoS ONE format guidelines. The whole content of the manuscript, including the abstract, introduction, methods, discussion and reference are formatted using the guidelines (Please see the revised version for each section).

Powered by Editorial Manager® and ProduXion Manager® from Aries Systems Corporation

	Question #2. Data availability<br>Response. We have attached the data on the supplementary information<br>Question #3. Please amend either the title on the online submission form (via Edit Submission) or the title in the manuscript so that they are identical.<br>Response: Thank you for your comment. We made the title identical on the online submission and the title in the manuscript.<br>Question #4. Please amend either the abstract on the online submission form (via Edit Submission) or the abstract in the manuscript so that they are identical.<br>Response: Thank you for your comment and we did it accordingly.<br>Question #5. Your ethics statement should only appear in the Methods section of your manuscript. If your ethics statement is written in any section besides the Methods, please move it to the Methods section and delete it from any other section. Please ensure that your ethics statement is included in your manuscript, as the ethics statement entered into the online submission form will not be published alongside your manuscript.<br>Response: Thank you. We did it.<br>Question #6. We note that you have included a table to which you do not refer in the text of your manuscript. Please ensure that you refer to Table 2 in your text; if accepted, production will need this reference to link the reader to the Table.<br>Response: We have accepted the comments and hence the text written in Table 1 is changed in to Table 2.<br>Response to reviewer 1<br>Question #1. What is the difference between patient and visitors in your study?<br>Response: Sorry for the confusion about the two terms. We understand that we used the two terms interchangeably and now we updated the manuscript by hospital visitors. the patient is all persons who came to health care facilities for medical treatment. But visitors are any persons who came to the health care facilities for different purposes including seeking of medical treatment. Therefore, to avoid confusion we used visitors since the study was done on visitors, not only to patients (see the revised version).<br>Question #2. The document has not page number please incorporate<br>Response: Based on your comment, we gave the page number accordingly. Thank you.<br>Question # 3. Some sentences are incomplete which need intensive editing.<br>Response: We tried to assess errors like incomplete sentence, grammatical and language error from title up to discussion of the manuscript. As a result, the amendment was done accordingly in the revised version of the manuscript.<br>Questions #4. In the abstract section please add space between 'of' and 'south' as 'To assess COVID-19 preventive practice and associated factors among visitors in 30 hospitals of South Gondar Zone, Northwest Ethiopia.<br>Response: Thank you for your comment. We made a correction in the revised manuscript.<br>Question #5. In abstract section, "The questionnaire was pre-tested in 5% of the final sample size to establish the validity of the data collection instrument. The data were collected using face-to-face interviews by considering physical distancing and wearing of face masks. The data was entered in Epi-data version 3.1 and exported to Statistical Package for Social Science (SPSS) Version 25 for analysis." It is better delete and replace by tool of outcome variable measurement.<br>Response: Based on your comment we removed less important points from the abstract and amendment was done accordingly (see the revised new version of the manuscript).<br>Question #6. In abstract section, Bi-variate Crude Odd Ration (COR) with 95% confidence intervals (CI) and p-values of less than 0.25 were applied to select candidate variables for multi-variable analysis. Then, multi-variable Adjusted Odd Ratio (AOR) using binary logistic regression analysis at a p-value of less than 0.05 at 95% CI was shall be rephrased.<br>Response: Thank you for this key comment, we revised accordingly and please see the data analysis in page 9 and 10.<br>Question #7. In the abstract section, (AOR=2.96; 95% CI: 1.46, 6.01) were significantly associated with knowledge of COVID-1.' This is incomplete and does not give sense. It is better rewrite it again.<br>Response: We have accepted your comment hence this sentence rewritten as …were associated significantly with visitors' knowledge towards the prevention of COVID-19 (Please see the revised version of the abstract).<br>Question #8. In abstract section, the conclusion did not in line the finding. Please

Powered by Editorial Manager® and ProduXion Manager® from Aries Systems Corporation

conclude according to the finding.
Response: We have accepted your comment. The conclusion was amended based on the finding of the study (see the revised version of the manuscript).
Question #9. In Methods and Materials section, the patient flow data were estimated by reviewing the patients' logbook in the last three months and the average number of the patient for a month was calculated to determine the interval. Then, we used a systematic random sampling technique to select study participants of the study'. This paragraph is not clear. How to reach to apply systematic random sampling technique? What is the sampling frame? Is your study population are patients or patients attendance or any visitor of the hospital?

Response: We found that our way of writing was confusing. Sorry for the mistakes. After selecting the two hospitals randomly out of the 8 hospitals, we proportionally allocated sample size based on total estimated visitors of hospitals in the last three months. Then, 303 sample size was allocated for Debre Tabor general hospitals and 117 for Mekane Eyesus hospitals. Then hospitals visitors flow data during the previous 3 months in emergency ward, surgical ward, medical ward, gynecology/obstetrics ward and pediatrics ward considered for sample size allocation for each hospitals departments. Finally, randomly selection of visitors for each ward was selected until the allocated sample size was achieved (See the revised version in page 7.
Question #10. Under outcome and explanatory variables: please delete sensitive words like poor knowledge, attitude, and practice. It shall be replaced with 'favorable/unfavorable'
Response: We accepted your reflections, however, most studies used good/poor knowledge, positive and negative attitude and good/poor practice. We used these terms accordingly throughout the paper.
Question #11. Delete subtopic of 'operational definition.' This is already state in the outcome variables. Please avoid bolding words like 'good knowledge, poor knowledge, positive attitude, and Practice
Response: Thank you for your comment; we deleted subtopic of operational definition
Question#12. A pre-test was conducted using 5% (21) of the final sample size in the Andabet district to establish the validity of the questionnaire and amendment was made accordingly.' What type of amendment you made? Can you explain that amendment?
Response: The pre-test is aimed for amendment of measuring tool. Therefore, some of the amendments were arrangement, editing of unclear questions, and avoiding irrelevant questions.
Question 13. Under Statistical analysis: what is the different between bi- variate and multi-variable? What do you mean 'multi-variable'?
Response: Bivariate analysis refers one independent variable with outcome variables. However, multivariable means that more than one independent variables with the outcome variable. From the adjusted analysis, all variables that has a p-value less than 0.25 were included into the adjusted multivariable analysis to control confounders. In our study, in the case of this study we used bivariable and multivariable analysis. Furthermore, the word bivariate analysis was changed to bivariable analysis throughout the manuscript.
Question#14. Result section, use one of result presentation. Almost all tables are explained in the text. Please follow rule of text and table presentation together.
Response: Based on your comments we reduced more than half of the explanation. As a result, only pertinent finding of the study was explained (Please see all result section).
Question #15. In result section, similarly, 283 (70%) of the respondents knew that COVID-19 can be transmitted from one person to another even in the absence of COVID-19 (Table 1).' Table 1 presents socio-demographic characteristics of the participants but not knowledge of participants. Please cite the table appropriately.
Response: We made a correction accordingly (See the revised version of the manuscript).
Question #16. The finding of the study revealed seven out of ten 280 (69.3%) respondents had good knowledge towards COVID-19 while 253 (62.6%) had a positive attitude towards COVID-19. The finding of the study showed the pillar of prevention practice was much lower and only half 199(49.3%) of the participants had a score of good prevention practice of COVID-19 (Figure 1).' This paragraph is not clear.
Response: This idea is rewritten as'...280 (69.3% of study participants had favorable knowledge towards COVID-19 prevention. '... almost half of the study participants 119

(49.3%) are practiced the recommended COVID-19 prevention methods.'
Question #17. In result section, the finding revealed that those who can read and write were 2.78 times more likely to have good knowledge than those who can't read and write is not clear and should be re-write.
Response: We have rewritten as "The finding revealed that those who can read and write were 2.78 times more likely to have good knowledge towards COVID-19 prevention methods than those who can't read and write".
Question #18. In discussion section, this discrepancy 274 might be due to Spatio-temporal variation.' This is not a justifiable reason to the knowledge people towards novel coronal virus discrepancy of between Ethiopia and Egyptian population. Please search another justification of this discrepancy.
Response: Based on comment we tried to elaborate better justification for variation in this study with the finding of other researches conducted in different parts of the world.
Question #19. In discussion section, this deviation may be due to the change in the study population (health care professionals vs. general population) and residents of the study population. This makes confuse reader please rephrase again
Response: This idea is rewritten as" the deviation may be due to the difference in the study subjects. In the present study, the study subjects were visitors of Hospitals while the study conducted in Egypt were only health care professionals.
Response to reviewer 2
Question #1. The outcome variable and the title is not congruent. If your outcome variables are knowledge, attitude and practice towards COVID -19 prevention, your title should be modified to KAP of visitors towards COVID-19 prevention. Otherwise, if your title is focused only practice and associated factor, you have to include knowledge and attitude as part of associated factor, rather than the outcome variable.
Response: Based on the comment, we tried to make the title in line with its outcome variable. Therefore the title is modified in to KAP of visitors towards COVID-19 prevention while the outcome variables of the study are Knowledge, attitude, and practice towards COVID-19 prevention
Response to reviewer 3
Question #1. Result and discussion part 1. Please use software modeling for clear elaboration the topic of CVID 19 Examples OLS Model, add other better software
Response: We have already used logistic regression analysis using crude odds ratio (COR) and adjusted odds ratio (AOR) for determining the associated factors with the outcome variables. Associations between independent variables and knowledge, attitudes and practices towards COVID-19 were determined using a binary logistic regression model at 95% CI (Confidence interval). We used three different logistic regression models: The first model (Model 1) identified factors associated with good knowledge about COVID-19, the second model (Model II) identified factors associated with favorable attitudes and the third model (Model III) identified factors associated factors with good preventive practices towards COVID-19. For each model, bivariable analysis with (COR [crude odds ratio]) and multivariable analysis (AOR [adjusted odds ratio]) was used.
From the bivariable analysis, variables with a p-value <0.25 were retained into the multivariable logistic regression analysis. From the multivariable analysis of each model, variables with a significance level of p-value <0.05 were taken as factors independently associated with knowledge, attitude and practices towards COVID-19. The presence of multicollinearity among independent variables was checked using standard error at the cutoff value of 2 and we found that a maximum standard error of 0.97, which indicated no multi-collinearity. Model fitness was checked using the Hosmer-Lemeshow test for Model I, Model II and Model III and found a p-value of 0.650, 0.871 and 0.913, respectively and indicated that all models were fit.
We hope that the data analysis we used above is very sufficient to our study, which we could able to explain the result and discussion as we did it.
Question #2. Adding Images as possible
Response: We have 7 Tables and including more Figure is repeating of the result of the Table in another forms.
Question #3. Edit grammatical error and other
Response: This comment was also raised by other reviewers. We tried to revise of grammar, language, and punctuation errors starting from the title of the manuscript up to discussion (see the revised version of the manuscript). We appreciate your comment.
Response to reviewer 4
Question #1. What does medical visitor mean? is that for only visiting the medical

Powered by Editorial Manager® and ProduXion Manager® from Aries Systems Corporation

ward? if not, it is better to say among visitors.
Response: The study subjects all visitors of the health care facilities. So that, the title is modified to Knowledge, Attitude, and Practices towards COVID-19 and associated factors among hospital Visitors in South Gondar Zone Hospitals, Northwest Ethiopia

\Question #2. In the background section, please also include information on preventive practices of COVID-19.
Response: The title of the revised is modified to Knowledge, Attitude, and Practices towards COVID-19 and associated factors among hospital Visitors in South Gondar Zone Hospitals, Northwest Ethiopia. Therefore the emphasis is given not only for prevention measures but also for knowledge and attitude towards prevention of COVID-19. But in the revised manuscript, we elaborate detail COVID-19 prevention measures.
Question #3. Replace the word "face-to-face administered" by "interviewer administered"
Response: The phrase “face-to-face administered” is replaced by “interviewer administered”. (See the revised version of the manuscript).
Question #4. Please include the confidence intervals for these percentages with 95% CI.
Response: Knowledge, attitude, and practice of respondents are presented in the result and discussion section of the revised manuscript. According to this study, 69.3% (95%CI;65.1-73.8%) had a favorable knowledge, 62.6% (95%CI;57.2-67.6) had a favorable attitude, and 49.3%(95%CI;) had a favorable practice towards the prevention of COVID-19.

Question #5. If you did the associations for knowledge, attitude and practice of visitors towards COVID- 19 prevention, please rewrite your title as knowledge, attitude and practice of hospital visitors towards COVID-19 prevention....
Response: We have accepted your comment and the title was modified accordingly.
Question #6. Bivariate analysis refers two paired data sets/with two outcome variables. But your study has single outcome so, better to use bivariable.
Response: The word bivariate analysis was replaced by bivariable analysis throughout the revised version of the manuscript.
Question #7. Where does your pretest conducted and what are the psychomotor properties of that pretest? Particularly state clearly in your methods section, than the abstract.
Response: the pretest was conducted in other districts hospital visitors (andabet hospital) other than the study area. The psychomotor domains of the pretest are reliability and validity.
Question #8. How the response rate becomes 95.7%, if you use interviewer administered questionnaire?
Response: As we know the response rate of interviewer administered questionnaire is higher than self-administered once. But in the case of our study, the study subjects were visitors of health care facilities who came for different activities including medical treatments. The non-response rate of the study participants means that those study participants who are unable to provide data due to shortage of time, unwillingness to be part of the study and etc.

Question #9. Most paragraphs emphasized what COVID -19 entails and may be considered as too lengthy. Introduction should follow a structured and sequential order while capturing relevant information to be included in this section. What are the current issues about COVID -19 prevention? Some of these points were included in one of your paragraphs. What are the quantifiable effect/issue around the points identified on COVID- 19 prevention? Based on the earlier identified issues about COVID-19 prevention, what are the points to be addressed in your study? What is the rationale for the study? and lastly what is the aim of the study?
Response: We thank you for this key comment. Based on your comment we tried to shorten the introduction part. Furthermore we point out the current issues of COVID-19 prevention measures, earlier identified COVID-19 prevention measures, points to be addressed, rational of the study and finally aims of the study are explained in the revised version of the manuscript (See the updated manuscript).
Question #10. If you exclude individuals whose age is <18 years , it is better to modify your title as ... among adult visitors... otherwise, why you exclude these groups?
Response: Based on your comment we modified the title as to only adult visitors.

Question #11. You stated the total population of South Gondar Zone. However, it is better to state the average number of monthly visitors to hospitals within the zone because you are aiming to study visitors...
Response: we tried to modify this point method section particularly in study design, setting, and period of the revised version of the manuscript.
Question #12. If these are your outcome variables, your title should be revised as KAP (knowledge, attitude, and practice of visitors towards COVID-19 prevention...) otherwise use practice as your outcome variable and use knowledge, attitude, socio-demographic.... as your independent variables. Doing research without knowing the variables is meaningless.
Response: The title of the manuscript are modified in to KAP of adult visitors towards prevention of COVID- 19 to make in line with the outcome variable of favorable/ unfavorable knowledge, attitude, and practice towards prevention of COVID-19.
Question #13. Sampling procedure is not clear
Response: Sorry for the confusion. We rewrite the sampling procedure in a more clear manner. After selecting the two hospitals randomly out of the 8 hospitals, we proportionally allocated sample size based on total estimated visitors of hospitals in the last three months. Then, 303 sample size was allocated for Debre Tabor general hospitals and 117 for Mekane Eyesus hospitals. Then hospitals visitors flow data during the previous 3 months in emergency ward, surgical ward, medical ward, gynecology/obstetrics ward and pediatrics ward considered for sample size allocation for each hospitals departments. Finally, randomly selection of visitors for each ward was selected until the allocated sample size achieved.
Question #14. Attitude measurement is not clear. What does 26.4 (80%) score mean? is that the mean or median score of the overall attitude score?
Response: The attitude of the participants was measured using 11 items based three measurement scales with agree (3 points), neutral (2 points), and disagree (1 point). As a result the score varied from 11 to 33. Therefore, respondents with a mean score of ≥27 (80%) were considered as having a favorable attitude towards the prevention of COVID-19.
Question #15. Conducting of pre-test and keeping of the recommended physical distances should be considered as parts of ethical consideration rather than data collection.
Response: Based on the comment we moved the statement of keeping recommended physical distance during data collection to ethical consideration from method section (Please see the ethical consideration section).
Question #16. Is it feasible to conduct double data entery?
Response: We have written in a different term what we did, which is wrong. Thank you for your commitment in brining such errors for correction. We mean that and what we did was data entry was re-checked for 10% of the sample size in order to control data entry errors of the entered data and data cleaning was carried before statistical analysis. Thank you so much.

Question#17. The overall knowledge of the respondents should also be stated in figures, percentages using 95% CI based on the operational definitions stated in the methods section.

Response: Based on the operational definition, the knowledge of the respondents’ was presented using figures and percentages with 95%CI. Therefore, about 280 (69.3%) (CI; 65.1-73.8) of the participants had a good knowledge towards prevention of COVID-19.

Question #18. Your sample size is 422. However, you collect from 404 participants alone. Why? None response rate is rare in interviewer administered/ face to face questionnaire. Why this discrepancy arises?
Response: The response rate of the study was 95.7%. The response rate of interviewer administered questionnaire is higher than self-administered once. But in the case of our study, the study subjects were visitors of health care facilities who came for different activities including medical treatments. The non-response rate of the study participants means that those study participants who are unable to provide data due to shortage of time, unwillingness to be part of the study and etc. Since the study subjects were all visitors of the healthcare facilities who came for different activities including medical treatment. Therefore they withdraw from the interview for different personal reasons which made the non-response rate higher than the expected one. Of course

	the 95.% response rate is good for such type of study.<br><br>Question #19. The heading of Status of Knowledge, attitude, and preventive practice of COVID-19. It is better to avoid this title and write in their own parts as I comments above.<br>Response: Based on your comment we omitted this subheading and the contents were written in their own parts accordingly.<br>Question #20. Predictor is used for more advanced studies like cohort study. In this cross - sectional study, it is better to say associated factors.<br>Response: Yes, this is excellent idea too. The word predictor is replaced by associated factors throughout the revised version of the manuscript.<br><br>Question #21. Discuss only your pertinent findings like knowledge, attitude, and practice rather than discussing on sources of information towards COVID-19.<br>Response: Thank you very much for your comment. We tried to discuss only the pertinent finding of knowledge, attitude, and practices towards prevention of COVID-19. As a result, other less important points like sources of information are removed in the revised version of the manuscript.<br>Question #22. Why your justification becomes similar throughout your discussion? Please give reasonable justifications for each<br>Response: We tried to write specific justification for each variation in the finding of this study with other study finding<br><br>Question #23. Conclude based on your objective. Please also include the major factors affecting practice in the conclusion section. Finally, your recommendations should be based on your results. Does it mean, there is no problem on knowledge and attitude? Please rewrite it<br>Response: In the original version of the manuscript our emphasis was only on prevention practice of COVID-19 rather than knowledge and attitude of visitors towards COVID-19 prevention measures. But now the title is modified in to knowledge, attitude, and practice. Therefore the conclusion is amended according to finding of the study. (See the revised version of the manuscript.<br>Question #24. Avoid variables which contain a confidence interval of 1 in binary logistic regression.<br>Response: All variables in logistic analysis which contains a confidence interval of 1 are excluded<br>: Question #25. Check the figure digits<br>Response: We have accepted the comment and all figure digits are presented with two digit value<br><br><br>I hope that the revised manuscript is accepted for publication in PLoS ONE.<br><br>Sincerely yours,<br><br>Gete Berihun<br><br>Department of Environmental Health<br>Wollo University<br>Dessie, Ethiopia.
**Additional Information:**	
**Question**	**Response**
**Financial Disclosure**<br><br>Enter a financial disclosure statement that describes the sources of funding for the work included in this submission. Review the submission guidelines for detailed requirements. View published research articles from *PLOS ONE* for specific	The author(s) received no specific funding for this work

examples.<br><br>This statement is required for submission and **will appear in the published article** if the submission is accepted. Please make sure it is accurate.<br><br>**Unfunded studies**<br>Enter: *The author(s) received no specific funding for this work.*<br><br>**Funded studies**<br>Enter a statement with the following details:<br>• Initials of the authors who received each award<br>• Grant numbers awarded to each author<br>• The full name of each funder<br>• URL of each funder website<br>• Did the sponsors or funders play any role in the study design, data collection and analysis, decision to publish, or preparation of the manuscript?<br>• **NO** - Include this sentence at the end of your statement: *The funders had no role in study design, data collection and analysis, decision to publish, or preparation of the manuscript.*<br>• **YES** - Specify the role(s) played.<br><br>* typeset	
**Competing Interests**<br><br>Use the instructions below to enter a competing interest statement for this submission. On behalf of all authors, disclose any competing interests that could be perceived to bias this work—acknowledging all financial support and any other relevant financial or non-financial competing interests.<br><br>This statement **will appear in the published article** if the submission is accepted. Please make sure it is accurate. View published research articles from *PLOS ONE* for specific examples.	The authors have declared that no competing interests exist.

Powered by Editorial Manager® and ProduXion Manager® from Aries Systems Corporation

**NO authors have competing interests**<br><br>Enter: *The authors have declared that no competing interests exist.*<br><br>**Authors with competing interests**<br><br>Enter competing interest details beginning with this statement:<br><br>*I have read the journal's policy and the authors of this manuscript have the following competing interests: [insert competing interests here]*<br><br>* typeset	
**Ethics Statement**<br><br>Enter an ethics statement for this submission. This statement is required if the study involved:<br><br>• Human participants<br>• Human specimens or tissue<br>• Vertebrate animals or cephalopods<br>• Vertebrate embryos or tissues<br>• Field research<br><br>Write "N/A" if the submission does not require an ethics statement.<br><br>General guidance is provided below. Consult the submission guidelines for detailed instructions. **Make sure that all information entered here is included in the Methods section of the manuscript.**	The study was approved by the ethical review committee of Debre Tabor University. Permission to conduct the study was obtained from the respective hospital managers of the study site. Before the data collection, the purpose of the study was explained and verbal consent was obtained from each participant. Individuals who were volunteer to participate in the study were also told as they have the right to withdraw from the study at any stage of the interview. The confidentiality of the study participants was ensured by avoiding possible identifiers. Data collectors wear a facemask and keep a physical distancing of two feet. Facemask was provided for the study participants who did not wear during the data collection.

Powered by Editorial Manager® and ProduXion Manager® from Aries Systems Corporation

**Format for specific study types**<br><br>**Human Subject Research (involving human participants and/or tissue)**<br>• Give the name of the institutional review board or ethics committee that approved the study<br>• Include the approval number and/or a statement indicating approval of this research<br>• Indicate the form of consent obtained (written/oral) or the reason that consent was not obtained (e.g. the data were analyzed anonymously)<br><br>**Animal Research (involving vertebrate animals, embryos or tissues)**<br>• Provide the name of the Institutional Animal Care and Use Committee (IACUC) or other relevant ethics board that reviewed the study protocol, and indicate whether they approved this research or granted a formal waiver of ethical approval<br>• Include an approval number if one was obtained<br>• If the study involved *non-human primates*, add *additional details* about animal welfare and steps taken to ameliorate suffering<br>• If anesthesia, euthanasia, or any kind of animal sacrifice is part of the study, include briefly which substances and/or methods were applied<br><br>**Field Research**<br><br>Include the following details if this study involves the collection of plant, animal, or other materials from a natural setting:<br>• Field permit number<br>• Name of the institution or relevant body that granted permission	
**Data Availability**<br><br>Authors are required to make all data underlying the findings described fully available, without restriction, and from the time of publication. PLOS allows rare exceptions to address legal and ethical concerns. See the PLOS Data Policy and FAQ for detailed information.	Yes - all data are fully available without restriction

A Data Availability Statement describing where the data can be found is required at submission. Your answers to this question constitute the Data Availability Statement and **will be published in the article**, if accepted.<br><br>**Important:** Stating 'data available on request from the author' is not sufficient. If your data are only available upon request, select 'No' for the first question and explain your exceptional situation in the text box.<br><br>Do the authors confirm that all data underlying the findings described in their manuscript are fully available without restriction?	
**Describe where the data may be found in full sentences. If you are copying our sample text, replace any instances of XXX with the appropriate details.**<br><br>• If the data are **held or will be held in a public repository**, include URLs, accession numbers or DOIs. If this information will only be available after acceptance, indicate this by ticking the box below. For example: *All XXX files are available from the XXX database (accession number(s) XXX, XXX.*).<br>• If the data are all contained **within the manuscript and/or Supporting Information files**, enter the following: *All relevant data are within the manuscript and its Supporting Information files.*<br>• If neither of these applies but you are able to provide **details of access elsewhere**, with or without limitations, please do so. For example:<br><br>*Data cannot be shared publicly because of [XXX]. Data are available from the XXX Institutional Data Access / Ethics Committee (contact via XXX) for researchers who meet the criteria for access to confidential data.*<br><br>*The data underlying the results presented in the study are available from (include the name of the third party*	All relevant data are within the manuscript and its Supporting Information files.

and contact information or URL).<br>• This text is appropriate if the data are owned by a third party and authors do not have permission to share the data.<br><br>* typeset	
Additional data availability information:	

Powered by Editorial Manager® and ProduXion Manager® from Aries Systems Corporation

Manuscript

Click here to access/download;Manuscript;Manuscript.docx

Knowledge, Attitude, and Practices towards COVID-19 and associated factors among adult hospital Visitors in South Gondar Zone Hospitals, Northwest Ethiopia

Gete Berihun[1*,#], Zebader Walle Belete[2#], Awoke Keleb[1], Ayechew Ademas[1], Leykun Berhanu[1], Masresha Abebe[1], Adinew Gizeyatu[1], Seada Hassen[1], Daniel Teshome[3], Mistir Lingerew[2], Alelgne Feleke[2], Tarikuwa Natnael[2], Metadel Adane[1]

[1]Department of Environmental Health, College of Medicine and Health Sciences, Wollo University, Dessie, Ethiopia.

[2]Department of Public Health, College of Health Sciences, Debre Tabor University, Debre Tabor, Ethiopia.

[3]Department of Anatomy, College of Medicine and Health Sciences, Wollo University, Dessie, Ethiopia.

[*] **Corresponding author: geteberihun@gmail.com**

#Those authors contributed equally to this work

Abstract

Background

Coronavirus disease 2019 is currently the critical health problem of the globe, including Ethiopia. Visitors of healthcare facilities are the high-risk groups due to the presence of suspected and confirmed cases of coronavirus 2019 in healthcare setting. Increasing the knowledge, attitude and practices towards COVID-19 prevention among hospitals visitors are very important to prevent transmissions of the pandemic despite lack of evidence remains a challenge in Ethiopia. Therefore, this study was designed to investigate the status of knowledge, attitude, and practice towards COVID-19 and associated factors among hospital visitors in South Gondar Zone Hospitals, Northwest Ethiopia.

Methods

A facility-based cross-sectional study design was employed during August 1 to 30, 2020 from randomly selected 404 hospital visitors in South Gondar Zone Hospitals, northwest Ethiopia. Data was collected using interviewer administered questionnaire. The outcome of this study was good or poor knowledge, positive or negative attitude and good or poor practice towards COVID-19. A binary logistic regression model with 95% CI (Confidence interval) was used for data analysis. Bivariable analysis with (COR [crude odds ratio]) and multivariable analysis (AOR [adjusted odds ratio]) was used during data analysis. From the bivariable analysis, variables with a *p*-value <0.25 were retained into the multivariable logistic regression analysis. From the multivariable logistic regression analysis, variables with a significance level of *p*-value <0.05 were taken as factors independently associated with knowledge, attitude and practices towards COVID-19.

Main findings

About 69.3% of the respondents had good knowledge, 62.6% had positive attitude, and 49.3% had good practice towards the prevention of COVID-19. We found that factors significantly associated with good knowledge about COVID-19 were educational status who can read and write (AOR=2.78; 95%CI: 1.18, 6.56) and college and above (AOR=6.15; 95%CI: 2.18-17.40), and use of social media (AOR=2.96; 95%CI: 1.46, 6.01). Furthermore, factors significantly associated with positive attitude towards COVID-19 includes presence of chronic illnesses (AOR=5.00; 95%CI; 1.71-14.67), training on COVID-19 (AOR=3.91; 95%CI: 1.96-7.70), and peer/family as a source of information (AOR=2.45; 95%CI: 1.06-5.63). Being a student (AOR=7.70; 95%CI: 1.15-15.86) and participants who had a good knowledge on COVID-19 (AOR=4.49; 95%CI: 2.41-8.39) were factors significantly associated with good practice towards COVID-19.

Conclusion

We conclude that the status of knowledge, attitude and practice towards COVID-19 prevention was not satisfactory. Factors significantly associated with good knowledge were educational status who can read and write and college and above and use of social media. Factors significantly associated with positive attitude include presence of chronic illnesses, training on COVID-19, and peer/family as a source of information. Being a student and participants who had a good knowledge were factors significantly associated with good practice towards COVID-19. Hence, intervention strategies such as health education and infection prevention and control that could improve the knowledge, attitude and practice status towards COVID-19 preventions are urgently needed to control the transmission of COVID-19.

Keywords: Knowledge, attitude, practice, associated factors, COVID-19, Ethiopia

Introduction

Corona virus 2019 (COVID-19) is a rapidly emerging pandemic respiratory disease caused by a novel Coronavirus of Severe Acute Respiratory Syndrome (SARS/COV-2). The disease was reported initially in Wuhan city, Hubei Province, China at the end of December 2019 (1–3). Later on, World Health Organization (WHO) announces the disease as a public health emergency of international concern at the end of January 2020 and then declared as a global pandemic on March 11 (4–6). Two days later, the government of Ethiopia reported the first confirmed case of COVID-19 (7,8).

COVID-19 transmits mainly through droplets, airborne transmission, and contact between humans (6,9–11). The major sign and symptoms of COVID-19 cases are fever, dry cough, fatigue, myalgia, shortness of breath, and dyspnoea (4–6). The Severe cases of the disease may lead to the developments of cardiac injury, respiratory failure, acute respiratory distress syndrome, and death. Elders and patients with chronic medical illnesses like hypertension, cardiac disease, lung disease, cancer, or diabetes have been identified as potential risk factors for disease severity and mortality (6,11).

According to the Worldometer report, as of October 6, 2020, 9:54 am, COVID-19 spreads to more than 214 countries across the world. Worldwide, a total of 35,707,844 confirmed cases were reported. Of them, 26,907,997 were recovered and 1,049,700 were died of the pandemic (12). In case of Ethiopia, 79,437 confirmed cases of COVID-19 were reported. Of this, 1,230 and 34,016 were died and recovered, respectively (13).

Due to the absence of cure (6,11) prevention is recommended as the only strategy to prevent the spread of COVID-19. Different COVID-19 prevention measures are implemented such as respiratory hygiene, hand washing, social distancing, use of Personal Protective Equipment (PPE), and environmental disinfection (6,15–18). WHO designed different guidelines and online training sessions to increase the awareness of the community towards the prevention of the pandemic (19).But still, there is a deficiency of information mainly for vulnerable groups (6,18).

The government of Ethiopia has also implemented different prevention measures. Later on, the country declares and enforces a state of emergency for about six months since March 2020 But most populations of Ethiopia perceived that as the disease was eliminated since the termination of a state of emergency. Therefore, prevention measures towards COVID-19 are becoming neglected from time to time. Healthcare facilities are one the vulnerable area for transmission of COVID-19.As a result, visitors of healthcare facilities are one of the victim groups of population for COVID-19 due to close contact with suspected and confirmed cases of the disease. Even though many researchers were conducted on KAP towards prevention of COVID-19, there is limited evidence on KAP of healthcare facility visitors. Therefore, assessing knowledge, attitude, and practice are important measures for identifying gaps and taking of intervention accordingly. (4). Therefore, the study was designed to assess knowledge, attitude, and practice of adult visitors towards COVID-19 Prevention in South Gondar Zone Hospitals, Northwest Ethiopia.

Methods and materials

Study design, period, and setting

A health facility-based cross-sectional study design was applied among hospital visitors in South Gondar Zone Hospitals, Northwest Ethiopia during August 1 to 30, 2020. South Gondar zone is one of the 13 administrative zones in the Amhara regional state of Ethiopia. Debre Tabor is its capital town which is located at 597 km and 105 km from Addis Ababa and Bahir Dar, respectively. According to the population projection of 2014, the total population of the study area was 2,364,603 of which 1,196,318 were males while 1,168,285 were females (population projection 2014).

In South Gondar, there are one general hospital found in Debre Tabor Town and seven district government hospitals found in Addis Zemen, Mekane Eyesus, Andabet, Ebnat, Lay Gayint, Tach Gayint, and Smada towns. In addition, there are 98 government health centers, and 76 private clinics in South Gondar Zone (23). According to the reports of hospitals in South Gondar Zone, the average monthly visitors during COVID-19 were 13,440.

Source and study population

All hospital visitors whose age 18 years and above in South Gondar Zone Hospitals were the source population, whereas the study populations were those randomly selected study participants from the two randomly selected hospitals (Debre Tabor and Mekane Eyesus). Those study participants whose age below 18 years were excluded.

Sample size estimation and sampling methods

The sample size was determined using the single population proportion formula by taking the following assumptions.

$$n = \frac{(z_{a/2})^2 * p(1-p)}{d^2}$$

$Z_{\alpha/2}$ is the standard normal variable value at (1-α)% confidence level (α is 0.05 with 95% CI, $Z_{\alpha/2}$ = 1.96), an estimate of the proportion of knowledge attitude and practice, was considered as 50% as there was no similar studies conducted and 5% margin of error was considered. The sample size became 384 and after considering of 10% non-response rates, the adequate sample final sample size becomes 422.

After selecting the two hospitals randomly out of the 8 hospitals, we proportionally allocated sample size based on total estimated visitors of hospitals in the last three months. Then, 303 sample size was allocated for Debre Tabor general hospitals and 117 for Mekane Eyesus hospitals. Then hospitals visitors flow data during the previous 3 months in emergency ward, surgical ward, medical ward, gynecology/obstetrics ward and pediatrics ward considered for sample size allocation for each hospitals departments. Finally, randomly selection of visitors for each ward was selected until the allocated sample size achieved.

Outcome variable measurement

The outcome variable was good or poor knowledge, positive or negative attitude and good or poor practice towards COVID-19.

Good or poor knowledge: Knowledge was measured by using 15 questions consisting of signs and symptoms, risk groups and prognosis, method of transmission, and /preventive methods. Each question was consisted of as 'Yes', 'No', and 'I do not know' options. Respondents who answered correctly were given 1 point while others were given 0 points. The total knowledge score ranges from 0-15 and a cut-off level of ≥12 (80% and above) was considered as good knowledge while <12 (80%) was considered as poor knowledge towards COVID-19 prevention (24).

Positive or negative attitude: Attitude was measured by using 11 items and the response was categorized based on 3 scale measurements with agree (3 points), neutral (2 points), and disagree (1 point). The score of attitude varied from 11 to 33, with an overall score of ≥ 27 (81.8%) was considered as a positive attitude, whereas a score of less than 27 (81.8%) was considered as negative attitude towards COVID-19 prevention (27).

Good or poor practice: The practice was measured using 10 items and those who respond as yes were given 1 point while no was marked as 0. The total prevention practice score ranges from 0-10 and a score with a cut-off ≥ 8 (80%) was considered as good practice while <8 was taken as a poor practice (25,26).

Data collection and quality assurance

Data were collected using a pre-tested structured questionnaire which was adapted from published articles in reputable journals and from WHO COVID-19 guidelines (28–33). The questionnaire consists of five sections including; part I: socio-demographic characteristics of the participants; part II; Pre-existing medical condition and sources of information towards COVID-19; part III: knowledge of the participants; part IV: Attitude of the participants; and part V:

Prevention practice of COVID-19. The tool was prepared in the English version and translated to Amharic version (local language), and re-translated back to English to ensure consistency. The tool was pre-tested using 5% of the final sample size in Andabet district hospital visitors to establish the validity of the questionnaire. Based on the pre-test, appropriate amendments such as order arrangement of questions, editing of unclear questions, and avoiding irrelevant questions were done accordingly.

The data was collected using interviewer administered method using four BSc nurse professionals and supervised by two Public Health experts. Two days of training was given for data collectors and supervisors on the overall aim of the study, contents of the tool and ethical issues. Supervision was carried out on daily basis, and appropriate corrections of the collected data were done accordingly. Furthermore, double data entry was done to control data entry errors and data cleaning was carried before statistical analysis. The reliability coefficient of Cronbach's alpha was 0.76 which is an acceptable range.

Statistical analysis

Data was entered into EpiData version 4.6 and exported to the Statistical Package of the Social Science (SPSS) version 25.0 for data cleaning analysis. Descriptive statistic such as frequencies and percentages were calculated for categorical variables and mean with standard deviations for continuous variables to examine the overall distribution.

Associations between independent variables and knowledge, attitudes and practices towards COVID-19 were determined using a binary logistic regression model at 95% CI (Confidence interval). We used three different logistic regression models: The first model (Model 1) identified factors associated with good knowledge about COVID-19, the second model (Model

II) identified factors associated with favorable attitudes and the third model (Model III) identified factors associated factors with good preventive practices towards COVID-19. For each model, bivariable analysis with (COR [crude odds ratio]) and multivariable analysis (AOR [adjusted odds ratio]) was used.

From the bivariable analysis, variables with a p-value <0.25 were retained into the multivariable logistic regression analysis. From the multivariable analysis of each model, variables with a significance level of p-value <0.05 were taken as factors independently associated with knowledge, attitude and practices towards COVID-19. The presence of multicollinearity among independent variables was checked using standard error at the cutoff value of 2 and we found that a maximum standard error of 0.97, which indicated no multi-collinearity. Model fitness was checked using the Hosmer-Lemeshow test for Model I, Model II and Model III and found a p-value of 0.650, 0.871 and 0.913, respectively and indicated that all models were fit.

Ethics approval and consent to participate

The study was approved by the ethical review committee of Debre Tabor University. Permission to conduct the study was obtained from the respective hospital managers of the study site. Before the data collection, the purpose of the study was explained and verbal consent was obtained from each participant. Individuals who were volunteer to participate in the study were also told as they have the right to withdraw from the study at any stage of the interview. The confidentiality of the study participants was ensured by avoiding possible identifiers. Data collectors wear a facemask and keep a physical distancing of two feet. Facemask was provided for the study participants who did not wear during the data collection.

Result

Socio-demographic characteristics of hospital visitors

A total of 404 visitors participated in the study with a response rate of 95.7%. Nearly two-thirds 242 (59.7%) of the study participants were females and about one-fifth 92 (22.8%) of the the hospital visitors were in the age range of 30-39 years. Furthermore, 66 (16.3%), 68(16.8%), and 117(29.0%) of the respondents can't read and write, farmers, and live in rural areas respectively. Finally, 56 (13.9%) of the participants had either or more chronic medical illness history (Table 1).

Knowledge of hospital visitors towards COVID-19 prevention

More than two-thirds 280 (69.3%; 95%CI; 65.1-73.8%) of the visitors had a good knowledge, whereas 124 (30.7%; 95%CI: 27.2-34.9%) of them had poor knowledge about COVID-19. Almost all 388 (96.0%) of the participants heard about COVID-19 and more than three-fourths 322 (79.7%) of the participants knew as COVID-19 is a viral disease and 339 (83.9%) of them knew the major sign and symptoms of COVID-19 cases. Furthermore, more than three-fourth 320 (79.2%) of the participants knew that elders, those who had a chronic medical illness, and being obese are more likely to have severe cases of COVID-19. Similarly, 283 (70.0%) of the respondents knew that COVID-19 can be transmitted from one person to another even in the absence of COVID-19 (Table 2).

Attitude of hospital visitors towards COVID-19 prevention

About two-thirds 62.6% (95%CI;57.2-67.6%) of the hospital visitors had a positive attitude towards COVID-19 prevention, whereas 37.4% (95%CI: 32.4-42.8%) had negative attitude

towards COVID-19 prevention. About half 203 (50.2%) of the participants agree that the black race is not protective against COVID-19. Similarly, less than half 180 (44.6%) of the participants agreed that Ethiopia is in a good position to contain the spread of the COVID-19. About two-thirds 274 (67.8%) of the participants believed COVID-19 do not cause stigma. More than half 221(54.7%) of the respondents agree that they can get infected with COVID-19 if they contacted infected patients despite their good immunity. On the other hand, 55 (13.6%) of the respondents believed that COVID-19 has occurred as a result of our sin (Table 3**)**.

Preventive practice of visitors towards COVID-19 prevention

Half of the respondents 199 (49.3%) practiced the recommended COVID-19 prevention methods. Majority 378 (93.6%) of the participants washed their hands with water and soap for at least 20 seconds. Furthermore, almost nine out of ten respondents avoid handshaking practice for prevention of COVID-19. But a relatively lower number of 338 (83.7%) participants used facemasks when they leave their home and 333 (82.4%) practiced respiratory hygiene while coughing and sneezing. Furthermore less than half 177(43.8%) participants applied keeping of the recommended physical distance for prevention of COVID-19. Staying at home was also another challenge and only less than one-third 121(30%) of the participants applied it (Table 4).

Factors Associated with Knowledge, Attitude, and practice towards COVID-19 from multivariable analysis

A multi-variable analysis from the first model indicated that educational status and use of social media as a source of information were statistically significant with the knowledge of COVID-19. The fining revealed that those who can read and write were 2.78 times more likely to have good knowledge towards COVID-19 prevention methods than those who could not read and write. Similarly, participants who have college and above educational level were 6.15 (AOR = 6.15; 95%CI: 2.18-17.40) times more likely to have good knowledge than those who could not read and write. Furthermore, participants who used social media as a source of information towards COVID-19 was 2.96 (AOR = 2.96; 95%CI: 1.46-6.01) times more likely to have good knowledge than those who did not use social media (Table 5).

A multi-variable analysis from the second model revealed that those who had primary education were 6.49 (AOR = 6.49; 95%CI: 1.52-27.78) times more likely to have a positive attitude than those who could not read and write while being college and above graduated were 6.91 (AOR = 6.91; 95%CI: 2.58-14.5) times more likely to have a positive attitude than the corresponding reference group. Furthermore, visitors who had chronic medical illnesses were 5 times (AOR = 5; 95%CI: 1.71-14.67) more likely to have a positive attitude than those who did not have a chronic illness. Furthermore, participants who took training on COVID-19 prevention were 3.9 (AOR = 3.9; 95%CI: 1.96-7.70) times more likely to have a positive attitude than those who didn't take the training. Additionally, participants who used peer as a source of information towards COVID-19 prevention were 2.45 (AOR = 2.45; 1.06-5.63) times more likely to have a

positive attitude than those who didn’t use peers as a source of information for COVID-19 prevention (Table 6).

From multivariable analysis of model three, we found that being a student was 7.7 times (AOR = 7.7; 95%CI: 1.15-51.86) more likely to have a good practices than farmers. Furthermore, participants who had good knowledge were 4.49 (AOR = 4.49; 95%CI: 2.41-8.39) times more likely to have a good practice about COVID-19 prevention than those who poor knowledge (Table 7).

Discussion

The pandemic of COVID-19 is still the critical concern of the globe including our country Ethiopia. But up to date, there is no confirmed treatment for the pandemic. Therefore prevention is the single most important method of alleviating the spread of COVID-19 pandemic.

In this finding, about 81.67% of the knowledge questions were correctly replied to by the respondents. This finding was in line with the study conducted in Saudi Arabia (80.5%) (4) and in Nigeria (77.36) (35). The finding of this study was lower than the study conducted in China (90%) (36). This discrepancy may be due to variation in the study population's characteristics, government commitment, and health care system. On the contrary, this study result was higher than in the Egyptian population (71.26%) (37). This discrepancy might be due to the variation in socio-demographic characteristics of the population.

The finding of this study revealed that 69.3 % (CI; 65.1-73.8) of the participants had good knowledge towards COVID-19 prevention which was in line with the study conducted in India (70 %) (38). On the other hand, this study finding was lower than a multicenter study conducted among health care workers in Ethiopia with 88.2% (16) and Nigerian residents in an urban

setting (99.7%) (39). This deviation may be due to variations in socio-demographic characteristics of the study population and sources of information towards COVID-19.

This study also revealed that about 80% of participants knew that the elderly, those who had chronic medical illnesses, and obese are more likely to develop severe cases of COVID-19. This finding was slightly higher than the study conducted in Ethiopia (72.5%) (11**).** This variation may be due to the change in study period, socio-demographic characteristics of the study population, and coverage of awareness creation towards COVID-19 prevention. Even though children and young adults are vulnerable groups, only 83.4% of the participants knew that these groups need to take preventive measures towards COVID-19. Neglecting such types of the population may wide-spreading the transmission of the pandemic (11).

Regarding the attitudes, 62.6% (95% CI; 57.2-67.6) of respondents had a positive attitude towards COVID-19 prevention which was lower than the study conducted in Ethiopia (94.7%) (16), Nigeria 79.5% (39), and Pakistan (82.16%) (40). This discrepancy may be due to a change in the socio-demographic characteristics of the study population, government commitment towards COVID-19. On the other hand, less than half (44.6%) of the participants believed that the government of Ethiopia can control the spread of COVID-19 within a short time. This finding was lower than the study conducted in china 97.1% (24) and India at 87.2% (41). This deviation may be due to the variation in the quality of the health care system, socio-demographic characteristics of the study population, and government preparedness towards the control of COVID-19 pandemic. According to the WHO report, the government of Ethiopia scored 52% towards COVID-19 preparedness response (21) which supports the finding of this study. Furthermore, this study also indicated that almost two-thirds of the respondents believed that the pandemic of COVID-19 leads to the development of social stigma which was lower than a study

conducted in Ethiopia at 77% (16) and 83.8% (11). This deviation may be due to differences in socio-demographic characteristics of the study population and study period. On the contrary, this study finding was higher than the study conducted in the Peruvian population 59.1% (42). This variation may be due to a change in the socio-demographic characteristics of the study population, study period, awareness creation towards COVID-19, and the burden of the pandemic. The social stigma may be developed due to fear of its mortality and high communicability. The history of social stigma due to pandemic was not a new phenomenon (43,44).

Regarding the prevention practice of COVID-19, the overall practice score of the respondents was 73.2% which was higher than the study conducted in Ethiopia (26). The finding of this study revealed that only half of the respondents 49.3% had a positive preventive practice of COVID-19. The finding of this study was lower than other studies conducted in Ethiopia (16,26) and China (45). This variation may be due to the change in the study setting, socio-demographic characteristics of the study population, and occupation of the study participant (being a health professional vs. general population), and the commitment of the government towards the prevention of COVID-19. Furthermore, most of the participants 93.6% washed their hands with water and soap for at least 20 seconds which was in line with a study conducted in Nigeria 96.4% (39). On the contrary, this finding was lower than a study conducted in Nigeria 87.9 % (35). This deviation may be to a change in access and utilization of handwashing facilities in health care facilities.

Furthermore, 83.7% of the participants used face masks for prevention of COVID-19 which were consistent with the study conducted in Nigeria 84.4% (35), and 82.3% (39). This finding also revealed that less than half (43.8%) of the respondents applied the recommended physical

distance of 2 meter when they go to public crowded areas. This finding was lower than the study conducted in Nigeria 83% (35) and 92.7%, (39). This variation may be due to a change in the socio-demographic characteristic of the study population, the burden of the disease, awareness of the community towards the COVID-19 pandemic, and population way of life.

The finding also revealed that more than two-thirds 70% of the respondents avoid going to crowded places after the emergence of COVID-19 which was higher than the finding in Nigeria 58.9% (35). in addition to this, 82.4% of the respondents practiced respiratory hygiene which was lower than the finding in India (97.7%) (46). The variation might be due to a change in a study setting, heterogeneity of population perception of the community, knowledge towards COVID-19, and burden of confirmed COVID-19 cases. Above all, the most common problem which was not applied by the participants was staying at home and only less than one-third (30%) after the occurrence of COVID-19. The possible justification for this might be due to the uncontrolled of the pandemic for a long period of time, poor preparedness of the community towards the pandemic, and subsistence way of life of the community.

Conclusion

We conclude that the status of knowledge, attitude and practice towards COVID-19 prevention was not satisfactory. Factors significantly associated with good knowledge about COVID-19 were educational status who can read and write and college and above and use of social media. Factors significantly associated with positive attitude towards COVID-19 include presence of chronic illnesses, training on COVID-19, and peer/family as a source of information. Being a student and participants who had a good knowledge on COVID-19 were factors significantly associated with good practice towards COVID-19. Hence, intervention strategies that could

improve the knowledge, attitude and practice status towards COVID-19 preventions are urgently needed to control the transmission of COVID-19. Health education about COVID-19 infection prevention and control methods and advocacy about COVOD-19 recommended prevention using various media channels are highly recommended. Therefore, hospital administrators should work in collaboration with other concerned stakeholders to enhance knowledge, attitude, and practice of hospital visitors to wards COVID-19 prevention.

Abbreviations

COVID-19: Coronavirus disease 2019; AOR: Adjusted Odds Ratio; CI: Confidence Interval; COR: Crude Odds Ratio; PPE: Personal Protective Equipment; SARAS_COV-1: Severe Acute Respiratory Syndrome- Corona Virus-1; SARS-COV-2: Severe Acute Respiratory Syndrome-Corona Virus-2

Acknowledgments

Our heartfelt gratitude extends to Debre Tabor University for providing ethical clearance for this study. Debra Tabor and Mebane Eyesus hospital administrators are duly acknowledged for their permission to conduct this study and for the information we received while we needed. We also thank the study participants who gave valuable information and their kind cooperation during this study. We are also grateful for data collectors and supervisors for their commitment during the data collection.

References

1. Elhadi M, Msherghi A, Alkeelani M, Zorgani A, Zaid A, Alsuyihili A, et al. Assessment of healthcare workers' levels of preparedness and awareness regarding Covid-19 infection in low-resource settings. Am J Trop Med Hyg. 2020;828–33.

2. WHO Regional Office for Africa. COVID-19 Situation Update for WHO African Region, External Situation Report. 2020 Jun. Report No.: 15.

3. Saqlain M, Munir MM, Ur Rehman S, Gulzar A, Naz S, Ahmed Z, et al. Knowledge, attitude, practice and perceived barriers among healthcare professionals regarding COVID-19: A Cross-sectional survey from Pakistan. Occupational and Environmental Health; 2020 Apr.

4. Al-Hanawi MK, Angawi K, Alshareef N, Qattan AMN, Helmy HZ, Abudawood Y, et al. Knowledge, Attitude and Practice Toward COVID-19 Among the Public in the Kingdom of Saudi Arabia: A Cross-Sectional Study. Front Public Heal. 2020;8(May).

5. Iannone P, Castellini G, Coclite D, Napoletano A, Fauci AJ, Iacorossi L, et al. The need for health policy perspective to protect Healthcare Workers during COVID-19 pandemic. A GRADE rapid review on the effectiveness of the N95 respirator. PLoS One [Internet]. 2020;15(6):1–13. Available from: http://dx.doi.org/10.1371/journal.pone.0234025

6. Nallani VRR, Nadendla RR, Kavuri NSS. Knowledge, attitude, and practice among health care professionals regarding COVID-19 and barriers faced by health care professionals inSouth India. Int J Community Med Public Heal. 2020;7(9):3450.

7. Lone SA, Ahmad A. COVID-19 pandemic – an African perspective. :10.

8. Baye K. COVID-19 prevention measures in Ethiopia Current realities and prospects. The Federal Democratic Republic of Ethiopia Policy Studies Institute; 2020.

9. Ssebuufu R, Katembo Sikakulya F, Binezero SM, Wasingya L, Nganza SK, Ibrahim B, et al. Awareness knowledge attitude and practice towards measures for prevention of the spread of COVID-19 in the Ugandans A nationwide online cross-sectional Survey. Medrxiv. 2020;1–28.

10. WHO. COVID-19 Strategic Preparedness and Response Plan Operational Planning Guideline to Support Country Preparedness and Response. 2020.

11. Kebede Y, Yitayih Y, Birhanu Z, Mekonen S, Ambelu A. Knowledge, perceptions and preventive practices towards COVID-19 early in the outbreak among Jimma university medical center visitors,Southwest Ethiopia. PLoS One [Internet]. 2020;15(5):1–15. Available from: http://dx.doi.org/10.1371/journal.pone.0233744

12. Worldometer Report COVID-19 coronavirus pandemic 2020. https://www.worldometers.info/coronavirus, 2020 accessed date October 6, 2020, 9.54 am

13. Worldometer report.COVID 19 coronavirus pandemic 2020. https://www.worldometers.info/coronavirus/country/ethiopia,2020 accessed date October 6, 2020, 9.54 am

14. Nigussie T.F and Azimach N.N. Knowledge, Attitude and Practice towards COVID-19 among Arba Minch Town GSJ : Volume 8, Issue 6, June 2020, Online : ISSN 2320-9186

15. CDC. Infection prevention and control for COVID-19 in healthcare settings - first update 12 March 2020. Eur Cent Dis Prev Control. 2020;(March):1–10.

16. Jemal B, Ferede ZA, Mola S, Hailu S, Abiy S, Wolde GD, et al. Knowledge, attitude, and practice of healthcare workers towards COVID-19 and its prevention in Ethiopia: a multicenter study. In Review; 2020 May.

17. Olum R, Chekwech G, Wekha G, Nassozi DR, Bongomin F. Coronavirus Disease-2019: Knowledge, Attitude, and Practices of Health Care Workers at Makerere University Teaching Hospitals, Uganda. Front Public Heal. 2020 Apr;8:181.

18. Lakshmi Priyadarsini S, Suresh M. Factors influencing the epidemiological characteristics of pandemic COVID 19: A TISM approach. Int J Healthc Manag. 2020;13(2):89–98.

19. Saqlain M, Munir MM, Rehman SU, ... Knowledge, attitude, practice and perceived barriers among healthcare professionals regarding COVID-19: A Cross-sectional survey from Pakistan. J … [Internet]. 2020; Available from https://www.ncbi.nlm.nih.gov/pmc/articles/PMC7211584/

20. Goshu D, Ketema M. Assessment of COVID-19 Effects and Response Measures in Ethiopia : Livelihoods and Welfare Implications Assessment of COVID-19 Effects and Response Measures in Ethiopia : Livelihoods and Welfare Implications 1. 2020.

21. Tolu LB, Ezeh A, Feyissa GT. How Prepared Is Africa for the COVID-19 Pandemic Response ? The Case of Ethiopia What Measures Should Be Taken to. 2020;

22. Alemayehu TY, Boled AT, Taye MG, Kibret S. Assessment of emergency and essential surgical service in primary hospitals ofSouth Gondar zone, Amhara region, Ethiopia, 2019 International Journal of Surgery Open Assessment of emergency and essential surgical service in primary hospitals ofSouth Gondar zone, Amhara region, Ethiopia, 2019. Int J Surg Open [Internet]. 2020;23(February):35–8. Available from: https://doi.org/10.1016/j.ijso.2020.02.003

23. Abate BA, Ahunie MA. Basic Emergency Maternal And Neonatal Care Status OfSouth Gon- Dar Zone, North Central Ethiopia: Institutional Descriptive Sur- Vey, June 2016. 2017;9(1):11.

24. Giao H, Han NTN, Khanh T Van, Ngan VK. Knowledge and attitude toward COVID-19 among healthcare workers at District 2 Hospital, Ho Chi Minh City. Asian Pac J Trop Med. :7.

25. Defar A, Molla G, Abdella S, Tessema M, Ahmed M, Tadele A et. al. Knowledge, Practice and associated factors towards the Prevention of COVID-19 among high-risk groups: A cross-sectional study in Addis Ababa, Ethiopia. 2020;19.

26. Asemahagn MA. Factors determining the knowledge and prevention practice of healthcare workers towards COVID-19 in the Amhara region, Ethiopia : a cross-sectional survey. 2020;3.

27. Salman M, Mustafa ZU, Asif N, Zaidi HA, Shehzadi N. Knowledge, attitude and preventive practices related to COVID-19 among health professionals of Punjab province of Pakistan.

28. WHO E. Survey Tool And Guidance Rapid, simple, flexible behavioral insights on COVID-19. 2020.

29. Ahmad NA, Ahmad W, Arman R, Rahimi W, Ahmadi A, Vijay Raghavan SMS. Community Perception Survey – COVID 19 Knowledge, Attitude, and Practice Survey in Kabul, Kunduz, and Khost Province. the JOHANNITER international assistance, JACK, OHW; 2020.

30. National Comprehensive Covid19 Management Handbook. :162.

31. National Institute of Environmental Health Science. COVID-19_BSSR_Research_Tools.pdf. 2020.

32. Admissions R, Homes R. COVID-19 : Infection Prevention and Control Checklist for Long-Term Care and Retirement Homes. 2020;1–15.

33. WHO. COVID-19 Infection Prevention and Control Sameeksha. 2020;2.

34. Ahmed N, Shakoor M, Vohra F, Abduljabbar T, Mariam Q, Rehman MA. Knowledge, Awareness, and Practice of Health care Professionals amid SARS-CoV-2, Corona Virus Disease Outbreak. :8.

35. Okoro J, Ekeroku A, Nweze B, Odionye T, Nkire J, Onuoha M, et al. Attitude and preventive practices towards COVID-19 disease and the impact of awareness training on knowledge of the disease among correctional officers [version 1 ; peer review : awaiting peer review]. 2020;1–10.

36. Zhong B, Luo W, Li H, Zhang Q, Liu X, Li W, et al. Knowledge, attitudes, and practices towards COVID-19 among Chinese residents during the rapid rise period of the COVID-19 outbreak : a quick online cross-sectional survey. 2020;16.

37. Abdelhafiz AS, Mohammed Z, Ibrahim ME, Ziady HH, Alorabi M, Ayyad M, et al. Knowledge, Perceptions, and Attitude of Egyptians Towards the Novel Coronavirus Disease (COVID-19). J Community Health [Internet]. 2020;45(5):881–90. Available from: https://doi.org/10.1007/s10900-020-00827-7

38. Singh JP, Sewda A. Assessing the Knowledge, Attitude, and Practices of Students Regarding the COVID-19 Pandemic. 2020;

39. Christopher R, Margaret R, Dauda MAD, Saleh A, Ene P. Knowledge, Attitudes and Practices Towards COVID - 19 : An Epidemiological Survey in North - Central Nigeria. J Community Health [Internet]. 2020; (0123456789). Available from: https://doi.org/10.1007/s10900-020-00881-1

40. Hussain I, Majeed A, Imran I, Ullah M, Hashmi FK, Saeed H, et al. Knowledge, Attitude, and Practices Toward COVID - 19 in Primary Healthcare Providers : A Cross-Sectional Study from Three Tertiary Care Hospitals of Peshawar, Pakistan. J Community Health [Internet]. 2020;(0123456789). Available from: https://doi.org/10.1007/s10900-020-00879-9

41. S DP, B KR, Athul K, Swamy S, Bhodaji S, Deshmukh A, et al. Knowledge, Attitude, Awareness and Practice Towards Covid-19 Pandemic in Indian Citizens During The National Lockdown Period : A Quick Online Cross-. 2020;8(May):3504–15.

42. Zegarra-Valdivia, J. A. 1; Chino-Vilca, B. N. 1; & Ames-Guerrero R. Knowledge, perception and attitudes regarding COVID-19 Pandemic in Peruvian Population.

43. Person B, Sy F, Holton K, Govert B, Liang A, Sars N. Fear, and Stigma : The Epidemic within the SARS Outbreak. 2004;10(2).

44. Paget J. ITALIAN OF PUBLIC HEALTH The influenza pandemic and Europe : the social impact and public health response OF PUBLIC. 2009;6(3):257–9.

45. Zhou M, Tang F, Wang Y, Nie H, Zhang L, You G, et al. Knowledge, attitude and practice regarding COVID-19 among health care workers in Henan, China. J Hosp Infect [Internet]. 2020; Available from https://doi.org/10.1016/j.jhin.2020.04.012

46. Awasthi AA, Taneja N, Janardhanan R. Knowledge, Attitude And Practice Towards Covid-19 Pandemic Among Residents Of Bihar, India. 2020;11(D):38990–5.

Table 1: Socio-demographic characteristics of adult visitors in hospitals of South Gondar zone Northwestern Ethiopia, August 1 to 30, 2020

**Variable**	**Category**	**Frequency (*n*)**	**Percentage (%)**
Sex	Male	163	40.3
	Female	241	59.7
Age(years)	<20	24	5.9
	20-29	92	22.8
	30-39	111	27.5
	40-49	94	23.3
	50-59	48	11.9
	≥60	35	8.7
Religion	Muslim	30	7.4
	Orthodox	331	81.9
	Protestant	43	10.6
Marital status	Single	83	20.5
	Married	295	73.1
	Divorced	26	6.5
Educational status	Cannot read and wright	66	16.3
	Read and write	95	23.5
	Primary (1-8) grade	36	8.9
	Secondary (9-12)grade	29	7.2
	College and above	178	44.1
Occupation	Farmer	68	16.8
	Student	45	11.1
	Unemployed	55	13.6
	Government employer	129	31.9
	Private business worker	107	26.5
Resident	Urban	287	71.0
	Rural	117	29.0
Monthly income(ETB)	≤499	127	31.4
	500-2000	101	25
	≥2001	176	43.6
History of chronic medical illness	Yes	56	13.9
	No	348	86.1
Training on COVID 19	Yes	137	33.9
	No	267	64.1
Use social media	Yes	252	62.4
	No	152	37.6
Peer as a source of information of COVID19	Yes	345	85.4
	No	59	14.6
Use TV/radio as a source of information	Yes	321	79.5
	No	83	20.5
Use religious institution as source of information	Yes	130	32.2
	No	274	67.8

Table 2: Knowledge of hospital visitors towards COVID-19 prevention in hospitals of South Gondar zone, Northwestern Ethiopia, August 1 to 30, 2020

Item	Yes		No		I do not know	
	Frequency	Percent	Frequency	Percent	Frequency	Percent
	n	%	n	%	*n*	%
Did you hear about COVID-19?	388	96.0	16	4.0		
COVID-19 is a viral disease.	322	79.7	40	9.9	42	10.4
The major sign and symptoms of COVID-19 are dry cough, fever, and shortness of breathing.	339	83.9	33	8.2	32	7.9
Runny nose and sneezing are less common symptoms of COVID-19.	275	68.1	88	21.8	41	10.1
Elder, those who have a chronic medical illness and obese are more likely to sever the case of COVID- 19.	320	79.2	57	14.1	27	6.7
Currently, there is no effective cure for COVID-19.	331	81.9	50	12.4	23	5.7
COVID-19 virus can spread via respiratory droplets.	375	92.8	29	7.2		
Eating and contacting wild animals would result COVID-19 infection	308	76.2	53	13.1	43	10.6
Persons with COVID 19 virus can transmit the virus to others when a fever is not present	283	70.0	71	17.6	50	12.4
Proper washing hand with soap and water is one method of preventing COVID-19.	375	92.8	18	4.5	11	2.7
Wearing general masks can prevent one from acquiring	354	87.6	35	8.7	15	3.7

infection by the COVID 19 virus						
Children and young adults must take measures to prevent the infection by Covid 19 virus	337	83.4	45	11.1	22	5.4
To prevent the infection by COVID 19 virus individuals should avoid going to crowded places such as bus parks and avoid public transportation	352	87.1	50	12.4	2	.5
People who have contact with someone infected with COVID 19 virus should be immediately isolated in a proper place in general the observation period is 14 days	273	67.6	95	23.5	36	8.9
Isolation and treatment of people who are infected with the COVID 19 virus are effective ways to reduce the spread of the virus	295	73.0	80	19.8	29	7.2

Mean± standard deviation = **12.25±2.45; Minimum = 2 and maximum = 15**

Table 3:-Attitude of adult visitors towards COVID-19 prevention in hospitals of South Gondar zone, Northwest Ethiopia, August 1 to 30, 2020

Questions	Agree	Neutral	Disagree
Black races are not protected from COVID 19 disease.	203(50.2%)	146(36.1%)	55(13.6%)
Wearing a well-fitting face mask are effective in preventing COVID 19 virus	268(66.3%)	81(20.0%)	55(13.6%)
Hand wash can prevent you from COVID 19 virus	321 79.5	77 19.1	6 1.5
Ethiopia is in a good position to contain COVID 19 virus	180 44.6	144 35.6	80 19.8
COVID 19 is not stigma and I should not hide my infection	274 67.8	90 22.3	40 9.9
If I get infected with COVID 19, I will go to the hospital as advised.	221 54.7	141 34.9	42 10.4
I can get infected with COVID 19 if I contacted an infected patient despite my good immunity.	230 56.9	100 24.8	74 18.3
COVID 19 is fatal	215 53.2	105 26.0	84 20.8
During the outbreak of COVID 19 eating well cooked and safely handled meat is safe.	249 61.6	96 23.8	59 14.6
COVID 19 patients should share their recent travel history with a health care provider.	256 63.4	85 21.0	63 15.6
Do you think that the cause of Covid-19 is not spiritual/ is it happened because of our sin?	262 64.9	87 21.5	55 13.6

***** Mean ±standard deviation = **27.11±4.08**; Minimum = **17**; Maximum = **33**

Table 4:-Preventive practice of adult visitors towards COVID-19 prevention in hospitals of South Gondar zone, Northwest Ethiopia, August 1 to 30, 2020

Questions	Yes		No	
	Frequency		Frequency	
	n	%	N	%
Do you avoid handshaking to prevent covid 19?	363	89.9	41	10.1
Have you washed your hands often with soap and water for at least 20 seconds especially after you have been in a public place or after blowing your nose, coughing, or sneezing?	378	93.6	26	6.4
If soap and water are not readily available, are you applying a hand sanitizer that contains at least 60% alcohol?	309	76.5	95	23.5
Do you wear face masks repeatedly when you leave your home?	338	83.7	66	16.3
Do you coughing and sneezing into the elbow or within clothing?	333	82.4	71	17.6
In recent days have you avoid going to any crowded place?	281	69.6	123	30.4
Do you avoid eating raw animal products to prevent the COVID 19 virus?	336	83.2	68	16.8
Do you avoid touching your mouth nose and eyes with unwashed hands?	323	80.0	81	20.0
Do you keep your self 2m away from the others when you got to the public area?	177	43.8	227	56.2
Do you stay at your home after the emergent of covid 19?	121	30.0	283	70.0
Mean ±standard deviation **7.32±1.60**				
Minimum **1.00**				
Maximum **10**				

Table 5:-Associated factors of knowledge towards COVID-19 prevention among adult visitors in hospitals of South Gondar zone, Northwest Ethiopia, August 1 to 30, 2020

**Variable**	**Knowledge status**		**COR(95% CI)**	**AOR(95% CI)**	**P-value**
	**Good**	**Poor**			
Age					
<20	12	12	1	1	
20-29	70	22	3.18(1.25-8.09)	1.98(0.54-7.29)	0.31
30-39	82	29	2.83(1.14-6.99)	1.08(0.30-3.87)	0.091
40-49	58	39	1.61(0.65-3.97)	1.06(0.31-3.68)	0.92
50-59	33	15	2.20(0.80-6.02)	1.21(0.30-4.82)	0.79
≥60	25	10	2.50(0.84-7.40)	0.69(0.16-2.95)	0.61
Marital status					
Single	61	22	1	1	
Married	205	90	0.82(0.48-1.42)	0.88(0.42-1.82)	0.72
Divorced	14	12	0.42(0.17-1.05)	0.92(0.29-2.98)	0.89
**Education**					
cannot read and write	25	41	1	1	
Read and write	54	41	2.16(1.14-4.12)	**2.78(1.18-6.56)***	**0.02***
Primary	26	10	4.26(1.76-10.31)	2.42(0.56-10.44)	0.24
Secondary	21	8	4.31(1.66-11.18)	1.54(0.25-9.56)	0.65
College and above	154	24	10.52(10.52-5.45)	**6.15(2.18-17.40)***	**0.001***
Occupation					
Farmer	23	45	1	1	
Student	33	12	5.38(2.35-12.34)	1.64(0.28-9.72)	0.59
Currently unemployed	38	17	4.37(2.04-9.36)	1.50(0.49-4.58)	0.48
Gov't worker	111	18	12.07(5.95-24.48)	0.83(0.16-4.19)	0.82
Private business	75	32	4.59(2.39-8.80)	0.91(0.25-3.30)	0.89
Resident					

Urban	216	71	2.52(1.60-3.96)	1.43(0.75-2.71)	0.281
Rural	64	53	1	1	
Monthly income					
<499	73	54	1	1	
500-2000	67	34	1.46(0.85-2.51)	1.272(0.52-3.09)	0.600
>2000	140	36	2.88(1.73-4.78)	1.29(0.46-3.60)	0.630
Training					
Yes	113	24	2.82(1.70-4.67)	1.74(0.89-3.42)	0.110
No	167	100	1	1	
Use social media					
Yes	204	48	4.25(2.72-6.65)	**2.96(1.46-6.01)***	**0.003***
No	76	76	1	1	
Peer					
Yes	252	93	3.00(1.71-5.27)	1.09(0.48-2.51)	0.840
No	28	31	1	1	
TV/radio					
Yes	243	78	3.87(2.34-6.40)	1.07(0.43-2.65)	0.88
No	37	46	1	1	
Religious institution					
Yes	98	32	1.55(0.97-2.48)	0.93(0.50-1.73)	0.83
No	182	92	1	1	

1, reference category

Table 6:-Associated factors of attitude towards COVID-19 prevention among adult visitors in hospitals of South Gondar zone, Northwest Ethiopia, in August 1 to 30, 2020

Variable	Attitude		COR (95% CI)	AOR(95%CI)	P-value
	Positive	Negative			
Age					
<20	12	12	1	1	
20-29	57	35	1.63(0.66-4.02)	0.42(0.12-1.46)	0.17
30-39	66	45	1.47(0.61-3.56)	0.51(0.14-1.82)	0.30
40-49	57	37	1.54(0.63-3.79)	0.94(0.28-3.21)	0.93
50-59	32	16	2.00(0.74-5.44)	1.10(0.28-4.27)	0.89
≥60	29	6	4.83(1.47-15.87)	1.65(0.33-8.42)	0.55
Religion					
Muslim	25	5	3.27(1.05-10.20)	2.18(0.50-9.58)	0.30
Orthodox	202	129	1.02(0.53-1.96)	1.49(0.64-3.48)	0.36
Protestant	26	17	1	1	
**Education**					
cannot read and write	22	44	1	1	
Read and write	47	48	1.96(1.02-3.76)	2.39(0.99-5.79)	.053
Primary	27	9	6.00(2.41-14.93)	**6.49(1.52-27.78)***	**0.012***
secondary	21	8	5.25(2.01-13.74)	2.32(0.39-13.74)	.35
College and above	136	42	6.48(3.49-12.01)	**6.91(2.58-14.50)***	**0.0001***
Occupation					
Farmer	22	46	1	1	
Student	36	9	8.36(3.44-20.36)	1.87(0.33-10.72)	0.48
Currently unemployed	33	22	3.14(1.50-6.58)	0.54(0.18-1.68)	0.29
Gov't worker	105	24	9.15(4.66-17.96)	0.61(0.12-3.05)	0.55
Private business	57	50	2.38(1.26-4.50)	0.29(0.07-1.12)	0.07
Resident					
Urban	193	94	1.95(1.26-3.02)	1.23(0.66-2.23)	0.51
Rural	60	57	1	1	
Monthly income					
<499	72	55	1	1	
500-2000	61	40	1.17(0.69-1.98)	0.89(0.33-2.38)	0.82
>2000	120	56	1.64(1.02-2.63)	0.57(0.19-1.70)	0.31
History of chronic illness					
Yes	48	8	4.19(1.92-9.12)	**5.00(1.71-14.67)***	**0.003***
No	205	143	1	1	
Training					
Yes	113	24	4.27(2.59-7.05)	**3.9(1.96-7.70)***	**0.0001***

No	140	127	1	1	
Use social media					
Yes	179	73	2.59(1.70-3.93)	1.20(0.59-2.44)	0.63
No	74	78	1	1	
Peer					
Yes	231	114	3.41(1.92-60.5)	**2.45(1.06-5.63)***	**0.04***
No	22	37	1	1	
Tv/radio					
Yes	221	100	3.52(2.13-5.81)	2.091(0.85-5.16)	0.11
No	32	51	1	1	
Religious institution					
Yes	100	30	2.64(1.64-4.23)	1.725(0.93-3.21)	0.09
No	153	121	1	1	

1, reference category

Associated factors of COVID-19 preventive practice

Table 7: Associated factors of preventive practice towards COVID-19 prevention among adult visitors in hospitals of South Gondar zone, Northwest Ethiopia, in August 1 to 30, 2020

Variable	Practice status		COR (95% CI)	AOR (95% CI)	P-value
	Good	Poor			
**Age**					
<20	9	15	1	1	
20-29	48	44	1.82(0.72-4.57)	0.87(0.24-3.38)	0.84
30-39	49	62	1.32(0.53-3.26)	0.43(0.12-1.67)	0.22
40-49	41	53	1.29(0.51-3.24)	0.75(0.20-2.56)	0.67
50-59	27	21	2.14(0.79-5.85)	0.99(0.23-4.24)	0.98
≥60	25	10	4.17(1.38-12.58)	1.31(0.28-6.11)	0.73
**Education**					
cannot read and write	17	49	1	1	
Read and write	25	70	1.03(0.50-2.11)	0.93(0.36-2.43)	0.88
Primary	16	20	2.31(0.98-5.44)	0.39(0.08-1.80)	0.23
Secondary	17	12	4.08(1.62-10.27)	0.82(0.16-4.19)	0.82
College and above	124	54	6.62(3.50-12.52)	1.90(0.67-5.17)	0.21
**Occupation**					
Farmer	6	62	1	1	
Student	24	21	11.81(4.25-32.83)	**7.70(1.15-15.86)***	**0.04***
Currently unemployed	20	35	5.91(2.17-16.08)	2.35(0.58-9.57)	0.23
Gov't worker	93	36	26.70(10.62-67.12)	2.49(0.42-14.61)	0.31
Private business	56	51	11.35(4.52-28.47)	2.15(0.45-10.2)	0.34
**Resident**					
Urban	166	121	3.49(2.19-5.56)	1.54(0.79-3.00)	0.21
Rural	33	84	1	1	
**Monthly income**					
<499	39	88	1	1	
500-2000	50	51	2.21(1.29-3.81)	2.05(0.71-5.93)	0.19
>2000	110	66	3.76(2.32-6.12)	1.99(0.62-6.39)	0.25
**Training**					
Yes	86	51	2.30(1.51-3.51)	0.88(0.47-1.64)	0.68
No	113	154	1	1	
**Use social media**					
Yes	160	92	5.04(3.23-7.87)	1.54(0.76-3.10)	0.23
No	39	113	1	1	
**Peer**					
Yes	184	161	3.35(1.80-6.25)	0.78(0.31-1.97)	0.61
No	15	44	1	1	
**Tv/radio**					
Yes	185	136	6.70(3.62-12.41)	1.45(0.53-3.96)	0.46
No	14	69	1	1	
**Knowledge**					

Poor knowledge	22	102	1	1	
Good knowledge	177	103	7.97(4.73-13.41)	**4.49(2.41-8.39)***	**0.0001***
**attitude**					
Negative attitude	44	107	1	1	
Positive attitude	155	98	3.85(2.50-5.93)	1.04(0.58-1.86)	0.068

1, reference category

Click here to access/download
Supporting Information
SI I English Version of the Questionnaire.docx

Click here to access/download

Supporting Information

SI II Amharic Version of the Questionnaire.docx

Click here to access/download
Supporting Information
SI III. Data set.xlsx

Knowledge, Attitude, and Practices towards COVID-19 and associated factors among adult hospital Visitors in South Gondar Zone Hospitals, Northwest Ethiopia

Gete Berihun[1*,#], Zebader Walle Belete[2#], Awoke Keleb[1], Ayechew Ademas[1], Leykun Berhanu[1], Masresha Abebe[1], Adinew Gizeyatu[1], Seada Hassen[1], Daniel Teshome[3], Mistir Lingerew[2], Alelgne Feleke[2], Tarikuwa Natnael[2], Metadel Adane[1]

[1]Department of Environmental Health, College of Medicine and Health Sciences, Wollo University, Dessie, Ethiopia.

[2]Department of Public Health, College of Health Sciences, Debre Tabor University, Debre Tabor, Ethiopia.

[3]Department of Anatomy, College of Medicine and Health Sciences, Wollo University, Dessie, Ethiopia.

[*] **Corresponding author: geteberihun@gmail.com**

#Those authors contributed equally to this work

Abstract

Background

Coronavirus disease 2019 is currently the critical health problem of the globe, including Ethiopia. Visitors of healthcare facilities are the high-risk groups due to the presence of suspected and confirmed cases of coronavirus 2019 in healthcare setting. Increasing the knowledge, attitude and practices towards COVID-19 prevention among hospitals visitors are very important to prevent transmissions of the pandemic despite lack of evidence remains a challenge in Ethiopia. Therefore, this study was designed to investigate the status of knowledge, attitude, and practice towards COVID-19 and associated factors among hospital visitors in South Gondar Zone Hospitals, Northwest Ethiopia.

Methods

A facility-based cross-sectional study design was employed ~~during~~ from August 1 to 30, 2020 from randomly selected 404 hospital visitors in South Gondar Zone Hospitals, northwest Ethiopia. Data was collected using interviewer administered questionnaire. The outcome of this study was good or poor knowledge, positive or negative attitude and good or poor practice towards COVID-19. A binary logistic regression model with 95% CI (Confidence interval) was used for data analysis. Bivariable analysis with (COR [crude odds ratio]) and multivariable analysis (AOR [adjusted odds ratio]) was used during data analysis. From the bivariable analysis, variables with a *p*-value <0.25 were retained into the multivariable logistic regression analysis. From the multivariable logistic regression analysis, variables with a significance level of *p*-value

<0.05 were taken as factors independently associated with knowledge, attitude and practices towards COVID-19.

Main findings

About 69.3% of the respondents had good knowledge, 62.6% had positive attitude, and 49.3% had good practice towards the prevention of COVID-19. We found that factors significantly associated with good knowledge about COVID-19 were educational status who can read and write (AOR=2.78; 95%CI: 1.18, 6.56) and college and above (AOR=6.15; 95%CI: 2.18-17.40), and use of social media (AOR=2.96; 95%CI: 1.46, 6.01). Furthermore, factors significantly associated with positive attitude towards COVID-19 includes presence of chronic illnesses (AOR=5.00; 95%CI; 1.71-14.67), training on COVID-19 (AOR=3.91; 95%CI: 1.96-7.70), and peer/family as a source of information (AOR=2.45; 95%CI: 1.06-5.63). Being a student (AOR=7.70; 95%CI: 1.15-15.86) and participants who had a good knowledge on COVID-19 (AOR=4.49; 95%CI: 2.41-8.39) were factors significantly associated with good practice towards COVID-19.

Conclusion

We conclude that the status of knowledge, attitude and practice towards COVID-19 prevention was not satisfactory. Factors significantly associated with good knowledge were educational status who can read and write and college and above and use of social media. Factors significantly associated with positive attitude include presence of chronic illnesses, training on COVID-19, and peer/family as a source of information. Being a student and participants who had a good knowledge were factors significantly associated with good practice towards COVID-19. Hence, intervention strategies such as health education and infection prevention and control that

could improve the knowledge, attitude and practice status towards COVID-19 preventions are urgently needed to control the transmission of COVID-19.

Keywords: Knowledge, attitude, practice, associated factors, COVID-19, Ethiopia

Introduction

Corona virus 2019 (COVID-19) is a rapidly emerging pandemic respiratory disease caused by a novel Coronavirus of Severe Acute Respiratory Syndrome (SARS/COV-2). The disease was reported initially in Wuhan city, Hubei Province, China at the end of December 2019 (1–3). Later on, World Health Organization (WHO) announces the disease as a public health emergency of international concern at the end of January 2020 and then declared as a global pandemic on March 11 (4–6). Two days later, the government of Ethiopia reported the first confirmed case of COVID-19 (7,8).

COVID-19 transmits mainly through droplets, airborne transmission, and contact between humans (6,9–11). The major sign and symptoms of COVID-19 cases are fever, dry cough, fatigue, myalgia, shortness of breath, and dyspnoea (4–6). The Severe cases of the disease may lead to the developments of cardiac injury, respiratory failure, acute respiratory distress syndrome, and death. Elders and patients with chronic medical illnesses like hypertension, cardiac disease, lung disease, cancer, or diabetes have been identified as potential risk factors for disease severity and mortality (6,11).

According to the Worldometer report, as of October 6, 2020, 9:54 am, COVID-19 spreads to more than 214 countries across the world. Worldwide, a total of 35,707,844 confirmed cases were reported. Of them, 26,907,997 were recovered and 1,049,700 were died of the pandemic

(12). In case of Ethiopia, 79,437 confirmed cases of COVID-19 were reported. Of this, 1,230 and 34,016 were died and recovered, respectively (13).

Due to the absence of cure (6,11) prevention is recommended as the only strategy to prevent the spread of COVID-19. Different COVID-19 prevention measures are implemented such as respiratory hygiene, hand washing, social distancing, use of Personal Protective Equipment (PPE), and environmental disinfection (6,15–18). WHO designed different guidelines and online training sessions to increase the awareness of the community towards the prevention of the pandemic (19).But still, there is a deficiency of information mainly for vulnerable groups (6,18).

The government of Ethiopia has also implemented different prevention measures. Later on, the country declares and enforces a state of emergency for about six months since March 2020 But most populations of Ethiopia perceived that as the disease was eliminated since the termination of a state of emergency. Therefore, prevention measures towards COVID-19 are becoming neglected from time to time. Healthcare facilities are one the vulnerable area for transmission of COVID-19.As a result, visitors of healthcare facilities are one of the victim groups of population for COVID-19 due to close contact with suspected and confirmed cases of the disease. Even though many researchers were conducted on KAP towards prevention of COVID-19, there is limited evidence on KAP of healthcare facility visitors. Therefore, assessing knowledge, attitude, and practice are important measures for identifying gaps and taking of intervention accordingly. (4). Therefore, the study was designed to assess knowledge, attitude, and practice of adult visitors towards COVID-19 Prevention in South Gondar Zone Hospitals, Northwest Ethiopia.

Methods and materials

Study design, period, and setting

A ~~health~~ facility-based cross-sectional study design was applied among hospital visitors in South Gondar Zone Hospitals, Northwest Ethiopia ~~during~~ August 1 to 30, 2020. South Gondar zone is one of the 13 administrative zones in the Amhara regional state of Ethiopia. Debre Tabor is its capital town which is located at 597 km and 105 km from Addis Ababa and Bahir Dar, respectively. According to the population projection of 2014, the total population of the study area was 2,364,603 of which 1,196,318 were males while 1,168,285 were females (population projection 2014).

In South Gondar, there are one general hospital found in Debre Tabor Town and seven district government hospitals found in Addis Zemen, Mekane Eyesus, Andabet, Ebnat, Lay Gayint, Tach Gayint, and Smada towns. In addition, there are 98 government health centers, and 76 private clinics in South Gondar Zone (23). According to the reports of hospitals in South Gondar Zone, the average monthly visitors during COVID-19 were 13,440.

Source and study population

All hospital visitors whose age 18 years and above in South Gondar Zone Hospitals were the source population, whereas the study populations were those randomly selected study participants from the two randomly selected hospitals (Debre Tabor and Mekane Eyesus). Those study participants whose age below 18 years were excluded.

Sample size estimation and sampling methods

The sample size was determined using the single population proportion formula by taking the following assumptions.

$$n = \frac{(z_{\alpha/2})^2 * p(1-p)}{d^2}$$

$Z_{\alpha/2}$ is the standard normal variable value at (1-α)% confidence level (α is 0.05 with 95% CI, $Z_{\alpha/2}$ = 1.96), an estimate of the proportion of knowledge attitude and practice, was considered as 50% as there was no similar studies conducted and 5% margin of error was considered. The sample size became 384 and after considering of 10% non-response rates, the adequate sample final sample size becomes 422.

After selecting the two hospitals randomly out of the 8 hospitals, we proportionally allocated sample size based on total estimated visitors of hospitals in the last three months. Then, 303 sample size was allocated for Debre Tabor general hospitals and 117 for Mekane Eyesus hospitals. Then hospitals visitors flow data during the previous 3 months in emergency ward, surgical ward, medical ward, gynecology/obstetrics ward and pediatrics ward considered for sample size allocation for each hospitals departments. Finally, randomly selection of visitors for each ward was selected until the allocated sample size achieved.

Outcome variable measurement

The outcome variable was good or poor knowledge, positive or negative attitude and good or poor practice towards COVID-19.

Good or poor knowledge: Knowledge was measured by using 15 questions consisting of signs and symptoms, risk groups and prognosis, method of transmission, and /preventive methods. Each question was consisted of as 'Yes', 'No', and 'I do not know' options. Respondents who answered correctly were given 1 point while others were given 0 points. The total knowledge score ranges from 0-15 and a cut-off level of ≥12 (80% and above) was considered as good knowledge while <12 (80%) was considered as poor knowledge towards COVID-19 prevention (24).

Positive or negative attitude: Attitude was measured by using 11 items and the response was categorized based on 3 scale measurements with agree (3 points), neutral (2 points), and disagree (1 point). The score of attitude varied from 11 to 33, with an overall score of ≥ 27 (81.8%) was considered as a positive attitude, whereas a score of less than 27 (81.8%) was considered as negative attitude towards COVID-19 prevention (27).

Good or poor practice: The practice was measured using 10 items and those who respond as yes were given 1 point while no was marked as 0. The total prevention practice score ranges from 0-10 and a score with a cut-off ≥ 8 (80%) was considered as good practice while <8 was taken as a poor practice (25,26).

Data collection and quality assurance

Data were collected using a pre-tested structured questionnaire which was adapted from published articles in reputable journals and from WHO COVID-19 guidelines (28–33). The questionnaire consists of five sections including; part I: socio-demographic characteristics of the participants; part II; Pre-existing medical condition and sources of information towards COVID-19; part III: knowledge of the participants; part IV: Attitude of the participants; and part V:

Prevention practice of COVID-19. The tool was prepared in the English version and translated to Amharic version (local language), and re-translated back to English to ensure consistency. The tool was pre-tested using 5% of the final sample size in Andabet district hospital visitors to establish the validity of the questionnaire. Based on the pre-test, appropriate amendments such as order arrangement of questions, editing of unclear questions, and avoiding irrelevant questions were done accordingly.

The data was collected using interviewer administered method using four BSc nurse professionals and supervised by two Public Health experts. Two days of training was given for data collectors and supervisors on the overall aim of the study, contents of the tool and ethical issues. Supervision was carried out on daily basis, and appropriate corrections of the collected data were done accordingly. Furthermore, data entry was re-checked for 10% of the sample size in order to control data entry errors of the entered data and data cleaning was carried before statistical analysis. The reliability coefficient of Cronbach's alpha was 0.76 which is an acceptable range.

Statistical analysis

Data was entered into EpiData version 4.6 and exported to the Statistical Package of the Social Science (SPSS) version 25.0 for data cleaning analysis. Descriptive statistic such as frequencies and percentages were calculated for categorical variables and mean with standard deviations for continuous variables to examine the overall distribution.

Associations between independent variables and knowledge, attitudes and practices towards COVID-19 were determined using ~~a binary~~ logistic regression model at 95% CI (Confidence interval). We used three d~~ifferent logistic regressi~~on models: The first model (Model 1)

identified factors associated with good knowledge about COVID-19, the second model (Model II) identified factors associated with favorable attitudes and the third model (Model III) identified factors associated factors with good preventive practices towards COVID-19. For each model, bivariable analysis with (COR [crude odds ratio]) and multivariable analysis (AOR [adjusted odds ratio]) was used.

From the bivariable analysis, variables with a *p*-value <0.25 were retained into the multivariable logistic regression analysis. From the multivariable analysis of each model, variables with a significance level of *p*-value <0.05 were taken as factors independently associated with knowledge, attitude and practices towards COVID-19. The presence of multicollinearity among independent variables was checked using standard error at the cutoff value of 2 and we found that a maximum standard error of 0.97, which indicated no multi-collinearity. Model fitness was checked using the Hosmer-Lemeshow test for Model I, Model II and Model III and found a *p*-value of 0.650, 0.871 and 0.913, respectively and indicated that all models were fit.

Ethics approval and consent to participate

The study was approved by the ethical review committee of Debre Tabor University. Permission to conduct the study was obtained from the respective hospital managers of the study site. Before the data collection, the purpose of the study was explained and verbal consent was obtained from each participant. Individuals who were volunteer to participate in the study were also told as they have the right to withdraw from the study at any stage of the interview. The confidentiality of the study participants was ensured by avoiding possible identifiers. Data collectors wear a facemask and keep a physical distancing of two feet. Facemask was provided for the study participants who did not wear during the data collection.

Result

Socio-demographic characteristics of hospital visitors

A total of 404 visitors participated in the study with a response rate of 95.7%. Nearly two-thirds 242 (59.7%) of the study participants were females and about one-fifth 92 (22.8%) of the the hospital visitors were in the age range of 30-39 years. Furthermore, 66 (16.3%), 68(16.8%), and 117(29.0%) of the respondents can't read and write, farmers, and live in rural areas respectively. Finally, 56 (13.9%) of the participants had either or more chronic medical illness history (Table 1).

Knowledge of hospital visitors towards COVID-19 prevention

More than two-thirds 280 (69.3%; 95%CI; 65.1-73.8%) of the visitors had a good knowledge, whereas 124 (30.7%; 95%CI: 27.2-34.9%) of them had poor knowledge about COVID-19. Almost all 388 (96.0%) of the participants heard about COVID-19 and more than three-fourths 322 (79.7%) of the participants knew as COVID-19 is a viral disease and 339 (83.9%) of them knew the major sign and symptoms of COVID-19 cases. Furthermore, more than three-fourth 320 (79.2%) of the participants knew that elders, those who had a chronic medical illness, and being obese are more likely to have severe cases of COVID-19. Similarly, 283 (70.0%) of the respondents knew that COVID-19 can be transmitted from one person to another even in the absence of COVID-19 (Table 2).

Attitude of hospital visitors towards COVID-19 prevention

About two-thirds 62.6% (95%CI;57.2-67.6%) of the hospital visitors had a positive attitude towards COVID-19 prevention, whereas 37.4% (95%CI: 32.4-42.8%) had negative attitude

towards COVID-19 prevention. About half 203 (50.2%) of the participants agree that the black race is not protective against COVID-19. Similarly, less than half 180 (44.6%) of the participants agreed that Ethiopia is in a good position to contain the spread of the COVID-19. About two-thirds 274 (67.8%) of the participants believed COVID-19 do not cause stigma. More than half 221(54.7%) of the respondents agree that they can get infected with COVID-19 if they contacted infected patients despite their good immunity. On the other hand, 55 (13.6%) of the respondents believed that COVID-19 has occurred as a result of our sin (Table 3).

Preventive practice of visitors towards COVID-19 prevention

Half of the respondents 199 (49.3%) practiced the recommended COVID-19 prevention methods. Majority 378 (93.6%) of the participants washed their hands with water and soap for at least 20 seconds. Furthermore, almost nine out of ten respondents avoid handshaking practice for prevention of COVID-19. But a relatively lower number of 338 (83.7%) participants used facemasks when they leave their home and 333 (82.4%) practiced respiratory hygiene while coughing and sneezing. Furthermore less than half 177(43.8%) participants applied keeping of the recommended physical distance for prevention of COVID-19. Staying at home was also another challenge and only less than one-third 121(30%) of the participants applied it (Table 4).

Factors Associated with Knowledge, Attitude, and practice towards COVID-19 from multivariable analysis

A multi-variable analysis from the first model indicated that educational status and use of social media as a source of information were statistically significant with the knowledge of COVID-19. The fining revealed that those who can read and write were 2.78 times more likely to have good knowledge towards COVID-19 prevention methods than those who could not read and write. Similarly, participants who have college and above educational level were 6.15 (AOR = 6.15; 95%CI: 2.18-17.40) times more likely to have good knowledge than those who could not read and write. Furthermore, participants who used social media as a source of information towards COVID-19 was 2.96 (AOR = 2.96; 95%CI: 1.46-6.01) times more likely to have good knowledge than those who did not use social media (Table 5).

A multi-variable analysis from the second model revealed that those who had primary education were 6.49 (AOR = 6.49; 95%CI: 1.52-27.78) times more likely to have a positive attitude than those who could not read and write while being college and above graduated were 6.91 (AOR = 6.91; 95%CI: 2.58-14.5) times more likely to have a positive attitude than the corresponding reference group. Furthermore, visitors who had chronic medical illnesses were 5 times (AOR = 5; 95%CI: 1.71-14.67) more likely to have a positive attitude than those who did not have a chronic illness. Furthermore, participants who took training on COVID-19 prevention were 3.9 (AOR = 3.9; 95%CI: 1.96-7.70) times more likely to have a positive attitude than those who didn't take the training. Additionally, participants who used peer as a source of information towards COVID-19 prevention were 2.45 (AOR = 2.45; 1.06-5.63) times more likely to have a

positive attitude than those who didn’t use peers as a source of information for COVID-19 prevention (Table 6).

From multivariable analysis of model three, we found that being a student was 7.7 times (AOR = 7.7; 95%CI: 1.15-51.86) more likely to have a good practices than farmers. Furthermore, participants who had good knowledge were 4.49 (AOR = 4.49; 95%CI: 2.41-8.39) times more likely to have a good practice about COVID-19 prevention than those who poor knowledge (Table 7).

Discussion

The pandemic of COVID-19 is still the critical concern of the globe including our country Ethiopia. But up to date, there is no confirmed treatment for the pandemic. Therefore prevention is the single most important method of alleviating the spread of COVID-19 pandemic.

In this finding, about 81.67% of the knowledge questions were correctly replied to by the respondents. This finding was in line with the study conducted in Saudi Arabia (80.5%) (4) and in Nigeria (77.36) (35). The finding of this study was lower than the study conducted in China (90%) (36). This discrepancy may be due to variation in the study population's characteristics, government commitment, and health care system. On the contrary, this study result was higher than in the Egyptian population (71.26%) (37). This discrepancy might be due to the variation in socio-demographic characteristics of the population.

The finding of this study revealed that 69.3 % (CI; 65.1-73.8) of the participants had good knowledge towards COVID-19 prevention which was in line with the study conducted in India (70 %) (38). On the other hand, this study finding was lower than a multicenter study conducted among health care workers in Ethiopia with 88.2% (16) and Nigerian residents in an urban

setting (99.7%) (39). This deviation may be due to variations in socio-demographic characteristics of the study population and sources of information towards COVID-19.

This study also revealed that about 80% of participants knew that the elderly, those who had chronic medical illnesses, and obese are more likely to develop severe cases of COVID-19. This finding was slightly higher than the study conducted in Ethiopia (72.5%) (11**).** This variation may be due to the change in study period, socio-demographic characteristics of the study population, and coverage of awareness creation towards COVID-19 prevention. Even though children and young adults are vulnerable groups, only 83.4% of the participants knew that these groups need to take preventive measures towards COVID-19. Neglecting such types of the population may wide-spreading the transmission of the pandemic (11).

Regarding the attitudes, 62.6% (95% CI; 57.2-67.6) of respondents had a positive attitude towards COVID-19 prevention which was lower than the study conducted in Ethiopia (94.7%) (16), Nigeria 79.5% (39), and Pakistan (82.16%) (40). This discrepancy may be due to a change in the socio-demographic characteristics of the study population, government commitment towards COVID-19. On the other hand, less than half (44.6%) of the participants believed that the government of Ethiopia can control the spread of COVID-19 within a short time. This finding was lower than the study conducted in china 97.1% (24) and India at 87.2% (41). This deviation may be due to the variation in the quality of the health care system, socio-demographic characteristics of the study population, and government preparedness towards the control of COVID-19 pandemic. According to the WHO report, the government of Ethiopia scored 52% towards COVID-19 preparedness response (21) which supports the finding of this study. Furthermore, this study also indicated that almost two-thirds of the respondents believed that the pandemic of COVID-19 leads to the development of social stigma which was lower than a study

conducted in Ethiopia at 77% (16) and 83.8% (11). This deviation may be due to differences in socio-demographic characteristics of the study population and study period. On the contrary, this study finding was higher than the study conducted in the Peruvian population 59.1% (42). This variation may be due to a change in the socio-demographic characteristics of the study population, study period, awareness creation towards COVID-19, and the burden of the pandemic. The social stigma may be developed due to fear of its mortality and high communicability. The history of social stigma due to pandemic was not a new phenomenon (43,44).

Regarding the prevention practice of COVID-19, the overall practice score of the respondents was 73.2% which was higher than the study conducted in Ethiopia (26). The finding of this study revealed that only half of the respondents 49.3% had a positive preventive practice of COVID-19. The finding of this study was lower than other studies conducted in Ethiopia (16,26) and China (45). This variation may be due to the change in the study setting, socio-demographic characteristics of the study population, and occupation of the study participant (being a health professional vs. general population), and the commitment of the government towards the prevention of COVID-19. Furthermore, most of the participants 93.6% washed their hands with water and soap for at least 20 seconds which was in line with a study conducted in Nigeria 96.4% (39). On the contrary, this finding was lower than a study conducted in Nigeria 87.9 % (35). This deviation may be to a change in access and utilization of handwashing facilities in health care facilities.

Furthermore, 83.7% of the participants used face masks for prevention of COVID-19 which were consistent with the study conducted in Nigeria 84.4% (35), and 82.3% (39). This finding also revealed that less than half (43.8%) of the respondents applied the recommended physical

distance of 2 meter when they go to public crowded areas. This finding was lower than the study conducted in Nigeria 83% (35) and 92.7%, (39). This variation may be due to a change in the socio-demographic characteristic of the study population, the burden of the disease, awareness of the community towards the COVID-19 pandemic, and population way of life.

The finding also revealed that more than two-thirds 70% of the respondents avoid going to crowded places after the emergence of COVID-19 which was higher than the finding in Nigeria 58.9% (35). in addition to this, 82.4% of the respondents practiced respiratory hygiene which was lower than the finding in India (97.7%) (46). The variation might be due to a change in a study setting, heterogeneity of population perception of the community, knowledge towards COVID-19, and burden of confirmed COVID-19 cases. Above all, the most common problem which was not applied by the participants was staying at home and only less than one-third (30%) after the occurrence of COVID-19. The possible justification for this might be due to the uncontrolled of the pandemic for a long period of time, poor preparedness of the community towards the pandemic, and subsistence way of life of the community.

Conclusion

We conclude that the status of knowledge, attitude and practice towards COVID-19 prevention was not satisfactory. Factors significantly associated with good knowledge about COVID-19 were educational status who can read and write and college and above and use of social media. Factors significantly associated with positive attitude towards COVID-19 include presence of chronic illnesses, training on COVID-19, and peer/family as a source of information. Being a student and participants who had a good knowledge on COVID-19 were factors significantly associated with good practice towards COVID-19. Hence, intervention strategies that could

improve the knowledge, attitude and practice status towards COVID-19 preventions are urgently needed to control the transmission of COVID-19. Health education about COVID-19 infection prevention and control methods and advocacy about COVOD-19 recommended prevention using various media channels are highly recommended. Therefore, hospital administrators should work in collaboration with other concerned stakeholders to enhance knowledge, attitude, and practice of hospital visitors to wards COVID-19 prevention.

Abbreviations

COVID-19: Coronavirus disease 2019; AOR: Adjusted Odds Ratio; CI: Confidence Interval; COR: Crude Odds Ratio; PPE: Personal Protective Equipment; SARAS_COV-1: Severe Acute Respiratory Syndrome- Corona Virus-1; SARS-COV-2: Severe Acute Respiratory Syndrome-Corona Virus-2

Acknowledgments

Our heartfelt gratitude extends to Debre Tabor University for providing ethical clearance for this study. Debra Tabor and Mebane Eyesus hospital administrators are duly acknowledged for their permission to conduct this study and for the information we received while we needed. We also thank the study participants who gave valuable information and their kind cooperation during this study. We are also grateful for data collectors and supervisors for their commitment during the data collection.

References

1. Elhadi M, Msherghi A, Alkeelani M, Zorgani A, Zaid A, Alsuyihili A, et al. Assessment of healthcare workers' levels of preparedness and awareness regarding Covid-19 infection in low-resource settings. Am J Trop Med Hyg. 2020;828–33.

2. WHO Regional Office for Africa. COVID-19 Situation Update for WHO African Region, External Situation Report. 2020 Jun. Report No.: 15.

3. Saqlain M, Munir MM, Ur Rehman S, Gulzar A, Naz S, Ahmed Z, et al. Knowledge, attitude, practice and perceived barriers among healthcare professionals regarding COVID-19: A Cross-sectional survey from Pakistan. Occupational and Environmental Health; 2020 Apr.

4. Al-Hanawi MK, Angawi K, Alshareef N, Qattan AMN, Helmy HZ, Abudawood Y, et al. Knowledge, Attitude and Practice Toward COVID-19 Among the Public in the Kingdom of Saudi Arabia: A Cross-Sectional Study. Front Public Heal. 2020;8(May).

5. Iannone P, Castellini G, Coclite D, Napoletano A, Fauci AJ, Iacorossi L, et al. The need for health policy perspective to protect Healthcare Workers during COVID-19 pandemic. A GRADE rapid review on the effectiveness of the N95 respirator. PLoS One [Internet]. 2020;15(6):1–13. Available from: http://dx.doi.org/10.1371/journal.pone.0234025

6. Nallani VRR, Nadendla RR, Kavuri NSS. Knowledge, attitude, and practice among health care professionals regarding COVID-19 and barriers faced by health care professionals inSouth India. Int J Community Med Public Heal. 2020;7(9):3450.

7. Lone SA, Ahmad A. COVID-19 pandemic – an African perspective. :10.

8. Baye K. COVID-19 prevention measures in Ethiopia Current realities and prospects. The Federal Democratic Republic of Ethiopia Policy Studies Institute; 2020.

9. Ssebuufu R, Katembo Sikakulya F, Binezero SM, Wasingya L, Nganza SK, Ibrahim B, et al. Awareness knowledge attitude and practice towards measures for prevention of the spread of COVID-19 in the Ugandans A nationwide online cross-sectional Survey. Medrxiv. 2020;1–28.

10. WHO. COVID-19 Strategic Preparedness and Response Plan Operational Planning Guideline to Support Country Preparedness and Response. 2020.

11. Kebede Y, Yitayih Y, Birhanu Z, Mekonen S, Ambelu A. Knowledge, perceptions and preventive practices towards COVID-19 early in the outbreak among Jimma university medical center visitors,Southwest Ethiopia. PLoS One [Internet]. 2020;15(5):1–15. Available from: http://dx.doi.org/10.1371/journal.pone.0233744

12. Worldometer Report COVID-19 coronavirus pandemic 2020. https://www.worldometers.info/coronavirus, 2020 accessed date October 6, 2020, 9.54 am

13. Worldometer report.COVID 19 coronavirus pandemic 2020. https://www.worldometers.info/coronavirus/country/ethiopia,2020 accessed date October 6, 2020, 9.54 am

14. Nigussie T.F and Azimach N.N. Knowledge, Attitude and Practice towards COVID-19 among Arba Minch Town GSJ : Volume 8, Issue 6, June 2020, Online : ISSN 2320-9186

15. CDC. Infection prevention and control for COVID-19 in healthcare settings - first update 12 March 2020. Eur Cent Dis Prev Control. 2020;(March):1–10.

16. Jemal B, Ferede ZA, Mola S, Hailu S, Abiy S, Wolde GD, et al. Knowledge, attitude, and practice of healthcare workers towards COVID-19 and its prevention in Ethiopia: a multicenter study. In Review; 2020 May.

17. Olum R, Chekwech G, Wekha G, Nassozi DR, Bongomin F. Coronavirus Disease-2019: Knowledge, Attitude, and Practices of Health Care Workers at Makerere University Teaching Hospitals, Uganda. Front Public Heal. 2020 Apr;8:181.

18. Lakshmi Priyadarsini S, Suresh M. Factors influencing the epidemiological characteristics of pandemic COVID 19: A TISM approach. Int J Healthc Manag. 2020;13(2):89–98.

19. Saqlain M, Munir MM, Rehman SU, ... Knowledge, attitude, practice and perceived barriers among healthcare professionals regarding COVID-19: A Cross-sectional survey from Pakistan. J … [Internet]. 2020; Available from https://www.ncbi.nlm.nih.gov/pmc/articles/PMC7211584/

20. Goshu D, Ketema M. Assessment of COVID-19 Effects and Response Measures in Ethiopia : Livelihoods and Welfare Implications Assessment of COVID-19 Effects and Response Measures in Ethiopia : Livelihoods and Welfare Implications 1. 2020.

21. Tolu LB, Ezeh A, Feyissa GT. How Prepared Is Africa for the COVID-19 Pandemic Response ? The Case of Ethiopia What Measures Should Be Taken to. 2020;

22. Alemayehu TY, Boled AT, Taye MG, Kibret S. Assessment of emergency and essential surgical service in primary hospitals ofSouth Gondar zone, Amhara region, Ethiopia, 2019 International Journal of Surgery Open Assessment of emergency and essential surgical service in primary hospitals ofSouth Gondar zone, Amhara region, Ethiopia, 2019. Int J Surg Open [Internet]. 2020;23(February):35–8. Available from: https://doi.org/10.1016/j.ijso.2020.02.003

23. Abate BA, Ahunie MA. Basic Emergency Maternal And Neonatal Care Status OfSouth Gon- Dar Zone, North Central Ethiopia: Institutional Descriptive Sur- Vey, June 2016. 2017;9(1):11.

24. Giao H, Han NTN, Khanh T Van, Ngan VK. Knowledge and attitude toward COVID-19 among healthcare workers at District 2 Hospital, Ho Chi Minh City. Asian Pac J Trop Med. :7.

25. Defar A, Molla G, Abdella S, Tessema M, Ahmed M, Tadele A et. al. Knowledge, Practice and associated factors towards the Prevention of COVID-19 among high-risk groups: A cross-sectional study in Addis Ababa, Ethiopia. 2020;19.

26. Asemahagn MA. Factors determining the knowledge and prevention practice of healthcare workers towards COVID-19 in the Amhara region, Ethiopia : a cross-sectional survey. 2020;3.

27. Salman M, Mustafa ZU, Asif N, Zaidi HA, Shehzadi N. Knowledge, attitude and preventive practices related to COVID-19 among health professionals of Punjab province of Pakistan.

28. WHO E. Survey Tool And Guidance Rapid, simple, flexible behavioral insights on COVID-19. 2020.

29. Ahmad NA, Ahmad W, Arman R, Rahimi W, Ahmadi A, Vijay Raghavan SMS. Community Perception Survey – COVID 19 Knowledge, Attitude, and Practice Survey in Kabul, Kunduz, and Khost Province. the JOHANNITER international assistance, JACK, OHW; 2020.

30. National Comprehensive Covid19 Management Handbook. :162.

31. National Institute of Environmental Health Science. COVID-19_BSSR_Research_Tools.pdf. 2020.

32. Admissions R, Homes R. COVID-19 : Infection Prevention and Control Checklist for Long-Term Care and Retirement Homes. 2020;1–15.

33. WHO. COVID-19 Infection Prevention and Control Sameeksha. 2020;2.

34. Ahmed N, Shakoor M, Vohra F, Abduljabbar T, Mariam Q, Rehman MA. Knowledge, Awareness, and Practice of Health care Professionals amid SARS-CoV-2, Corona Virus Disease Outbreak. :8.

35. Okoro J, Ekeroku A, Nweze B, Odionye T, Nkire J, Onuoha M, et al. Attitude and preventive practices towards COVID-19 disease and the impact of awareness training on knowledge of the disease among correctional officers [version 1 ; peer review : awaiting peer review]. 2020;1–10.

36. Zhong B, Luo W, Li H, Zhang Q, Liu X, Li W, et al. Knowledge, attitudes, and practices towards COVID-19 among Chinese residents during the rapid rise period of the COVID-19 outbreak : a quick online cross-sectional survey. 2020;16.

37. Abdelhafiz AS, Mohammed Z, Ibrahim ME, Ziady HH, Alorabi M, Ayyad M, et al. Knowledge, Perceptions, and Attitude of Egyptians Towards the Novel Coronavirus Disease (COVID-19). J Community Health [Internet]. 2020;45(5):881–90. Available from: https://doi.org/10.1007/s10900-020-00827-7

38. Singh JP, Sewda A. Assessing the Knowledge, Attitude, and Practices of Students Regarding the COVID-19 Pandemic. 2020;

39. Christopher R, Margaret R, Dauda MAD, Saleh A, Ene P. Knowledge, Attitudes and Practices Towards COVID - 19 : An Epidemiological Survey in North - Central Nigeria. J Community Health [Internet]. 2020; (0123456789). Available from: https://doi.org/10.1007/s10900-020-00881-1

40. Hussain I, Majeed A, Imran I, Ullah M, Hashmi FK, Saeed H, et al. Knowledge, Attitude, and Practices Toward COVID - 19 in Primary Healthcare Providers : A Cross-Sectional Study from Three Tertiary Care Hospitals of Peshawar, Pakistan. J Community Health [Internet]. 2020;(0123456789). Available from: https://doi.org/10.1007/s10900-020-00879-9

41. S DP, B KR, Athul K, Swamy S, Bhodaji S, Deshmukh A, et al. Knowledge, Attitude, Awareness and Practice Towards Covid-19 Pandemic in Indian Citizens During The National Lockdown Period : A Quick Online Cross-. 2020;8(May):3504–15.

42. Zegarra-Valdivia, J. A. 1; Chino-Vilca, B. N. 1; & Ames-Guerrero R. Knowledge, perception and attitudes regarding COVID-19 Pandemic in Peruvian Population.

43. Person B, Sy F, Holton K, Govert B, Liang A, Sars N. Fear, and Stigma : The Epidemic within the SARS Outbreak. 2004;10(2).

44. Paget J. ITALIAN OF PUBLIC HEALTH The influenza pandemic and Europe : the social impact and public health response OF PUBLIC. 2009;6(3):257–9.

45. Zhou M, Tang F, Wang Y, Nie H, Zhang L, You G, et al. Knowledge, attitude and practice regarding COVID-19 among health care workers in Henan, China. J Hosp Infect [Internet]. 2020; Available from https://doi.org/10.1016/j.jhin.2020.04.012

46. Awasthi AA, Taneja N, Janardhanan R. Knowledge, Attitude And Practice Towards Covid-19 Pandemic Among Residents Of Bihar, India. 2020;11(D):38990–5.

Table 1: Socio-demographic characteristics of adult visitors in hospitals of South Gondar zone Northwestern Ethiopia, August 1 to 30, 2020

Variable	Category	Frequency (*n*)	Percentage (%)
Sex	Male	163	40.3
	Female	241	59.7
Age(years)	<20	24	5.9
	20-29	92	22.8
	30-39	111	27.5
	40-49	94	23.3
	50-59	48	11.9
	≥60	35	8.7
Religion	Muslim	30	7.4
	Orthodox	331	81.9
	Protestant	43	10.6
Marital status	Single	83	20.5
	Married	295	73.1
	Divorced	26	6.5
Educational status	Cannot read and wright	66	16.3
	Read and write	95	23.5
	Primary (1-8) grade	36	8.9
	Secondary (9-12)grade	29	7.2
	College and above	178	44.1
Occupation	Farmer	68	16.8
	Student	45	11.1
	Unemployed	55	13.6
	Government employer	129	31.9
	Private business worker	107	26.5
Resident	Urban	287	71.0
	Rural	117	29.0
Monthly income(ETB)	≤499	127	31.4
	500-2000	101	25
	≥2001	176	43.6
History of chronic medical illness	Yes	56	13.9
	No	348	86.1
Training on COVID 19	Yes	137	33.9
	No	267	64.1
Use social media	Yes	252	62.4
	No	152	37.6
Peer as a source of information of COVID19	Yes	345	85.4
	No	59	14.6
Use TV/radio as a source of information	Yes	321	79.5
	No	83	20.5
Use religious institution as source of information	Yes	130	32.2
	No	274	67.8

Table 2: Knowledge of hospital visitors towards COVID-19 prevention in hospitals of South Gondar zone, Northwestern Ethiopia, August 1 to 30, 2020

**Item**	**Yes**		**No**		**I do not know**	
	**Frequency**	**Percent**	**Frequency**	**Percent**	**Frequency**	**Percent**
	n	**%**	**n**	**%**	***n***	**%**
Did you hear about COVID-19?	388	96.0	16	4.0		
COVID-19 is a viral disease.	322	79.7	40	9.9	42	10.4
The major sign and symptoms of COVID-19 are dry cough, fever, and shortness of breathing.	339	83.9	33	8.2	32	7.9
Runny nose and sneezing are less common symptoms of COVID-19.	275	68.1	88	21.8	41	10.1
Elder, those who have a chronic medical illness and obese are more likely to sever the case of COVID- 19.	320	79.2	57	14.1	27	6.7
Currently, there is no effective cure for COVID-19.	331	81.9	50	12.4	23	5.7
COVID-19 virus can spread via respiratory droplets.	375	92.8	29	7.2		
Eating and contacting wild animals would result COVID-19 infection	308	76.2	53	13.1	43	10.6
Persons with COVID 19 virus can transmit the virus to others when a fever is not present	283	70.0	71	17.6	50	12.4
Proper washing hand with soap and water is one method of preventing COVID-19.	375	92.8	18	4.5	11	2.7
Wearing general masks can prevent one from acquiring	354	87.6	35	8.7	15	3.7

infection by the COVID 19 virus						
Children and young adults must take measures to prevent the infection by Covid 19 virus	337	83.4	45	11.1	22	5.4
To prevent the infection by COVID 19 virus individuals should avoid going to crowded places such as bus parks and avoid public transportation	352	87.1	50	12.4	2	.5
People who have contact with someone infected with COVID 19 virus should be immediately isolated in a proper place in general the observation period is 14 days	273	67.6	95	23.5	36	8.9
Isolation and treatment of people who are infected with the COVID 19 virus are effective ways to reduce the spread of the virus	295	73.0	80	19.8	29	7.2

Mean± standard deviation = **12.25±2.45; Minimum = 2 and maximum = 15**

Table 3:-Attitude of adult visitors towards COVID-19 prevention in hospitals of South Gondar zone, Northwest Ethiopia, August 1 to 30, 2020

**Questions**	Agree	Neutral	Disagree
Black races are not protected from COVID 19 disease.	203(50.2%)	146(36.1%)	55(13.6%)
Wearing a well-fitting face mask are effective in preventing COVID 19 virus	268(66.3%)	81(20.0%)	55(13.6%)
Hand wash can prevent you from COVID 19 virus	321 79.5	77 19.1	6 1.5
Ethiopia is in a good position to contain COVID 19 virus	180 44.6	144 35.6	80 19.8
COVID 19 is not stigma and I should not hide my infection	274 67.8	90 22.3	40 9.9
If I get infected with COVID 19, I will go to the hospital as advised.	221 54.7	141 34.9	42 10.4
I can get infected with COVID 19 if I contacted an infected patient despite my good immunity.	230 56.9	100 24.8	74 18.3
COVID 19 is fatal	215 53.2	105 26.0	84 20.8
During the outbreak of COVID 19 eating well cooked and safely handled meat is safe.	249 61.6	96 23.8	59 14.6
COVID 19 patients should share their recent travel history with a health care provider.	256 63.4	85 21.0	63 15.6
Do you think that the cause of Covid-19 is not spiritual/ is it happened because of our sin?	262 64.9	87 21.5	55 13.6

***** Mean ±standard deviation = **27.11±4.08**; Minimum = **17**; Maximum = **33**

Table 4:-Preventive practice of adult visitors towards COVID-19 prevention in hospitals of South Gondar zone, Northwest Ethiopia, August 1 to 30, 2020

Questions	Yes		No	
	Frequency		Frequency	
	n	%	N	%
Do you avoid handshaking to prevent covid 19?	363	89.9	41	10.1
Have you washed your hands often with soap and water for at least 20 seconds especially after you have been in a public place or after blowing your nose, coughing, or sneezing?	378	93.6	26	6.4
If soap and water are not readily available, are you applying a hand sanitizer that contains at least 60% alcohol?	309	76.5	95	23.5
Do you wear face masks repeatedly when you leave your home?	338	83.7	66	16.3
Do you coughing and sneezing into the elbow or within clothing?	333	82.4	71	17.6
In recent days have you avoid going to any crowded place?	281	69.6	123	30.4
Do you avoid eating raw animal products to prevent the COVID 19 virus?	336	83.2	68	16.8
Do you avoid touching your mouth nose and eyes with unwashed hands?	323	80.0	81	20.0
Do you keep your self 2m away from the others when you got to the public area?	177	43.8	227	56.2
Do you stay at your home after the emergent of covid 19?	121	30.0	283	70.0
Mean ±standard deviation **7.32±1.60**				
Minimum **1.00**				
Maximum **10**				

Table 5:-Associated factors of knowledge towards COVID-19 prevention among adult visitors in hospitals of South Gondar zone, Northwest Ethiopia, August 1 to 30, 2020

**Variable**	**Knowledge status**		**COR(95% CI)**	**AOR(95% CI)**	**P-value**
	**Good**	**Poor**			
Age					
<20	12	12	1	1	
20-29	70	22	3.18(1.25-8.09)	1.98(0.54-7.29)	0.31
30-39	82	29	2.83(1.14-6.99)	1.08(0.30-3.87)	0.091
40-49	58	39	1.61(0.65-3.97)	1.06(0.31-3.68)	0.92
50-59	33	15	2.20(0.80-6.02)	1.21(0.30-4.82)	0.79
≥60	25	10	2.50(0.84-7.40)	0.69(0.16-2.95)	0.61
Marital status					
Single	61	22	1	1	
Married	205	90	0.82(0.48-1.42)	0.88(0.42-1.82)	0.72
Divorced	14	12	0.42(0.17-1.05)	0.92(0.29-2.98)	0.89
**Education**					
cannot read and write	25	41	1	1	
Read and write	54	41	2.16(1.14-4.12)	**2.78(1.18-6.56)***	**0.02***
Primary	26	10	4.26(1.76-10.31)	2.42(0.56-10.44)	0.24
Secondary	21	8	4.31(1.66-11.18)	1.54(0.25-9.56)	0.65
College and above	154	24	10.52(10.52-5.45)	**6.15(2.18-17.40)***	**0.001***
Occupation					
Farmer	23	45	1	1	
Student	33	12	5.38(2.35-12.34)	1.64(0.28-9.72)	0.59
Currently unemployed	38	17	4.37(2.04-9.36)	1.50(0.49-4.58)	0.48
Gov't worker	111	18	12.07(5.95-24.48)	0.83(0.16-4.19)	0.82
Private business	75	32	4.59(2.39-8.80)	0.91(0.25-3.30)	0.89
Resident					

Urban	216	71	2.52(1.60-3.96)	1.43(0.75-2.71)	0.281
Rural	64	53	1	1	
Monthly income					
<499	73	54	1	1	
500-2000	67	34	1.46(0.85-2.51)	1.272(0.52-3.09)	0.600
>2000	140	36	2.88(1.73-4.78)	1.29(0.46-3.60)	0.630
Training					
Yes	113	24	2.82(1.70-4.67)	1.74(0.89-3.42)	0.110
No	167	100	1	1	
Use social media					
Yes	204	48	4.25(2.72-6.65)	**2.96(1.46-6.01)***	**0.003***
No	76	76	1	1	
Peer					
Yes	252	93	3.00(1.71-5.27)	1.09(0.48-2.51)	0.840
No	28	31	1	1	
TV/radio					
Yes	243	78	3.87(2.34-6.40)	1.07(0.43-2.65)	0.88
No	37	46	1	1	
Religious institution					
Yes	98	32	1.55(0.97-2.48)	0.93(0.50-1.73)	0.83
No	182	92	1	1	

1, reference category

Table 6:-Associated factors of attitude towards COVID-19 prevention among adult visitors in hospitals of South Gondar zone, Northwest Ethiopia, in August 1 to 30, 2020

Variable	Attitude		COR (95% CI)	AOR(95%CI)	P-value
	Positive	Negative			
Age					
<20	12	12	1	1	
20-29	57	35	1.63(0.66-4.02)	0.42(0.12-1.46)	0.17
30-39	66	45	1.47(0.61-3.56)	0.51(0.14-1.82)	0.30
40-49	57	37	1.54(0.63-3.79)	0.94(0.28-3.21)	0.93
50-59	32	16	2.00(0.74-5.44)	1.10(0.28-4.27)	0.89
≥60	29	6	4.83(1.47-15.87)	1.65(0.33-8.42)	0.55
Religion					
Muslim	25	5	3.27(1.05-10.20)	2.18(0.50-9.58)	0.30
Orthodox	202	129	1.02(0.53-1.96)	1.49(0.64-3.48)	0.36
Protestant	26	17	1	1	
**Education**					
cannot read and write	22	44	1	1	
Read and write	47	48	1.96(1.02-3.76)	2.39(0.99-5.79)	.053
Primary	27	9	6.00(2.41-14.93)	**6.49(1.52-27.78)***	**0.012***
secondary	21	8	5.25(2.01-13.74)	2.32(0.39-13.74)	.35
College and above	136	42	6.48(3.49-12.01)	**6.91(2.58-14.50)***	**0.0001***
Occupation					
Farmer	22	46	1	1	
Student	36	9	8.36(3.44-20.36)	1.87(0.33-10.72)	0.48
Currently unemployed	33	22	3.14(1.50-6.58)	0.54(0.18-1.68)	0.29
Gov't worker	105	24	9.15(4.66-17.96)	0.61(0.12-3.05)	0.55
Private business	57	50	2.38(1.26-4.50)	0.29(0.07-1.12)	0.07
Resident					
Urban	193	94	1.95(1.26-3.02)	1.23(0.66-2.23)	0.51
Rural	60	57	1	1	
Monthly income					
<499	72	55	1	1	
500-2000	61	40	1.17(0.69-1.98)	0.89(0.33-2.38)	0.82
>2000	120	56	1.64(1.02-2.63)	0.57(0.19-1.70)	0.31
History of chronic illness					
Yes	48	8	4.19(1.92-9.12)	**5.00(1.71-14.67)***	**0.003***
No	205	143	1	1	
Training					
Yes	113	24	4.27(2.59-7.05)	**3.9(1.96-7.70)***	**0.0001***

No	140	127	1	1	
Use social media					
Yes	179	73	2.59(1.70-3.93)	1.20(0.59-2.44)	0.63
No	74	78	1	1	
Peer					
Yes	231	114	3.41(1.92-60.5)	**2.45(1.06-5.63)***	**0.04***
No	22	37	1	1	
Tv/radio					
Yes	221	100	3.52(2.13-5.81)	2.091(0.85-5.16)	0.11
No	32	51	1	1	
Religious institution					
Yes	100	30	2.64(1.64-4.23)	1.725(0.93-3.21)	0.09
No	153	121	1	1	

1, reference category

Associated factors of COVID-19 preventive practice

Table 7: Associated factors of preventive practice towards COVID-19 prevention among adult visitors in hospitals of South Gondar zone, Northwest Ethiopia, in August 1 to 30, 2020

Variable	Practice status		COR (95% CI)	AOR (95% CI)	P-value
	Good	Poor			
**Age**					
<20	9	15	1	1	
20-29	48	44	1.82(0.72-4.57)	0.87(0.24-3.38)	0.84
30-39	49	62	1.32(0.53-3.26)	0.43(0.12-1.67)	0.22
40-49	41	53	1.29(0.51-3.24)	0.75(0.20-2.56)	0.67
50-59	27	21	2.14(0.79-5.85)	0.99(0.23-4.24)	0.98
≥60	25	10	4.17(1.38-12.58)	1.31(0.28-6.11)	0.73
**Education**					
cannot read and write	17	49	1	1	
Read and write	25	70	1.03(0.50-2.11)	0.93(0.36-2.43)	0.88
Primary	16	20	2.31(0.98-5.44)	0.39(0.08-1.80)	0.23
Secondary	17	12	4.08(1.62-10.27)	0.82(0.16-4.19)	0.82
College and above	124	54	6.62(3.50-12.52)	1.90(0.67-5.17)	0.21
**Occupation**					
Farmer	6	62	1	1	
Student	24	21	11.81(4.25-32.83)	**7.70(1.15-15.86)***	**0.04***
Currently unemployed	20	35	5.91(2.17-16.08)	2.35(0.58-9.57)	0.23
Gov't worker	93	36	26.70(10.62-67.12)	2.49(0.42-14.61)	0.31
Private business	56	51	11.35(4.52-28.47)	2.15(0.45-10.2)	0.34
**Resident**					
Urban	166	121	3.49(2.19-5.56)	1.54(0.79-3.00)	0.21
Rural	33	84	1	1	
**Monthly income**					
<499	39	88	1	1	
500-2000	50	51	2.21(1.29-3.81)	2.05(0.71-5.93)	0.19
>2000	110	66	3.76(2.32-6.12)	1.99(0.62-6.39)	0.25
**Training**					
Yes	86	51	2.30(1.51-3.51)	0.88(0.47-1.64)	0.68
No	113	154	1	1	
**Use social media**					
Yes	160	92	5.04(3.23-7.87)	1.54(0.76-3.10)	0.23
No	39	113	1	1	
**Peer**					
Yes	184	161	3.35(1.80-6.25)	0.78(0.31-1.97)	0.61
No	15	44	1	1	
**Tv/radio**					
Yes	185	136	6.70(3.62-12.41)	1.45(0.53-3.96)	0.46
No	14	69	1	1	
**Knowledge**					

Poor knowledge	22	102	1	1	
Good knowledge	177	103	7.97(4.73-13.41)	**4.49(2.41-8.39)***	**0.0001***
**attitude**					
Negative attitude	44	107	1	1	
Positive attitude	155	98	3.85(2.50-5.93)	1.04(0.58-1.86)	0.068

1, reference category

Date: March 12 2021

Manuscript ID: PONE-D-20-36453R1

Preventive Practice and Associated Factors towards COVID-19 among Medical Visitors in Hospitals of South Gondar Zone, Northwest Ethiopia

Corresponding authors: Gete Berihun (MSc)

Dear Dr, Francesco Di Gennaro (PhD)
Academic Editor
PLOS ONE

Thank you for your letter dated January 27, 2021 with a decision of **major revision** needed. We were pleased to know that our manuscript was considered potentially acceptable for publication in PLoS ONE, subject to adequate revision as requested by the reviewers, academic editors and the journals. Based on the instructions provided in your letter, we uploaded the file of the rebuttal letter; the marked up copy of the revised manuscript highlighting the changes made in the original submitted version and the clean copy of the revised manuscript.

We have revised the manuscript by modifying the abstract, introduction, methods, results, discussion and other sections, based on the comments made by the reviewers and using the journal guidelines. Therefore, we have marked in red color all the changes made during the revision process. Appended to this letter is our point-by-point response (rebuttal letter) to the comments made by the reviewers.

We have agreed with almost all the comments and questions raised by the reviewers and academic editors. We also provided justification for those comments and questions for disagreeing. We would like to take this opportunity to express our thanks to the reviewers for their valuable comments and to thank you for allowing us to resubmit a revision of the manuscript.

I hope that the revised manuscript is accepted for publication in PLoS ONE.
Sincerely yours,
Gete Berihun (Wollo University)

Response to the Journal Requirements Questions

Response to editor

Question #1 Please ensure that your manuscript meets PLOS ONE's style requirements, including those for file naming.

Response: Thank you for this remark. We re-formatted the revised manuscript using the PLoS ONE format guidelines. The whole content of the manuscript, including the abstract, introduction, methods, discussion and reference are formatted using the guidelines (**Please see the revised version for each section**).

Question #2. Data availability

Response. We have attached the data on the supplementary information

Question #3. Please amend either the title on the online submission form (via Edit Submission) or the title in the manuscript so that they are identical.

Response: Thank you for your comment. We made the title identical on the online submission and the title in the manuscript.

Question #4. Please amend either the abstract on the online submission form (via Edit Submission) or the abstract in the manuscript so that they are identical.

Response: Thank you for your comment and we did it accordingly.

Question #5. Your ethics statement should only appear in the Methods section of your manuscript. If your ethics statement is written in any section besides the Methods, please move it to the Methods section and delete it from any other section. Please ensure that your ethics statement is included in your manuscript, as the ethics statement entered into the online submission form will not be published alongside your manuscript.

Response: Thank you. We did it.

Question #6. We note that you have included a table to which you do not refer in the text of your manuscript. Please ensure that you refer to Table 2 in your text; if accepted, production will need this reference to link the reader to the Table.

Response: We have accepted the comments and hence the text written in Table 1 is changed in to Table 2.

Response to reviewer 1

Question #1. What is the difference between patient and visitors in your study?

Response: Sorry for the confusion about the two terms. We understand that we used the two terms interchangeably and now we updated the manuscript by hospital visitors. the patient is all persons who came to health care facilities for medical treatment. But visitors are any persons who came to the health care facilities for different purposes including seeking of medical treatment. Therefore, to avoid confusion we used visitors since the study was done on visitors, not only to patients (see the revised version).

Question #2. The document has not page number please incorporate

Response: Based on your comment, we gave the page number accordingly. Thank you.

Question # 3. Some sentences are incomplete which need intensive editing.

Response: We tried to assess errors like incomplete sentence, grammatical and language error from title up to discussion of the manuscript. As a result, the amendment was done accordingly in the revised version of the manuscript.

Questions #4. In the abstract section please add space between 'of' and 'south' as 'To assess COVID-19 preventive practice and associated factors among visitors in 30 hospitals of South Gondar Zone, Northwest Ethiopia.

Response: Thank you for your comment. We made a correction in the revised manuscript.

Question #5. In abstract section, “The questionnaire was pre-tested in 5% of the final sample size to establish the validity of the data collection instrument. The data were collected using face-to-face interviews by considering physical distancing and wearing of face masks. The data was entered in Epi-data version 3.1 and exported to Statistical Package for Social Science (SPSS) Version 25 for analysis.” It is better delete and replace by tool of outcome variable measurement.

Response: Based on your comment we removed less important points from the abstract and amendment was done accordingly (see the revised new version of the manuscript).

Question #6. In abstract section, Bi-variate Crude Odd Ration (COR) with 95% confidence intervals (CI) and p-values of less than 0.25 were applied to select candidate variables for multi-variable analysis. Then, multi-variable Adjusted Odd Ratio (AOR) using binary logistic regression analysis at a p-value of less than 0.05 at 95% CI was shall be rephrased.

Response: Thank you for this key comment, we revised accordingly and please see the data analysis in page 9 and 10.

Question #7. In the abstract section, (AOR=2.96; 95% CI: 1.46, 6.01) were significantly associated with knowledge of COVID-1.’ This is incomplete and does not give sense. It is better rewrite it again.

Response: We have accepted your comment hence this sentence rewritten as …were associated significantly with visitors’ knowledge towards the prevention of COVID-19 (Please see the revised version of the abstract).

Question #8. In abstract section, the conclusion did not in line the finding. Please conclude according to the finding.

Response: We have accepted your comment. The conclusion was amended based on the finding of the study (see the revised version of the manuscript).

Question #9. In Methods and Materials section, the patient flow data were estimated by reviewing the patients' logbook in the last three months and the average number of the patient for a month was calculated to determine the interval. Then, we used a systematic random sampling

technique to select study participants of the study'. This paragraph is not clear. How to reach to apply systematic random sampling technique? What is the sampling frame? Is your study population are patients or patients attendance or any visitor of the hospital?

Response: We found that our way of writing was confusing. Sorry for the mistakes. After selecting the two hospitals randomly out of the 8 hospitals, we proportionally allocated sample size based on total estimated visitors of hospitals in the last three months. Then, 303 sample size was allocated for Debre Tabor general hospitals and 117 for Mekane Eyesus hospitals. Then hospitals visitors flow data during the previous 3 months in emergency ward, surgical ward, medical ward, gynecology/obstetrics ward and pediatrics ward considered for sample size allocation for each hospitals departments. Finally, randomly selection of visitors for each ward was selected until the allocated sample size was achieved (See the revised version in page 7.

Question #10. Under outcome and explanatory variables: please delete sensitive words like poor knowledge, attitude, and practice. It shall be replaced with 'favorable/unfavorable'

Response: We accepted your reflections, however, most studies used good/poor knowledge, positive and negative attitude and good/poor practice. We used these terms accordingly throughout the paper.

Question #11. Delete subtopic of 'operational definition.' This is already state in the outcome variables. Please avoid bolding words like 'good knowledge, poor knowledge, positive attitude, and Practice

Response: Thank you for your comment; we deleted subtopic of operational definition

Question#12. A pre-test was conducted using 5% (21) of the final sample size in the Andabet district to establish the validity of the questionnaire and amendment was made accordingly.' What type of amendment you made? Can you explain that amendment?

Response: The pre-test is aimed for amendment of measuring tool. Therefore, some of the amendments were arrangement, editing of unclear questions, and avoiding irrelevant questions.

Question 13. Under Statistical analysis: what is the different between bi- variate and multi-variable? What do you mean 'multi-variable'?

Response: Bivariate analysis refers one independent variable with outcome variables. However, multivariable means that more than one independent variables with the outcome variable. From the adjusted analysis, all variables that has a p-value less than 0.25 were included into the adjusted multivariable analysis to control confounders. In our study, in the case of this study we used bivariable and multivariable analysis. Furthermore, the word bivariate analysis was changed to bivariable analysis throughout the manuscript.

Question#14. Result section, use one of result presentation. Almost all tables are explained in the text. Please follow rule of text and table presentation together.

Response: Based on your comments we reduced more than half of the explanation. As a result, only pertinent finding of the study was explained (Please see all result section).

Question #15. In result section, similarly, 283 (70%) of the respondents knew that COVID-19 can be transmitted from one person to another even in the absence of COVID-19 (Table 1).' Table 1 presents socio-demographic characteristics of the participants but not knowledge of participants. Please cite the table appropriately.

Response: We made a correction accordingly (See the revised version of the manuscript).

Question #16. The finding of the study revealed seven out of ten 280 (69.3%) respondents had good knowledge towards COVID-19 while 253 (62.6%) had a positive attitude towards COVID-19. The finding of the study showed the pillar of prevention practice was much lower and only

half 199(49.3%) of the participants had a score of good prevention practice of COVID-19 (Figure 1).' This paragraph is not clear.

Response: This idea is rewritten as'...280 (69.3% of study participants had favorable knowledge towards COVID-19 prevention. '... almost half of the study participants 119 (49.3%) are practiced the recommended COVID-19 prevention methods.'

Question #17. In result section, the finding revealed that those who can read and write were 2.78 times more likely to have good knowledge than those who can't read and write is not clear and should be re-write.

Response: We have rewritten as "The finding revealed that those who can read and write were 2.78 times more likely to have good knowledge towards COVID-19 prevention methods than those who can't read and write".

Question #18. In discussion section, this discrepancy 274 might be due to Spatio-temporal variation.' This is not a justifiable reason to the knowledge people towards novel coronal virus discrepancy of between Ethiopia and Egyptian population. Please search another justification of this discrepancy.

Response: Based on comment we tried to elaborate better justification for variation in this study with the finding of other researches conducted in different parts of the world.

Question #19. In discussion section, this deviation may be due to the change in the study population (health care professionals vs. general population) and residents of the study population. This makes confuse reader please rephrase again

Response: This idea is rewritten as" the deviation may be due to the difference in the study subjects. In the present study, the study subjects were visitors of Hospitals while the study conducted in Egypt were only health care professionals.

Response to reviewer 2

Question #1. The outcome variable and the title is not congruent. If your outcome variables are knowledge, attitude and practice towards COVID -19 prevention, your title should be modified to KAP of visitors towards COVID-19 prevention. Otherwise, if your title is focused only practice and associated factor, you have to include knowledge and attitude as part of associated factor, rather than the outcome variable.

Response: Based on the comment, we tried to make the title in line with its outcome variable. Therefore the title is modified in to KAP of visitors towards COVID-19 prevention while the outcome variables of the study are Knowledge, attitude, and practice towards COVID-19 prevention

Response to reviewer 3

Question #1. Result and discussion part 1. Please use software modeling for clear elaboration the topic of CVID 19 Examples OLS Model, add other better software

Response: We have already used logistic regression analysis using crude odds ratio (COR) and adjusted odds ratio (AOR) for determining the associated factors with the outcome variables. Associations between independent variables and knowledge, attitudes and practices towards COVID-19 were determined using a binary logistic regression model at 95% CI (Confidence interval). We used three different logistic regression models: The first model (Model 1) identified factors associated with good knowledge about COVID-19, the second model (Model II) identified factors associated with favorable attitudes and the third model (Model III) identified factors associated factors with good preventive practices towards COVID-19. For each model, bivariable analysis with (COR [crude odds ratio]) and multivariable analysis (AOR [adjusted odds ratio]) was used.

From the bivariable analysis, variables with a *p*-value <0.25 were retained into the multivariable logistic regression analysis. From the multivariable analysis of each model, variables with a

significance level of *p*-value <0.05 were taken as factors independently associated with knowledge, attitude and practices towards COVID-19. The presence of multicollinearity among independent variables was checked using standard error at the cutoff value of 2 and we found that a maximum standard error of 0.97, which indicated no multi-collinearity. Model fitness was checked using the Hosmer-Lemeshow test for Model I, Model II and Model III and found a *p*-value of 0.650, 0.871 and 0.913, respectively and indicated that all models were fit.

We hope that the data analysis we used above is very sufficient to our study, which we could able to explain the result and discussion as we did it.

Question #2. Adding Images as possible

Response: We have 7 Tables and including more Figure is repeating of the result of the Table in another forms.

Question #3. Edit grammatical error and other

Response: This comment was also raised by other reviewers. We tried to revise of grammar, language, and punctuation errors starting from the title of the manuscript up to discussion (see the revised version of the manuscript). We appreciate your comment.

Response to reviewer 4

Question #1. What does medical visitor mean? is that for only visiting the medical ward? if not, it is better to say among visitors.

Response: The study subjects all visitors of the health care facilities. So that, the title is modified to Knowledge, Attitude, and Practices towards COVID-19 and associated factors among hospital Visitors in South Gondar Zone Hospitals, Northwest Ethiopia

\Question #2. In the background section, please also include information on preventive practices of COVID-19.

Response: The title of the revised is modified to Knowledge, Attitude, and Practices towards COVID-19 and associated factors among hospital Visitors in South Gondar Zone Hospitals, Northwest Ethiopia. Therefore the emphasis is given not only for prevention measures but also for knowledge and attitude towards prevention of COVID-19. But in the revised manuscript, we elaborate detail COVID-19 prevention measures.

Question #3. Replace the word "face-to-face administered" by "interviewer administered"

Response: The phrase "face-to-face administered" is replaced by "interviewer administered". (See the revised version of the manuscript).

Question #4. Please include the confidence intervals for these percentages with 95% CI.

Response: Knowledge, attitude, and practice of respondents are presented in the result and discussion section of the revised manuscript. According to this study, 69.3% (95%CI;65.1-73.8%) had a favorable knowledge, 62.6% (95%CI;57.2-67.6) had a favorable attitude, and 49.3%(95%CI;) had a favorable practice towards the prevention of COVID-19.

Question #5. If you did the associations for knowledge, attitude and practice of visitors towards COVID- 19 prevention, please rewrite your title as knowledge, attitude and practice of hospital visitors towards COVID-19 prevention....

Response: We have accepted your comment and the title was modified accordingly.

Question #6. Bivariate analysis refers two paired data sets/with two outcome variables. But your study has single outcome so, better to use bivariable.

Response: The word bivariate analysis was replaced by bivariable analysis throughout the revised version of the manuscript.

Question #7. Where does your pretest conducted and what are the psychomotor properties of that pretest? Particularly state clearly in your methods section, than the abstract.

Response: the pretest was conducted in other districts hospital visitors (andabet hospital) other than the study area. The psychomotor domains of the pretest are reliability and validity.

Question #8. How the response rate becomes 95.7%, if you use interviewer administered questionnaire?

Response: As we know the response rate of interviewer administered questionnaire is higher than self-administered once. But in the case of our study, the study subjects were visitors of health care facilities who came for different activities including medical treatments. The non-response rate of the study participants means that those study participants who are unable to provide data due to shortage of time, unwillingness to be part of the study and etc.

Question #9. Most paragraphs emphasized what COVID -19 entails and may be considered as too lengthy. Introduction should follow a structured and sequential order while capturing relevant information to be included in this section. What are the current issues about COVID -19 prevention? Some of these points were included in one of your paragraphs. What are the quantifiable effect/issue around the points identified on COVID- 19 prevention? Based on the earlier identified issues about COVID-19 prevention, what are the points to be addressed in your study? What is the rationale for the study? and lastly what is the aim of the study?

Response: We thank you for this key comment. Based on your comment we tried to shorten the introduction part. Furthermore we point out the current issues of COVID-19 prevention measures, earlier identified COVID-19 prevention measures, points to be addressed, rational of the study and finally aims of the study are explained in the revised version of the manuscript (See the updated manuscript).

Question #10. If you exclude individuals whose age is <18 years , it is better to modify your title as ... among adult visitors... otherwise, why you exclude these groups?

Response: Based on your comment we modified the title as to only adult visitors.

Question #11. You stated the total population of South Gondar Zone. However, it is better to state the average number of monthly visitors to hospitals within the zone because you are aiming to study visitors...

Response: we tried to modify this point method section particularly in study design, setting, and period of the revised version of the manuscript.

Question #12. If these are your outcome variables, your title should be revised as KAP (knowledge, attitude, and practice of visitors towards COVID-19 prevention...) otherwise use practice as your outcome variable and use knowledge, attitude, socio-demographic.... as your independent variables. Doing research without knowing the variables is meaningless.

Response: The title of the manuscript are modified in to KAP of adult visitors towards prevention of COVID- 19 to make in line with the outcome variable of favorable/ unfavorable knowledge, attitude, and practice towards prevention of COVID-19.

Question #13. Sampling procedure is not clear

Response: Sorry for the confusion. We rewrite the sampling procedure in a more clear manner. After selecting the two hospitals randomly out of the 8 hospitals, we proportionally allocated sample size based on total estimated visitors of hospitals in the last three months. Then, 303 sample size was allocated for Debre Tabor general hospitals and 117 for Mekane Eyesus hospitals. Then hospitals visitors flow data during the previous 3 months in emergency ward, surgical ward, medical ward, gynecology/obstetrics ward and pediatrics ward considered for sample size allocation for each hospitals departments. Finally, randomly selection of visitors for each ward was selected until the allocated sample size achieved.

Question #14. Attitude measurement is not clear. What does 26.4 (80%) score mean? is that the mean or median score of the overall attitude score?

Response: The attitude of the participants was measured using 11 items based three measurement scales with agree (3 points), neutral (2 points), and disagree (1 point). As a result the score varied from 11 to 33. Therefore, respondents with a mean score of ≥27 (80%) were considered as having a favorable attitude towards the prevention of COVID-19.

Question #15. Conducting of pre-test and keeping of the recommended physical distances should be considered as parts of ethical consideration rather than data collection.

Response: Based on the comment we moved the statement of keeping recommended physical distance during data collection to ethical consideration from method section (Please see the ethical consideration section).

Question #16. Is it feasible to conduct double data entery?

Response: We have written in a different term what we did, which is wrong. Thank you for your commitment in brining such errors for correction. We mean that and what we did was data entry was re-checked for 10% of the sample size in order to control data entry errors of the entered data and data cleaning was carried before statistical analysis. Thank you so much.

Question#17. The overall knowledge of the respondents should also be stated in figures, percentages using 95% CI based on the operational definitions stated in the methods section.

Response: Based on the operational definition, the knowledge of the respondents' was presented using figures and percentages with 95%CI. Therefore, about 280 (69.3%) (CI; 65.1-73.8) of the participants had a good knowledge towards prevention of COVID-19.

Question #18. Your sample size is 422. However, you collect from 404 participants alone. Why? None response rate is rare in interviewer administered/ face to face questionnaire. Why this discrepancy arises?

Response: The response rate of the study was 95.7%. The response rate of interviewer administered questionnaire is higher than self-administered once. But in the case of our study, the study subjects were visitors of health care facilities who came for different activities including medical treatments. The non-response rate of the study participants means that those study participants who are unable to provide data due to shortage of time, unwillingness to be part of the study and etc. Since the study subjects were all visitors of the healthcare facilities who came for different activities including medical treatment. Therefore they withdraw from the interview for different personal reasons which made the non-response rate higher than the expected one. Of course the 95.% response rate is good for such type of study.

Question #19. The heading of Status of Knowledge, attitude, and preventive practice of COVID-19**.** It is better to avoid this title and write in their own parts as I comments above.

Response: Based on your comment we omitted this subheading and the contents were written in their own parts accordingly.

Question #20. Predictor is used for more advanced studies like cohort study. In this cross - sectional study, it is better to say associated factors.

Response: Yes, this is excellent idea too. The word predictor is replaced by associated factors throughout the revised version of the manuscript.

Question #21. Discuss only your pertinent findings like knowledge, attitude, and practice rather than discussing on sources of information towards COVID-19.

Response: Thank you very much for your comment. We tried to discuss only the pertinent finding of knowledge, attitude, and practices towards prevention of COVID-19. As a result, other less important points like sources of information are removed in the revised version of the manuscript.

Question #22. Why your justification becomes similar throughout your discussion? Please give reasonable justifications for each

Response: We tried to write specific justification for each variation in the finding of this study with other study finding

Question #23. Conclude based on your objective. Please also include the major factors affecting practice in the conclusion section. Finally, your recommendations should be based on your results. Does it mean, there is no problem on knowledge and attitude? Please rewrite it

Response: In the original version of the manuscript our emphasis was only on prevention practice of COVID-19 rather than knowledge and attitude of visitors towards COVID-19 prevention measures. But now the title is modified in to knowledge, attitude, and practice. Therefore the conclusion is amended according to finding of the study. (See the revised version of the manuscript.

Question #24. Avoid variables which contain a confidence interval of 1 in binary logistic regression.

Response: All variables in logistic analysis which contains a confidence interval of 1 are excluded

: Question #25. Check the figure digits

Response: We have accepted the comment and all figure digits are presented with two digit value

I hope that the revised manuscript is accepted for publication in PLoS ONE.

Sincerely yours,

Gete Berihun

Department of Environmental Health
Wollo University
Dessie, Ethiopia.
