# Supplementary material for: Knowledge, attitude, and preventive practices towards COVID-19 and associated factors among adult hospital visitors in South Gondar Zone Hospitals, Northwest Ethiopia
Source: PLoS One. 2021 May 17;16(5):e0250145. doi: 10.1371/journal.pone.0250145 (PMC8128268; doi:10.1371/journal.pone.0250145)
Supplement: Attachment — Submitted filename: plos review.docx [file pone.0250145.s004.docx]

Dear Editor,

I carefully read the article by Barium et al., which is interesting a quite well done.

My remarks are the following:

General comment

- What is the difference between patient and visitors in your study?
- The document has not page number please incorporate
- Some sentence are incomplete which need intensive editing

Abstract section

1. Objective

….please add space between ‘of’ and ‘south’ as ‘To assess COVID-19 preventive practice and associated factors among visitors in 30 hospitals **of South** Gondar Zone, Northwest Ethiopia.’ It is also page 2 line 42 please add space.

2. Method

- … It say “The questionnaire was pre-tested in 5% of the final sample size to establish the validity of the data collection instrument. The data were collected using face-to-face interviews by considering physical distancing and wearing of face masks. The data was entered in Epi-data version 3.1 and exported to Statistical Package for Social Science (SPSS) Version 25 for analysis.” It is better delete and replace by tool of outcome variable measurement.
- ‘Bi-variate Crude Odd Ration (COR) with 95% confidence intervals (CI) and p-values of less than 0.25 were applied to select candidate variables for multi-variable analysis. Then, multi-variable Adjusted Odd Ratio (AOR) using binary logistic regression analysis at a p-value of less than 0.05 at 95% CI was….’shall replace this paragraph by this ‘*Logistic regression was applied to assess the association between dependent and explanatory variables. The association was interpreted using the adjusted odds ratio (AOR) and 95% confidence interval* *(CI)’.*

3. Result

- ‘Multi-variable (AOR) logistic
- …….. (AOR=2.96; 95% CI: 1.46, 6.01) were significantly associated with knowledge of COVID-1.’ This is incomplete and does not give sense. It is better rewrite as ‘……*were associated significantly with visitors’ knowledge about prevention of COVID-19*.’ The same is true for altitude.
- Line 53-54 says…… good knowledge on COVID-19 (AOR=4.49; 54 95% CI: 2.41-8.39) were significantly associated. Does not make sense please add subject and verb like *participant who had good knowledge* ….

4. Conclusion

- The conclusion did not in line the finding. Please conclude according to the finding.

Methods and Materials section

1. Line 34-37 ‘The patient flow data were estimated by reviewing the patients' logbook in the last three months and the average number of the patient for a month was calculated to determine the interval. Then, we used a systematic random sampling technique to select study participants of the study’. This paragraph is not clear. How to reach to apply systematic random sampling technique? What is the sampling frame? Is your study population are patients or patients attendance or any visitor of the hospital?
2. Under outcome and explanatory variables: please delete sensitive words like poor knowledge, attitude, and practice. It shall be replaced with ‘*favorable/unfavorable’*
3. Delete subtopic of ‘operational definition.’ This is already state in the outcome variables. Please avoid bolding words like ‘**good knowledge, poor knowledge, positive attitude, and Practice.’**
4. Line 65 and 66 **‘**A pre-test was conducted using 5% (21) of the
   final sample size in the Andabet district to establish the validity of the questionnaire and amendment was made accordingly.’ What type of amendment you made? Can you explain that amendment?
5. Under Statistical analysis line 77-78: what is the different between bi- variate and multi-variable? What do you mean ‘multi-variable’?

Result section

1. Use one of result presentation. Almost all tables are explained in the text. Please follow rule of text and table presentation together.
2. Line 208 & 209: ‘Similarly, 283 (70%) of the respondents knew that COVID-19 can be transmitted from one person to another even in the absence of COVID-19 (Table 1).’ Table 1 presents socio-demographic characteristics of the participants but not knowledge of participants. Please cite the table appropriately.
3. Line 234-238: ‘The finding of the study revealed seven out of ten 280 (69.3%) respondents had good knowledge towards COVID-19 while 253 (62.6%) had a positive attitude towards COVID-19. The finding of the study showed the pillar of prevention practice was much lower and only half 199(49.3%) of the participants had a score of good prevention practice of COVID-19 ***(Figure 1).’*** This paragraph is not clear*.* This is better rewrite as’...*280 (69.3% of study participants had favorable knowledge towards COVID-19 prevention. ‘... almost half of the study participants 119 (49.3%) are practiced the recommended COVID-19 prevention methods*.’
4. Line 242-247: this paragraph is not clear. The sentence are not provide full information. For example ‘The finding revealed that those who can read and write were 2.78 times more likely to have good knowledge than those who can‘t read and write.’ To more clear rewrite as *“The finding
   revealed that those who can read and write were 2.78 times more likely to have good knowledge towards COVID-19 prevention methods than those who can‘t read and write.” The same is true for others and the next paragraph also.*
5. *Line 273 &274: ‘*This discrepancy 274 might be due to Spatio-temporal variation.’ This is not a justifiable reason to the knowledge people towards novel coronal virus discrepancy of between Ethiopia and Egyptian population. Please search another justification of this discrepancy.
6. Line 279 & 280: This deviation **may be due** to the change in the study population (health care professionals **vs.** general population) and residents of the study population. This make confuse reader please rephrase again
