# Supplementary material for: Knowledge, attitude, and preventive practices towards COVID-19 and associated factors among adult hospital visitors in South Gondar Zone Hospitals, Northwest Ethiopia
Source: PLoS One. 2021 May 17;16(5):e0250145. doi: 10.1371/journal.pone.0250145 (PMC8128268; doi:10.1371/journal.pone.0250145)
Supplement: Attachment — Submitted filename: coment.docx [file pone.0250145.s007.docx]

**Manuscript Title:** Knowledge, Attitude, and Practices towards COVID-19 and associated factors among adult hospital Visitors in South Gondar Zone Hospitals, Northwest Ethiopia

Topic sound

Mereology: fit scientific standard

Discussion fit the order of scientist criteria

Conclusion fit the scientific criteria
